# Design, synthesis, and evaluation of 1,4-benzothiazine-3-one containing bisamide derivatives as dual inhibitors of *Staphylococcus aureus* with plausible application in a urinary catheter

**DOI:** 10.3389/fchem.2024.1420593

**Published:** 2024-06-26

**Authors:** Kaushal Naithani, Arka Das, Mamta Ushare, Subham Nath, Rashmita Biswas, Anirban Kundu, Kazi Tawsif Ahmed, Utpal Mohan, Subhendu Bhowmik

**Affiliations:** ^1^ Department of Medicinal Chemistry, National Institute of Pharmaceutical Education and Research, Kolkata, West Bengal, India; ^2^ Microbiology Division, Department of Medicinal Chemistry, National Institute of Pharmaceutical Education and Research, Kolkata, West Bengal, India; ^3^ Department of Natural Product, National Institute of Pharmaceutical Education and Research, Kolkata, West Bengal, India; ^4^ Department of Botany, Visva Bharati University, Santiniketan, West Bengal, India

**Keywords:** benzothiazine, *Staphylococcus aureus*, peptide deformylase, biofilm, catheter, computational studies

## Abstract

In this study, 1,4-benzothiazine-based bisamide derivatives, a new class of antibacterial agents targeting bacterial peptide deformylase (PDF), were designed and synthesized to combat *Staphylococcus aureus* infection. Molecular modeling of the designed molecules showed better docking scores compared to the natural product actinonin. Bioactivity assessment identified two derivatives with promising antibacterial activity *in vitro*. The stability of the most active molecule, **8bE**, was assessed using molecular dynamics (MD) simulation. Significantly, compound **8bE** could also inhibit the *S. aureus* biofilm at low concentrations. Furthermore, the capability of the synthesized molecule to inhibit *S. aureus* biofilm formation on medical devices like urinary catheters is also demonstrated.

## 1 Introduction

*Staphylococcus aureus*, a Gram-positive bacterium that is mostly located in the upper respiratory tract and skin of healthy humans, is the major cause of nosocomial infection and is associated with significant mortality among hospitalized patients (Willekens et al., 2021). The major issue regarding *S. aureus* is its ability to form biofilms and grow on the surface of medical equipment like urinary catheters, which further increases the chances of infection among immune-compromised patients (de Oliveira et al., 2021). Catheter-associated UTIs (CAUTIs) are among the most frequent types of hospital-acquired infections and uropathogens like *S. aureus* (Walker et al., 2017). Biofilms are complex clusters of microorganisms produced by bacteria, providing the pathogens several advantages like resistance to phagocytosis and antimicrobial agents (Vashistha et al., 2023). On urinary catheters, the biofilms add another advantage to the bacteria as they provide resistance to the bacteria against the sheer force of the urine flow (Werneburg, 2022). Therefore, the development of antibacterial agents that can inhibit bacterial biofilm formation on medical equipment is of utmost need.

**FIGURE 1:**
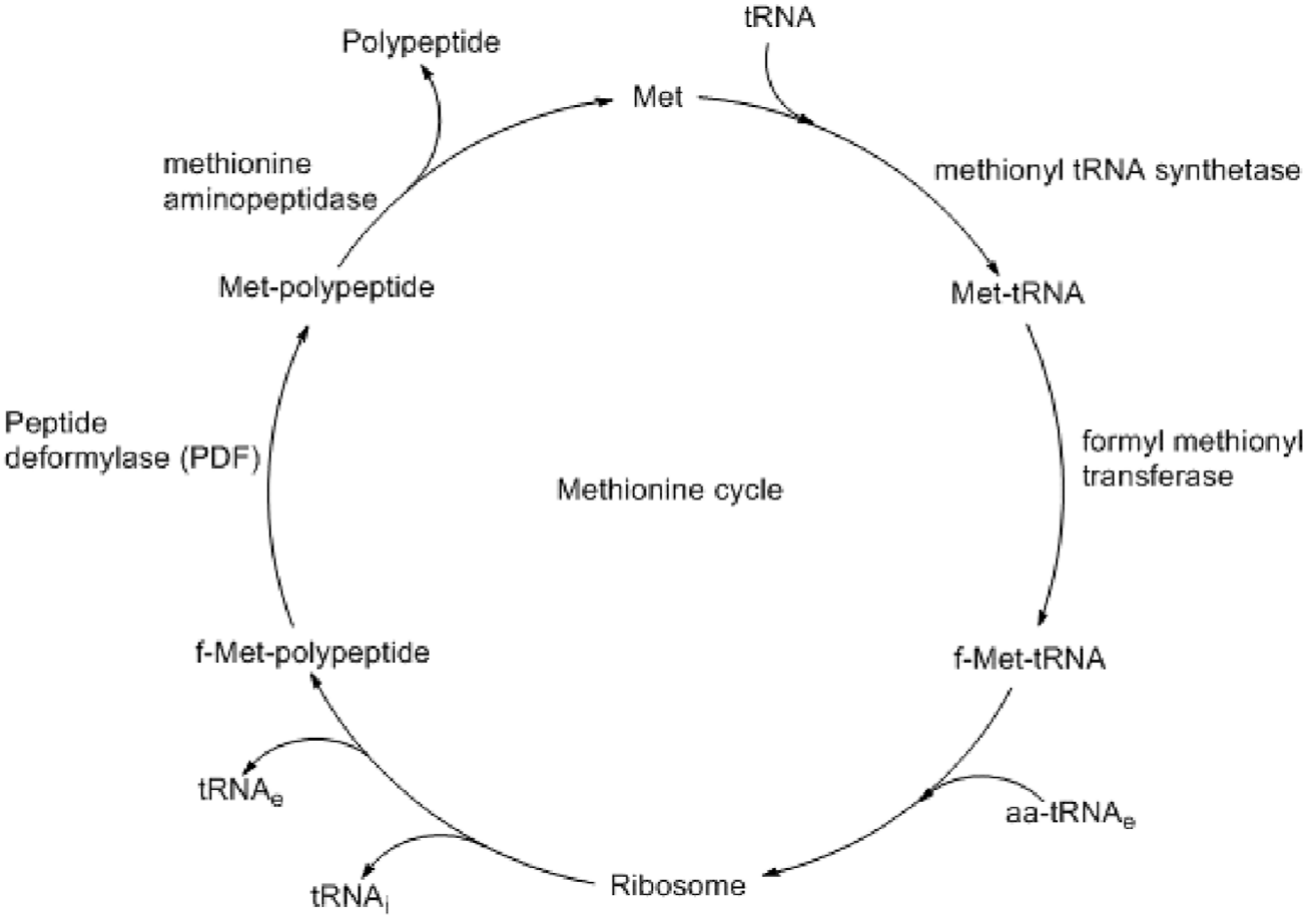
Methionine cycle shows the important role of peptide deformylase (PDF) in polypeptide synthesis (Aubart and Zalacain, 2006).

Bacterial biofilm formation and pathogenesis are controlled by several enzymes like fibronectin-binding anchors and collagen-binding proteins (Foster et al., 2014). Some are responsible for virulence, and some are responsible for defense by biofilm formation. Although very challenging, the development of therapeutics by targeting both virulence and biofilm formation could be an effective option in controlling bacterial infection. Peptide deformylase (PDF) is one of such proteins present at the *def* gene of *S. aureus* and catalyzes the deformylation step during protein synthesis (Margolis et al., 2000). During bacterial protein synthesis, N-formyl methionine, which is formed by formyl methionine tRNA transferase, is removed by PDF (Figure 1) (Aubart and Zalacain, 2006). Such a formylation–deformation cycle is essential for the growth and survival of all bacterial species, including *S. aureus* (Yang et al., 2014). Different studies also found that similar to the virulence proteins, the biosynthesis of biofilm-associated protein (Bap) also depends on such formylation–deformation steps, as controlled by PDF (Swarupa et al., 2018). Therefore, targeting PDF may prevent bacterial virulence and biofilm formation simultaneously.

**FIGURE 2:**
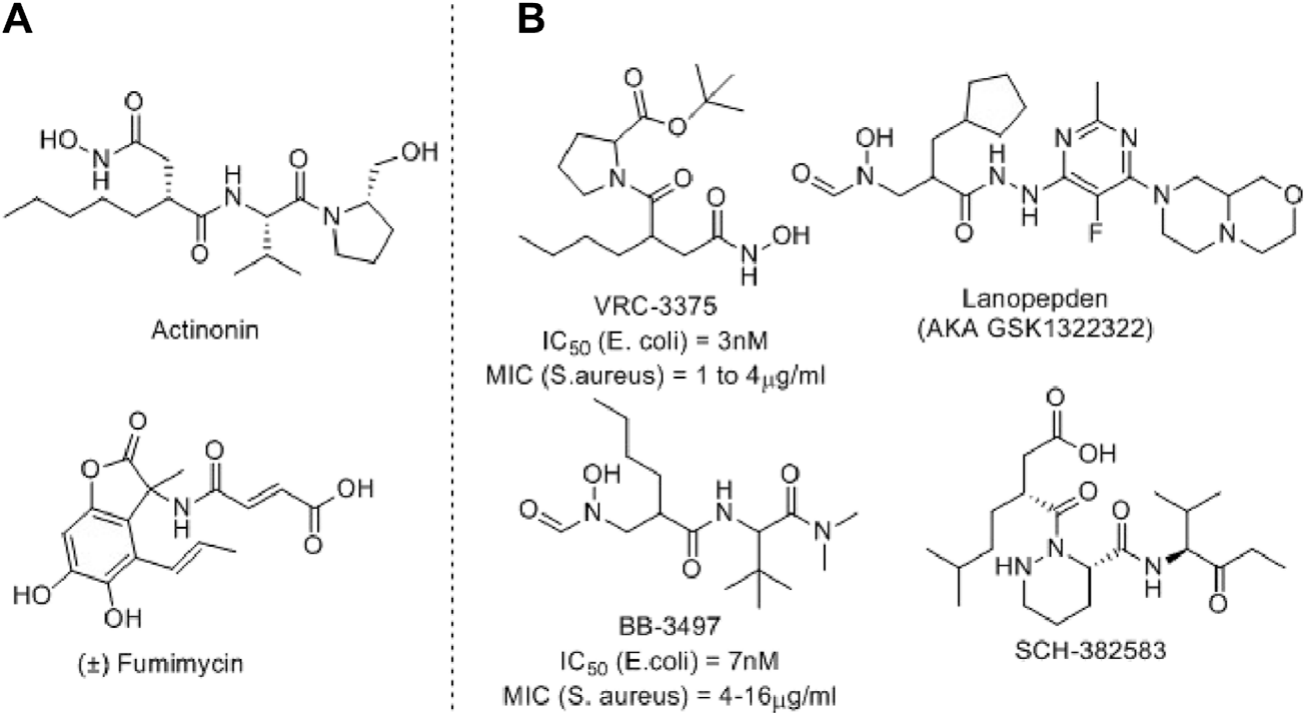
Representative examples of PDF inhibitors. **(A)** Naturally Occurring PDF inhibitors **(B)** PDF Inhibitors in clinical trials.

Several PDF inhibitors have been developed so far using the natural product actinonin as a prototype, although none have been marketed (Figure 2). In all these known PDF inhibitors, the characteristic feature is the metal-binding hydroxamic or *N*-formylated or free acid site as the metal-binding motif (Yang et al., 2014). However, the robust metal-binding affinity of these groups creates toxicity by binding with the iron present in blood and, as a result, causes methemoglobinemia (Gokhale and Telvekar, 2021). To overcome this, other groups have developed thiol (Belete, 2019), *N*-substituted maleamic acid (Zaghouani et al., 2019), or peptide-based (Hu et al., 2004) PDF inhibitors, although with limited success. Keeping this in mind, herein, we designed a series of benzothiazine-3-one-based bisamide derivatives as plausible PDF inhibitors.

Benzothiazines are privileged scaffolds with numerous activities, including antibacterial, antifungal, and anticancer properties (Ahmad et al., 2014). Depending on the positioning of *N* and *S*-atoms, these scaffolds are classified as 1, 2-, 1,3-, or 1,4-benzothiazines. The 1,4-benzothiazines have antibacterial, antifungal, anticancer, and several other activities (Badshah and Naeem, 2016). Furthermore, 1,4-benzothiazine-3-one containing hydroxamic acids also displayed good bacterial Ni-PDF inhibitory activity (Molteni et al., 2004). Considering such biological significance and known PDF inhibitory roles, we designed a 1,4-benzothiazine-3-one-containing molecule where the hydroxamic acid part is replaced with a bisamide side chain.

## 2 Materials and methods

### 2.1 Experimental section

Solvents were dried using standard procedures or collected from a solvent purification system (SPS). All starting materials were obtained from commercial suppliers and used as received. Products were purified by flash chromatography on silica gel (230–400 mesh, Merck). ^1^H and ^13^C NMR spectra were recorded using JEOL-400-MHz instruments. Signals are quoted as δ values in ppm using residual protonated solvent signals as the internal standard (CDCl_3_: δ 7.26 ppm). Data are reported as follows: chemical shift, multiplicity (*s* = singlet, *d* = doublet, *t* = triplet, *q* = quartet, *m* = multiplet, and bs = broad), coupling constants (Hz), and integration. The HRMS spectra were recorded as EI-HRMS (recorded as ESI+) using Q-TOF YA263 high-resolution (Water Corporation) instruments.

### 2.2 Chemistry

#### 2.2.1 Synthesis and characterization

##### 2.2.1.1 2-(3-Oxo-3,4-dihydro-2*H*-benzo[*b*][1,4]-thiazin-2-yl)-acetic acid (3)

Maleic anhydride (**2**) (4.3 g, 43.93 mM, 1.1 eq) was dissolved in toluene (∼15 mL) and stirred until a clear solution was obtained. Then, 2-amino-thiophenol (**1**) (5 g, 39.94 mM, 1 eq) was added. The reaction was stirred for 6 h until an off-white precipitate was obtained. The precipitate was filtered off and washed with CHCl_3_. It was dried at room temperature to remove traces of CHCl_3_. The precipitate was used without further purification. The desired compound was obtained as a white solid in 82% yield (7.13 g). Percentage yield = 82% as an off-white solid, R_f_ = 0.1 (hexane:EtOAc, 1:1); ^1^H NMR (400 MHz, CD_3_OD): δ 7.29 (d, *J* = 7.8 Hz, 1H), 7.18 (t, *J* = 7.6 Hz, 1H), 7.02 (t, *J* = 7.6 Hz, 1H), 6.97 (d, *J* = 7.8 Hz, 1H), 3.86 (dd, *J*_1_ = 8 Hz, *J*_2_ = 6.4 Hz, 1H), 2.91 (dd, *J*_1_ = 16.4 Hz, *J*_2_ = 6.3 Hz, 1H), and 2.52 (dd, *J*_1_ = 16.4 Hz, *J*_2_ = 6 Hz, 1H); ^13^C NMR (100 MHz, CD_3_OD): δ 173.5, 168.6, 137.9, 129, 128.5, 124.9, 120.2, 118.4, 39.3, and 34.9 (Molteni et al., 2004).

##### 2.2.1.2 General procedure for the synthesis of 8(aA–kG)

An amine derivative was dissolved in 5 mL reagent grade methanol and stirred until a clear solution was obtained. To this solution, carboxylic acid derivative, **3**, was added, resulting in a suspension. This suspension was cooled to 5°C, and *tert*-butyl isocyanide, **7**, was added. To this mixture, an aldehyde derivative was added, and the reaction was stirred at room temperature (∼30°C) overnight.

NOTE: Reactions involving furfural aldehyde, tryptamine, or phenylethyl amine require a temperature of 60°C for the completion of the reaction.

Workup: The reaction mixture was evaporated until a solid layer was obtained. It was then dissolved in CHCl_3_ and washed with distilled water (3*15 mL), followed by a brine solution (2*15 mL). The organic layer was then collected and dried using sodium sulfate. It was then evaporated in a rotavap until a thin film was obtained.

##### 2.2.1.3 *N*-benzyl-*N*-[2-(*tert*-butylamino)-2-oxo-1-phenylethyl)-2-(3-oxo-3,4-dihydro-2*H*-benzo [*b*][1,4]-thiazin-2-yl]-acetamide (8aA)

The reaction was carried out as mentioned for the synthesis of 8. The desired compound was obtained as a pale-brown solid in 60% yield (135 mg), R_*f*_ = 0.3 (hexane:EtOAc, 7:3); ^1^H NMR (400 MHz, CDCl_3_): δ 8.77 (bs, 1H), 7.39–7.29 (m, 3H), 7.23–7.2 (m, 3H), 7.15–7.06 (m, 4H), 7.03–6.92 (m, 2H), 6.85–6.81 (m, 2H), 6.01–5.86 (m, 2H), 4.79–4.47 (m, 2H), 4.24–4.19 (m, 1H), 3.08–2.9 (m, 1H), 2.63–2.43 (m, 1H), 1.33 (s, 9H); ^13^C NMR (100 MHz, CDCl_3_): δ 171.1, 168.7, 167.5, 137.1, 136, 134.8, 129.7, 128.5,128.3, 128, 127.2, 126.8, 126, 125.8, 123.8, 119.7, 117.2, 63.2, 51.6, 49.8, 38.5, 33.4, and 28.6; ES + HRMS calculated for C_29_H_31_N_3_O_3_SNa = 524.1978 and obtained = 524.1997.

##### 2.2.1.4 *N*-(4-Bromophenyl)-*N*-(2-(*tert*-butylamino)-2-oxo-1-phenylethyl)-2-(3-oxo-3,4-dihydro-2*H*-benzo[*b*][1,4]-thiazin-2-yl)-acetamide (8aC)

The reaction was carried out as mentioned for the synthesis of 8. The desired compound was obtained as a pale yellow solid in 54% yield (137 mg), R_*f*_ = 0.3 (hexane:EtOAc, 7:3); ^1^H NMR (400 MHz, CDCl_3_): δ 8.67 (s, 1H), 7.68 (s, 1H), 7.26 (dd, *J* = 7.79 Hz, 1.30 Hz, 1H), 7.20–7.10 (m, 6H), 6.98 (td, *J*_1_ = 7.60 Hz, *J*_2_ *=* 1.27 Hz, 1H), 6.82 (dd, *J*_1_ = 7.95 Hz, *J*_2_ = 1.07 Hz, 1H), 6.21–6.75 (m, 2H), 5.94–6.05 (m, 1H), 5.76–5.91 (m, 1H), 4.15 (dd, *J* = 8.02 Hz, 6.19 Hz, 1H), 2.76 (dd, *J* = 16.20 Hz, 6.19 Hz, 1H), 2.26 (q, *J* = 8.07 Hz, 1H), 1.31–1.36 (s, 9H); ^13^C NMR (100 MHz, CDCl_3_): δ 169.8, 168.5, 167.3, 135.9, 134.3, 130.3, 128.4, 127.9, 127.3, 123.8, 119.5, 117.2, 65.5, 51.6, 38.5, 34.1, and 28.6; ES + HRMS calculated for C_28_H_28_BrN_3_O_3_SNa = 590.0912 and obtained = 590.0912.

##### 2.2.1.5 *N*-(*tert*-butyl)-2-(*N*-(4-fluorophenyl)-2-[3-oxo-3,4-dihydro-2*H*-benzo[*b*][1,4]-thiazin-2-yl)-acetamido]-2-phenylacetamide (8aD)

The reaction was carried out as mentioned for the synthesis of 8. The desired compound was obtained as a gray solid in 51% yield (115 mg), R_*f*_ = 0.3 (hexane:EtOAc, 7:3); ^1^H NMR (400 MHz, CDCl_3_): δ 8.67 (s, 1H), 7.27 (d, *J* = 1.4 Hz, 1H), 7.25 (d, *J* = 1.4 Hz, 1H), 7.24–7.13 (m, 4H), 7.13–7.07 (m, 3H), 6.98 (td, *J*_1_ = 7.6 Hz, *J*_2_ = 1.3 Hz, 2H), 6.82 (dd, *J*_1_ = 8.0 Hz, *J*_2_ = 1.1 Hz, 1H), 6.00 (s, 1H), 5.85 (s, 1H), 4.15 (dd, *J*_1_ = 8.0 Hz, *J*_2_ = 6.2 Hz, 1H), 2.75 (dd, *J*_1_ = 16.2 Hz, *J*_2_ = 6.2 Hz, 1H), 2.26 (dd, *J*_1_ = 16.2 Hz, *J*_2_ = 8.0 Hz, 1H), 1.33 (s, 9H); ^13^C NMR (100 MHz, CDCl_3_): δ 169.8, 168.5, 167.3, 163.2, 160.7, 135.9, 135.2, 135.1, 134.3, 130.3, 128.4, 127.9, 127.3, 123.8, 119.5, 117.2, 65.5, 51.7, 38.5, 34.1, and 28.6; ES + HRMS calculated for C_28_H_28_FN_3_O_3_SNa = 528.1733 and obtained = 528.1727.

##### 2.2.1.6 *N*-(*tert*-butyl)-2-(*N*-(3,5-dichlorophenyl)-2-(3-oxo-3,4-dihydro-2*H*-benzo[*b*][1,4]-thiazin-2-yl)-acetamido)-2-phenylacetamide (8aJ)

The reaction was carried out as mentioned for the synthesis of 8. The product was obtained as a white solid in 65% yield (162 mg), R_*f*_ = 0.3 (hexane:EtOAc, 7:3); ^1^H NMR (400 MHz, DMSO-d_6_): δ 10.57 (bs, 1H), 7.78 (s, 1H), 7.30 (d, *J* = 7.6 Hz, 2H), 7.25–7.13 (m, 4H), 7.05 (d, *J* = 8.4 Hz, 2H), 6.98 (t, *J* = 7.2 Hz, 2H), 6.87 (d, *J* = 7.6 Hz, 1H), 6.41 (bs, 1H), 6.03 (s, 1H), 3.82 (dd, *J*_1_ = 9.2 Hz, *J*_2_ = 4.4 Hz, 1H), 2.56–2.49 (m, 1H), 2.11–2.04 (m, 1H), 1.20 (s, 9H); ^13^C NMR (100 MHz, DMSO-d_6_): δ 168.38, 168.31, 165.9, 138.2, 136.6, 134.4, 132.5, 131.8, 131.5, 128.1, 127.9, 127.2, 123.2, 121.1, 117.8, 117.1, 63.0, 50.5, 38.0, 33.8, and 28.4; ES + HRMS calculated for C_28_H_27_Cl_2_N_3_O_3_SNa = 578.1048 and obtained = 578.1040.

##### 2.2.1.7 *N*-benzyl-*N*-(2-(*tert*-butylamino)-1-(4-nitrophenyl)-2-oxoethyl)-2-(3-oxo-3,4-dihydro-2*H*-benzo[*b*][1,4]-thiazin-2-yl)-acetamide (8bA)

The reaction was carried out as mentioned for the synthesis of 8. The desired compound was obtained as a pale yellow solid in 60% yield (147 mg), R_*f*_ = 0.3 (hexane:EtOAc, 7:3); ^1^H NMR (400 MHz, CDCl_3_): δ 8.23 (d, *J* = 8.4 Hz, 2H), 8.09 (d, *J* = 31.5 Hz, 2H), 7.69 (d, *J* = 8.4 Hz, 2H), 7.49 (d, *J* = 7.5 Hz, 1H), 7.36–7.30 (m, 2H), 7.19 (d, *J* = 7.0 Hz, 1H), 7.08 (dt, *J*_1_ = 13 Hz.4, *J*_2_ = 7.5 Hz, 3H), 6.35 (d, *J* = 26.5 Hz, 1H), 4.68 (d, *J* = 18.0 Hz, 2H), 4.33–4.18 (m, 1H), 3.17 (dd, *J*_1_ = 16.1 Hz, *J*_2_ = 5.3 Hz, 1H), 2.90 (dd, *J*_1_ = 16.1 Hz, *J*_2_ = 8.6 Hz, 1H), 1.49 (s, 9H); ^13^C NMR (100 MHz, CDCl_3_): δ 171.8, 168.7, 167.3, 147.6, 142.9, 137, 136.4, 130.5, 128.1, 127.8, 127.1, 126.8, 125.7, 123.7, 122.9, 120.1, 117.1, 113.2, 62.1, 51.3, 49.2, 38.7, 33.4, and 27.4; ES + HRMS calculated for C_29_H_30_N_4_O_5_SNa = 569.1829 and obtained = 569.1842.

##### 2.2.1.8 *N*-(*tert*-butyl)-2-[*N*-(3-chlorophenyl)-2-(3-oxo-3,4-dihydro-2*H*-benzo[*b*][1,4]-thiazin-2-yl)-acetamido]-2-(4-nitrophenyl)-acetamide (8bB)

The reaction was carried out as mentioned for the synthesis of 8. The desired compound was obtained as a greyish white solid in 55% yield (140 mg), R_*f*_ = 0.3 (hexane:EtOAc, 7:3); ^1^H NMR (400 MHz, CDCl_3_): δ 8.86 (bs, 1H), 8.02 (d, *J* = 7.2 Hz, 2H), 7.38 (d, *J* = 8.4 Hz, 2H), 7.29–7.27 (m, 2H), 7.21 (d, *J* = 6.8 Hz, 1H), 7.14–7.10 (m, 2H), 7.03–6.9 (m, 2H), 6.82 (d, *J* = 8 Hz, 1H), 6.19 (s, 1H), 5.99 (bs, 1H), 4.15–4.11 (m, 1H), 2.7 (q, *J* = 8 Hz, 1H), 2.32 (dd, *J*_1_ = 16 Hz, *J*_2_ = 6.4 Hz, 1H), 1.36 (s, 9H); ^13^C NMR (100 MHz, CDCl_3_): δ 170, 167.3, 147.6, 141.4, 140.1, 135.8, 131.1, 130.3, 129.2, 128.5, 128, 127.4, 124.1, 123.3, 119.7, 117.2, 64.8, 52, 38.4, 33.9, and 28.6; ES + HRMS calculated for C_28_H_27_ClN_4_O_5_S = 567.1361 and obtained = 567.1359.

##### 2.2.1.9 *N*-(4-bromophenyl)-*N*-(2-(*tert*-butylamino)-1-(4-nitrophenyl)-2-oxoethyl)-2-(3-oxo-3,4-dihydro-2*H*-benzo[*b*][1,4]-thiazin-2-yl)-acetamide (8bC)

The reaction was carried out as mentioned for the synthesis of 8. The product was obtained as a pale yellow solid in 58% yield (148 mg), R_*f*_ = 0.3 (hexane:EtOAc, 7:3); ^1^H NMR (400 MHz, CDCl_3_): δ 8.07–8.05 (m, 3H), 7.47–7.27 (m, 6H), 7.17 (t, *J* = 7.6 Hz, 1H), 7.01 (t, *J* = 8 Hz, 1H), 6.82 (d, *J* = 8 Hz, 1H), 6.32 (bs, 1H), 6.16 (s, 1H), 4.17 (d, *J* = 6 Hz, 2 Hz, 1H), 2.69 (dd, *J*_1_ = 8.4 Hz, *J*_2_ = 7.6 Hz, 1H), 2.28 (dd, *J*_1_ = 10 Hz, *J*_2_ = 6 Hz), and 1.39 (s, 9H); ^13^C NMR (100 MHz, CDCl_3_): δ 170.1, 167.3, 167.1, 147.6, 141.5, 137.9, 135.8, 132.6, 131.8, 131.5, 131.3, 127.9, 127.4, 124, 123.4, 119.8, 117.2, 64.4, 52, 38.5, 33.8, and 28.6; ES + HRMS calculated for C_28_H_27_BrN_4_O_5_SNa = 633.0783 and obtained = 633.0812.

##### 2.2.1.10 *N*-(*tert*-butyl)-2-(*N*-(4-fluorophenyl)-2-(3-oxo-3,4-dihydro-2*H*-benzo [*b*][1,4]-thiazin-2-yl)-acetamido)-2-(4-nitrophenyl)-acetamide (8bD)

The reaction was carried out as mentioned for the synthesis of 8. The desired compound was obtained as a pale white solid in 56% yield (138 mg), R_*f*_ = 0.3 (hexane:EtOAc, 7:3); ^1^H NMR (400 MHz, CDCl_3_): δ 8.28 (s, 1H), 8.04 (d, *J* = 8.86 Hz, 2H), 7.35 (d, *J* = 8.75 Hz, 2H), 7.28 (d, *J* = 1.26 Hz, 1H), 7.17–7.13 (m, 1H), 7.00 (td, *J*_1_ = 7.63 Hz, *J*_2_ = 1.24 Hz, 1H), 6.82 (dd, *J*_1_ = 7.95 Hz, *J*_2_ = 0.86 Hz, 1H), 6.35 (s, 1H), 6.16 (s, 1H), 4.16 (dd, *J* = 7.98 Hz, 6.37 Hz, 1H), 2.70 (q, *J* = 7.97 Hz, 1H), 2.28 (dd, *J*_1_ = 15.87 Hz, *J*_2_ = 6.32 Hz, 1H), and 1.38 (s, 9H); ^13^C NMR (100 MHz, CDCl_3_): δ 170.4, 167.4, 167.1, 147.6, 141.6, 135.8, 131.8, 131.7, 131.3, 127.9, 127.5, 124.0, 123.3, 117.2, 64.4, 52.0, 38.5, 33.8, and 28.6; ES + HRMS calculated for C_28_H_27_FN_4_O_5_SNa = 551.1686 and obtained = 551.1653.

##### 2.2.1.11 *N*-(2-(1*H*-indol-3-yl)-ethyl)-*N*-[2-(*tert*-butylamino)-1-(4-nitrophenyl)-2-oxoethyl]-2-(3-oxo-3,4-dihydro-2*H*-benzo[*b*][1,4]-thiazin-2-yl)-acetamide (8bE)

The reaction was carried out as mentioned for the synthesis of 8. The desired compound was obtained as a greyish white solid in 64% yield (172 mg), R_*f*_ = 0.15 (hexane:EtOAc, 7:3); ^1^H NMR (400 MHz, CDCl_3_): δ: 8.42–8 (m, 4H), 7.63–7.57 (m, 2H), 7.31–7.29 (m, 2H), 7.16–7.01 (m, 4H), 6.85–6.77 (m, 2H), 6.47 (bs, 1H), 5.89 (s, 1H), 4.24–4.19 (m, 1H), 3.86–3.50 (m, 2H), and 3.12–2.50 (m, 4H); ^13^C NMR (100 MHz, CDCl_3_): δ 170.9, 167.8, 167.2, 147.4, 143.1, 136.1, 135.9, 130.0, 129.6, 128.0, 127.5, 126.7, 125.0, 124.2, 123.6, 122.3, 122.1, 120.0, 119.7, 118.2, 117.2, 111.5, 111.3, 63.3, 51.9, 48.4, 38.6, 32.7, 31.9, and 28.6; ES + HRMS calculated for C_32_H_33_N_5_O_5_SNa = 622.2055 and obtained = 622.2056.

##### 2.2.1.12 *N*-(*tert*-butyl)-2-(4-nitrophenyl)-2-(2-(3-oxo-3,4-dihydro-2*H*-benzo[*b*][1,4]-thiazin-2-yl)-*N*-phenethylacetamido)-acetamide (8bF)

The reaction was carried out as mentioned for the synthesis of 8. The product was obtained as a gray solid in 60% yield (151 mg), R_*f*_ = 0.2 (hexane:EtOAc, 7:3); ^1^H NMR (400 MHz, CDCl_3_): δ 8.82 (bs, 1H), 8.25–8.14 (m, 3H), 7.62 (dd, *J*_1_ = 10.8 Hz, *J*_2_ = 8.8 Hz, 2H), 7.3 (t, *J* = 6.8 Hz, 1H), 7.19–7.14 (m, 5H), 7.03–6.99 (m, 1H), 6.89–6.84 (m, 3H), 6.52 (bs, 1H), 5.99 (s, 1H), 4.22 (t, *J* = 7 Hz, 1H), 3.70–3.47 (m, 2H), 3.13–3.06 (m, 1H), 2.91–2.31 (m, 3H), and 1.39 (s, 9H); ^13^C NMR (100 MHz, CDCl_3_): δ 170.8, 167.6, 167.3, 147.5, 143.0, 137.3, 137.1, 135.9, 129.9, 129.6, 128.7, 128.4, 128.0, 127.5, 127.1, 126.8, 124.2, 123.9, 123.7, 119.7, 117.3, 62.9, 51.9, 49.2, 38.7, 36.6, 32.5, and 28.5; ES + HRMS calculated for C_30_H_32_N_4_O_5_S = 561.2093 and obtained = 561.2061.

##### 2.2.1.13 *N*-(*tert*-butyl)-2-(*N*-(4-hydroxyphenyl)-2-(3-oxo-3,4-dihydro-2*H*-benzo[*b*][1,4]-thiazin-2-yl)-acetamido)-2-(4-nitrophenyl)-acetamide (8bI)

The reaction was carried out as mentioned for the synthesis of 8. The product was obtained as a pale brown solid in 55% yield (155 mg), R_*f*_ = 0.1 (hexane:EtOAc, 7:3); ^1^H NMR (400 MHz, CDCl_3_): δ 8.06–8.03 (m, 3H), 7.43–7.27 (m, 3H), 7.18–7.13 (m, 1H), 7.03–6.99 (m, 1H), 6.81–6.78 (m, 1H), 6.52–6.29 (m, 2H), 6.14–5.94 (m, 2H), 4.19–4.10 (m, 1H), 2.80–2.72 (m, 1H), 2.34–2.23 (m, 1H), and 1.40 (s, 9H); ^13^C NMR (100 MHz, CDCl_3_): δ 170.9, 168.0, 167.4, 156.6, 147.5, 141.7, 135.7, 131.2, 131.1, 131.0, 130.9, 130.8, 127.9, 127.4, 124.1, 123.2, 120.0, 117.3, 116.2, 65.3, 52.1, 38.4, 33.7, and 28.6; ES + HRMS calculated for C_28_H_28_N_4_O_6_SNa = 571.1627 and obtained = 571.1631.

##### 2.2.1.14 *N*-(4-bromophenyl)-*N*-(2-(*tert*-butylamino)-1-(4-chlorophenyl)-2-oxoethyl)-2-(3-oxo-3,4-dihydro-2*H*-benzo[*b*][1,4]-thiazin-2-yl)-acetamide (8cC)

The reaction was carried out as mentioned for the synthesis of 8. The product was obtained as a pale white solid in 52% yield (140 mg), R_*f*_ = 0.3 (hexane:EtOAc, 7:3); ^1^H NMR (400 MHz, CDCl_3_): δ 8.96 (d, *J* = 9.38 Hz, 1H), 7.28 (dd, *J*_1_ = 7.86 Hz, *J*_2_ = 1.34 Hz, 1H), 7.14–7.08 (m, 4H), 7.05 (d, *J* = 8.52 Hz, 2H), 6.99 (td, *J*_1_ = 7.58, *J*_2_ = 1.26 Hz, 1H), 6.82 (d, *J* = 7.78 Hz, 1H), 5.96 (d, *J* = 18.52 Hz, 2H), 4.09–4.18 (m, 1H), 2.68 (dd, *J*_1_ = 16.09 Hz, *J*_2_ = 7.46 Hz, 1H), 2.27 (dd, *J*_1_ = 16.07 Hz*, J*_2_ = 6.58 Hz, 1H), and 1.32 (s, 9H); ^13^C NMR (100 MHz, CDCl_3_): δ 170.0, 168.2, 167.4, 135.9, 134.4, 132.8, 132.1, 132.0, 132.0, 131.6, 128.5, 127.8, 127.3, 123.8, 119.3, 117.3, 64.5, 51.7, 38.5, 34.0, and 28.5; ES + HRMS calculated for C_28_H_27_BrClN_3_O_3_SNa = 622.0543 and obtained = 622.0554.

##### 2.2.1.15 *N*-(*tert*-butyl)-2-(4-chlorophenyl)-2-(*N*-(4-fluorophenyl)-2-(3-oxo-3,4-dihydro-2*H*-benzo[*b*][1,4]-thiazin-2-yl)-acetamido)-acetamide (8cD)

The desired compound was obtained as a pale white solid in 51% yield (121 mg), R_*f*_ = 0.2 (hexane:EtOAc, 7:3); ^1^H NMR (400 MHz, CDCl_3_): δ 8.06 (d, *J* = 24.53 Hz, 1H), 7.28 (d, *J* = 1.07 Hz, 1H), 7.19–7.13 (m, 3H), 7.07 (d, *J* = 8.48 Hz, 1H), 7.00 (td, *J*_1_ = 7.60 Hz, *J*_2_ = 1.20 Hz, 1H), 6.80 (dd, *J*_1_ = 7.95 Hz, *J*_2_ = 1 Hz, 1H), 5.99 (d, *J* = 4.28 Hz, 1H), 4.15 (t, *J* = 7.11 Hz, 1H), 2.72 (dd, *J*_1_ = 16.09 Hz, *J*_2_ = 7.22 Hz, 1H), 2.25 (dd, *J*_1_ = 16.05 Hz, *J*_2_ = 7.03 Hz, 1H), and 1.35 (s, 9H); ^13^C NMR (100 MHz, CDCl_3_): δ 169.8, 168.0, 167.0, 138.1, 135.8, 134.5, 132.8, 131.9, 131.7, 128.6, 128.0, 127.4, 123.9, 119.8, 117.1, 64.5, 51.8, 38.5, 33.9, and 28.6; ES + HRMS calculated for C_28_H_27_ClFN_3_O_3_SNa = 562.1343 and obtained = 562.1328.

##### 2.2.1.16 *N*-(2-(1*H*-indol-3-yl)-ethyl)-*N*-(2-(*tert*-butylamino)-1-(4-chlorophenyl)-2-oxoethyl)-2-(3-oxo-3,4-dihydro-2*H*-benzo[*b*][1,4]-thiazin-2-yl)-acetamide (8cE)

The reaction was carried out as mentioned for the synthesis of 8. The desired compound was obtained as an off-white solid in 62% yield (mg), R_*f*_ = 0.25 (hexane:EtOAc, 7:3); ^1^H NMR (400 MHz, CDCl_3_): δ 8.73 (bs, 1H), 7.44–7.29 (m, 5H), 7.19–7.14 (m, 4H), 7.02–6.91 (m, 1H), 6.90–6.76 (m, 1H), 6.21–5.94 (m, 2H), 4.25 (q, *J* = 8.8 Hz, 1H), 3.58–3.40 (m, 2H), 3.12–3.08 (m, 1H), 2.74–2.49 (m, 2H), 2.23–2.15 (m, 1H), and 1.36 (s, 9H); ^13^C NMR (100 MHz, CDCl_3_): δ 170.4, 168.3, 167.4, 137.7, 135.9, 134.4, 134.2, 133.9, 131.1, 130.8, 128.9, 128.6, 128.4, 128.1 127.5, 126.7, 126.6, 124.2, 119.7, 117.2, 61.9, 51.7, 48.3, 38.7, 36.3, 32.7, and 28.6; ES + HRMS calculated for C_32_H_33_ClN_4_O_3_SNa = 611.1854 and obtained = 611.1864.

##### 2.2.1.17 *N*-(*tert*-butyl)-2-(4-chlorophenyl)-2-(2-(3-oxo-3,4-dihydro-2*H*-benzo[*b*][1,4]-thiazin-2-yl)-N-phenethylacetamido)-acetamide (8cF)

The reaction was carried out as mentioned for the synthesis of 8. The product was obtained as a white solid in 55% yield (134 mg), R_*f*_ = 0.25 (hexane:EtOAc, 7:3); ^1^H NMR (400 MHz, CDCl_3_): δ 9.16 (bs, 1H), 8.25 (s, 1H), 7.41–7.28 (m, 5H), 7.13–7.11 (m, 3H), 7.04–6.94 (m, 2H), 6.90–6.87 (m, 1H), 6.63 (dd, *J*_1_ = 10 Hz, *J*_2_ = 2 Hz, 1H), 6.24 (bs, 1H), 5.93 (s, 1H), 4.23–4.19 (m, 1H), 3.62–3.47 (m, 2H), 3.08–2.56 (m, 3H), 2.46–2.40 (m, 1H), and 1.36 (s, 9H); ^13^C NMR (100 MHz, CDCl_3_): δ 170.5, 168.6, 167.5, 136.0, 134.4, 134.1, 133.9, 130.9, 128.9, 127.9, 127.4, 126.8, 124.0, 122.0, 119.5, 118.2, 117.3, 111.7, 111.2, 62.5, 51.7, 47.5, 38.6, 32.8, 28.6, and 25.8; ES + HRMS calculated for C_30_H_32_ClN_3_O_3_S = 550.1811 and obtained = 550.1816.

##### 2.2.1.18 *N*-(*tert*-butyl)-2-(4-chlorophenyl)-2-(*N*-(4-hydroxyphenyl)-2-(3-oxo-3,4-dihydro-2*H*-benzo[*b*][1,4]-thiazin-2-yl)-acetamido)-acetamide (8cI)

The reaction was carried out as mentioned for the synthesis of 8. The product was obtained as an off-white solid with 55% yield (108 mg), R_*f*_ = 0.1 (hexane:EtOAc, 7:3); ^1^H NMR (400 MHz, CDCl_3_): δ 9.07 (bs, 1H), 7.35–7.26 (m, 1H), 7.20 (d, *J* = 7.6 Hz, 1H), 7.12–7.04 (m, 5H), 6.95 (t, *J* = 7.4 Hz, 1H), 6.82–6.39 (m, 3H), 6.21 (bs, 1H), 5.87 (s, 1H), 4.14–4.07 (m, 1H), 2.80–2.71 (m, 1H), 2.36–2.27 (m, 1H), and 1.31 (s, 9H); ^13^C NMR (100 MHz, CDCl_3_): δ 170.8, 168.8, 167.6, 156.7, 135.7, 134.3, 132.9, 131.6, 130.8, 128.4, 127.8, 127.3, 123.9, 119.6, 117.4, 60.5, 51.9, 51.8, 38.4, and 28.5; ES + HRMS calculated for C_28_H_28_ClN_3_O_4_SNa = 560.1387 and obtained = 560.1390.

##### 2.2.1.19 *N*-(*tert*-butyl)-2-(furan-2-yl)-2-(*N*-(4-methoxyphenyl)-2-(3-oxo-3,4-dihydro-2*H*-benzo[*b*][1,4]-thiazin-2-yl)-acetamido)-acetamide (8dG)

The reaction was carried out as mentioned for the synthesis of 8. The desired compound was obtained as a pale brown solid in 48% yield (109 mg), R_*f*_ = 0.25 (hexane:EtOAc, 7:3); ^1^H NMR (400 MHz, CDCl_3_): δ 8.86 (bs, 1H), 7.28–7.25 (m, 3H), 7.12–7.10 (m, 1H), 7.00–6.98 (m, 1H), 6.83–6.81 (m, 1H), 6.72–6.56 (m, 2H), 6.30–6.25 (m, 2H), 6.18–6.15 (m, 2H), 4.16–4.11 (m, 1H), 3.73 (s, 3H), 2.78–2.71 (m, 1H), 2.35–2.27 (m, 1H), and 1.37 (s, 9H); ^13^C NMR (100 MHz, CDCl_3_): δ 170.3, 167.5, 166.5, 159.3, 147.8, 142.3, 135.9, 131.6, 127.9, 127.2, 123.7, 119.8, 117.2, 114.2, 112.1, 112.0, 110.6, 59.0, 55.3, 51.6, 38.5, 33.7, and 28.6; ES + HRMS calculated for C_27_H_29_N_3_O_5_S = 508.1861 and obtained = 508.1854.

##### 2.2.1.20 *N*-(*tert*-butyl)-2-(furan-2-yl)-2-(*N*-(4-nitrophenyl)-2-(3-oxo-3,4-dihydro-2*H*-benzo[*b*][1,4]-thiazin-2-yl)-acetamido)-acetamide (8dH)

The reaction was carried out as mentioned for the synthesis of 8. The desired compound was obtained as a reddish brown solid in 48% yield (112 mg), *R*_*f*_ = 0.3 (hexane:EtOAc, 7:3); ^1^H NMR (400 MHz, CDCl_3_): δ 8.06 (m, 3H), 7.86 (s, 1H), 7.30–7.26 (m, 2H), 7.17–7.13 (m, 1H), 7.03–6.98 (m, 1H), 6.77 (d, *J* = 8.00 Hz, 1H), 6.30–6.17 (m, 3H), 6.04 (s, 1H), 4.16 (t, *J* = 7.06 Hz, 1H), 2.71 (dd, *J*_1_ = 16.07 Hz, *J*_2_ = 7.09 Hz, 1H), 2.28 (dd, *J* = 16.04 Hz, 7.06 Hz, 1H), and 1.37 (s, 9H); ^13^C NMR (100 MHz, CDCl_3_): δ 169, 167.2, 166.1, 147.3, 146.9, 145.0, 143.2, 135.8, 131.2, 128.0, 127.3, 124.1, 124.0, 119.7, 117.2, 112.6, 110.8, 58.9, 51.9, 38.2, 33.8, 29.7, and 28.5; ES + HRMS calculated for C_26_H_26_N_4_O_6_SNa = 545.1471 and obtained = 545.1484.

##### 2.2.1.21 *N*-(4-bromophenyl)-*N*-(2-(t*ert*-butylamino)-2-oxo-1-(m-tolyl)-ethyl)-2-(3-oxo-3,4-dihydro-2*H*-benzo[*b*][1,4]-thiazin-2-yl)-acetamide (8eC)

The reaction was carried out as mentioned for the synthesis of 8. The product was obtained as a pale yellow solid in 52% yield (135 mg), R_*f*_ = 0.3 (30% EtOAc in hexane); ^1^H NMR (400 MHz, CDCl_3_): δ 8.42 (s, 1H), 7.43–7.27 (m, 1H), 7.13 (td, *J* = 7.7, 1.4 Hz, 1H), 7.06 (t, *J* = 7.5 Hz, 1H), 7.02–6.96 (m, 3H), 6.93 (s, 1H), 6.89 (d, *J* = 7.5 Hz, 2H), 6.80 (d, *J* = 7.9 Hz, 2H), 5.94 (s, 1H), 5.82 (s, 1H), 4.21–4.11 (m, 1H), 2.77 (dd, *J* = 16.2 Hz, 6.3 Hz, 1H), 2.26 (dd, *J* = 16.3 Hz, 7.9 Hz, 1H), 2.22 (s, 3H), and 1.34 (s, 9H); ^13^C NMR (100 MHz, CDCl_3_): δ 169.8, 168.6, 167.3, 138.0, 135.8, 135.3, 134.2, 132.1, 131.0, 129.1, 128.2, 127.9, 127.3, 123.8, 119.7, 117.1, 65.6, 51.6, 38.5, 34.0, 28.6, and 21.2; ES + HRMS calculated for C_29_H_30_BrN_3_O_3_S = 580.1225 and obtained = 580.1200.

##### 2.2.1.22 *N*-(*tert*-butyl)-2-(*N*-(4-fluorophenyl)-2-(3-oxo-3,4-dihydro-2*H*-benzo[*b*][1,4]-thiazin-2-yl)-acetamido)-2-(*m*-tolyl)-acetamide (8eD)

The reaction was carried out as mentioned for the synthesis of 8. The desired compound was obtained as a pale-brown solid in 55% yield (127 mg), R_*f*_ = 0.3 (hexane:EtOAc, 7:3); ^1^H NMR (400 MHz, CDCl_3_): δ 8.69 (s, 1H), 7.25 (d, *J* = 1.30 Hz, 1H), 7.13 (t, *J* = 7.68 Hz, 1H), 7.08–6.96 (m, 3H), 6.94 (s, 1H), 6.89 (d, *J* = 7.41 Hz, 1H), 6.82 (dd, *J*_1_ = 7.99 Hz, *J*_2_ = 1.11 Hz, 1H), 5.93 (s, 1H), 5.80 (s, 1H), 4.15 (dd, *J*_1_ = 7.91 Hz, *J*_2_ = 6.23 Hz, 1H), 2.77 (dd, *J*_1_ = 16.24 Hz, *J*_2_ = 6.23 Hz, 1H), 2.28–2.22 (m, 4H), and 1.32 (s, 9H); ^13^C NMR (100 MHz, CDCl_3_): δ 169.8, 168.7, 167.5, 163.2, 160.7, 138.0, 136.0, 135.2, 134.1, 132.4, 131.0, 129.1, 128.2, 127.9, 127.3, 123.8, 120.0, 117.1, 115.7, 115.5, 65.4, 51.6, 38.3, 28.6, and 21.2; ES + HRMS calculated for C_29_H_30_FN_3_O_3_SNa = 542.1890 and obtained = 542.1899.

##### 2.2.1.23 *N*-(2-(1*H*-indol-3-yl)-ethyl)-*N*-(2-(*tert*-butylamino)-1-(2-nitrophenyl)-2-oxoethyl)-2-(3-oxo-3,4-dihydro-2*H*-benzo[*b*][1,4-]-thiazin-2-yl)-acetamide (8fE)

The reaction was carried out as mentioned for the synthesis of 8. The desired compound was obtained as a pale brown solid in 59% yield (158 mg), R_*f*_ = 0.15 (hexane:EtOAc, 7:3); ^1^H NMR (400 MHz, CDCl_3_): δ 8.5–8.18 (m, 3H), 7.63 (dd, *J*_1_ = 8.4 Hz, *J*_2_ = 2.4 Hz, 2H), 7.33–7.3 (m, 1H), 7.22–7.17 (m, 4H), 7.02 (t, *J* = 7.6 Hz, 1H), 6.91–6.87 (m, 3H), 6.43 (bs, 1H), 5.95 (s, 1H), 4.26–4.21 (m, 1H), 3.71–3.48 (m, 2H), 3.14–3.07 (m, 2H), 2.93–2.38 (m, 3H), and 1.40 (s, 9H); ^13^C NMR (100 MHz, CDCl_3_): δ 170.8, 167.6, 167.3, 147.5,143.1, 137.2, 135.9, 130, 129.7, 128.7, 128.5, 128.1, 127.6, 126.9, 124.2, 123.7, 117.3, 63.0, 51.9, 49.2, 38.7, 36.1, 32.5, and 28.6; ES + HRMS calculated for C_32_H_33_N_5_O_5_SNa = 622.2094 and obtained = 622.2103.

##### 2.2.1.24 *N*-(2-(1*H*-indol-3-yl)-ethyl)-*N*-(2-(*tert*-butylamino)-1-(3-nitrophenyl)-2-oxoethyl)-2-(3-oxo-3,4-dihydro-2*H*-benzo[*b*][1,4]-thiazin-2-yl)-acetamide (8gE)

The reaction was carried out as mentioned for the synthesis of 8. The desired compound was obtained as a pale brown solid in 55% yield (148 mg), R_*f*_ = 0.15 (hexane:EtOAc, 7:3); ^1^H NMR (400 MHz, CDCl_3_): δ 8.20–8.05 (m, 3H), 7.60 (q, *J* = 7.2 Hz, 2H), 7.31–7.28 (m, 2H), 7.16–7.01 (m, 5H), 6.86–6.80 (m, 2H),6.43 (bs, 1H), 5.91 (s, 1H), 4.24–4.19 (m, 1H), 3.81–3.54 (m, 2H), 3.12–2.51 (m, 4H), and 1.39 (s, 9H); ^13^C NMR (100 MHz, CDCl_3_): δ 170.9, 167.9, 167.3, 148.1, 137.9, 137.7, 136.0, 135.8, 135.2, 134.9, 134.3, 129.4, 127.9, 127.4, 124.1, 123.8, 122.9, 122.1, 119.5, 119.3 118.0, 117.5, 111.3, 62.6, 53.4, 47.9, 38.7, 32.6, 30.8, and 28.5; ES + HRMS calculated for C_32_H_33_N_5_O_5_SNa = 622.2100 and obtained = 622.2110.

##### 2.2.1.25 *N*-benzyl-*N*-(2-(*tert*-butylamino)-2-oxo-1-(p-tolyl)-ethyl)-2-(3-oxo-3,4-dihydro-2*H*-benzo[*b*][1,4]-thiazin-2-yl)-acetamide (8hA)

The reaction was carried out as mentioned for the synthesis of 8. The desired compound was obtained as an off-white solid in 65% yield (150 mg), R_*f*_ = 0.3 (hexane:EtOAc, 7:3); ^1^H NMR (400 MHz, CDCl_3_): δ 9.25 (bs, 1H), 7.30–7.28 (m, 1H), 7.25–7.22 (m, 2H), 7.10–7.06 (m, 5H), 7.01–6.91 (m, 3H), 6.87–6.82 (m, 2H), 5.99–5.92 (m, 2H), 4.75–4.35 (m, 2H), 4.21–4.17 (m, 1H), 3.08–2.88 (m, 1H), 2.50–2.44 (m, 1H), 2.25 (s, 3H), and 1.31 (*s*, 9*H*); ^13^C NMR (100 MHz, CDCl_3_): δ 171.2, 168.9, 167.5, 138.2, 137.2, 135.9, 131.8, 129.7, 129.5, 129.2, 128.6, 128.3, 128, 128, 127.4, 127.2, 126.8, 126.1, 125.9, 123.8, 117.2, 117.1, 63.3, 51.6, 49.7, 38.6, 38.4, 33.5, 28.6, and 21; ES + HRMS calculated for C_30_H_33_N_3_O_3_SNa = 538.2135 and obtained = 538.2151.

##### 2.2.1.26 *N*-benzyl-*N*-(2-(*tert*-butylamino)-1-(4-methoxyphenyl)-2-oxoethyl)-2-(3-oxo-3,4-dihydro-2*H*-benzo[*b*][1,4]-thiazin-2-yl)-acetamide (8iA)

The reaction was carried out as mentioned for the synthesis of 8. The desired compound was obtained as a pale brown solid in 62% yield (147 mg), R_*f*_ = 0.3 (hexane:EtOAc, 7:3); ^1^H NMR (400 MHz, CDCl_3_): δ 9.23 (bs, 1H), 7.27–7.26 (m, 2H), 7.25–7.24 (m, 1H), 7.13–7.06 (m, 4H), 7.01–6.96 (m, 1H), 6.94–6.91 (m, 1H), 6.88–6.83 (m, 2H), 6.73–6.67 (m, 2H), 6.01–5.83 (m, 2H), 4.75–4.44 (m, 2H), 4.22–4.09 (m, 1H), 3.70 (s, 3H), 3.09–2.88 (m, 1H), 2.61–2.44 (m, 1H), and 1.32 (s, 9H); ^13^C NMR (100 MHz, CDCl_3_): δ 171.3, 171.1, 169, 169, 167.6, 159.5, 137.3, 137.2, 136.1, 135.9, 131.1, 131, 128.3, 127.9, 127.2, 127.2, 126.8, 126.7, 126.7, 126, 125.8, 123.8, 123.8, 119.9, 119.4, 117.3, 117.2, 113.9, 113.9, 99.9, 62.8, 62.4, 60.4, 55.2, 51.5, 49.5, 38.5, 38.3, 33.5, 33.4, and 28.6; ES + HRMS calculated for C_30_H_33_N_3_O_4_SNa = 554.2084 and obtained = 554.2092.

##### 2.2.1.27 *N*-(4-bromophenyl)-*N*-(2-(*tert*-butylamino)-1-(4-methoxyphenyl)-2-oxoethyl)-2-(3-oxo-3,4-dihydro-2*H*-benzo[*b*][1,4]-thiazin-2-yl)-acetamide (8iC)

The reaction was carried out as mentioned for the synthesis of 8. The desired compound was obtained as a pale yellow solid in 58% yield (155 mg), R_*f*_ = 0.3 (hexane:EtOAc, 7:3); ^1^H NMR (400 MHz, CDCl_3_): δ 8.52 (s, 1H), 7.25 (d, *J* = 1.4 Hz, 1H), 7.24 (d, *J* = 1.3 Hz, 1H), 7.12 (td, *J*_1_ = 7.7 Hz, *J*_2_ = 1.4 Hz, 1H), 7.05–6.93 (m, 4H), 6.82–6.76 (m, 1H), 6.71–6.67 (m, 2H), 5.94 (s, 1H), 5.78 (s, 1H), 5.28 (s, 2H), 4.13 (dd, *J*_1_ = 7.9 Hz, *J*_2_ = 6.3 Hz, 1H), 3.73 (s, 3H), 2.73 (dd, *J*_1_ = 16.2 Hz, *J*_2_ = 6.3 Hz, 1H), 2.22 (dd, *J*_1_ = 16.2 Hz, *J*_2_ = 7.9 Hz, 1H), and 1.31 (s, 9H); ^13^C NMR (100 MHz, CDCl_3_): δ 169.5, 168.7, 167.2, 159.5, 138.3, 135.8, 132.1, 131.6, 127.9, 127.3, 126.1, 123.8, 122.4, 119.5, 117.2, 113.8, 64.7, 55.2, 53.4, 51.6, 38.5, and 28.6; ES + HRMS calculated for C_29_H_30_BrN_3_O_4_SNa = 620.1018 and obtained = 620.1018.

##### 2.2.1.28 *N*-(*tert*-butyl)-2-(*N*-(4-fluorophenyl)-2-(3-oxo-3,4-dihydro-2*H*-benzo[*b*][1,4]-thiazin-2-yl)-acetamido)-2-(4-methoxyphenyl)-acetamide (8iD)

The reaction was carried out as mentioned for the synthesis of 8. The desired compound was obtained as a pale brown solid in 55% yield (132 mg), R_*f*_ = 0.3 (hexane:EtOAc, 7:3); ^1^H NMR (400 MHz, CDCl_3_): δ 8.21 (s, 1H), 7.29–7.27 (m, 1H), 7.16–7.11 (m, 1H), 7.05–6.94 (m, 5H), 6.79 (d, *J* = 8.0 Hz, 1H), 6.69 (d, *J* = 8.8 Hz, 3H), 5.97 (s, 1H), 5.82 (s, 1H), 4.17–4.13 (m, 1H), 3.74 (s, 3H), 2.75 (dd, *J*_1_ = 16.2 Hz, *J*_2_ = 6.3 Hz, 1H), 2.24 (dd, *J*_1_ = 16.2 Hz, *J*_2_ = 7.9 Hz, 1H), and 1.33 (s, 9H); ^13^C NMR (100 MHz, CDCl_3_): δ 169.7, 168.8, 167.2, 160.7, 159.4, 135.8, 135.2, 132.2, 131.6, 12, 127.3, 126.3, 123.8, 119.6, 117.1, 113.7, 64.7, 55.2, 51.6, 38.5, 34, and 28.6; ES + HRMS calculated for C_29_H_30_FN_3_O_4_SNa = 558.1839 and obtained = 558.1840.

##### 2.2.1.29 *N*-(*tert*-butyl)-2-(2-chlorophenyl)-2-(*N*-(3-chlorophenyl)-2-(3-oxo-3,4-dihydro-2*H*-benzo[*b*][1,4]-thiazin-2-yl)-acetamido)-acetamide (8jB)

The reaction was carried out as mentioned for the synthesis of 8. The desired compound was obtained as a white solid in 45% yield (112 mg), R_*f*_ = 0.3 (hexane:EtOAc, 7:3); ^1^H NMR (400 MHz, CDCl_3_): δ 9.12 (bs, 1H), 7.76–7.27 (m, 2H), 7.13–6.94 (m, 8H), 6.86–6.82 (m, 2H), 6.32 (s, 1H), 5.94 (bs, 1H), 4.17–4.12 (m, 1H), 2.81–2.70 (m, 1H), and 2.36–2.25 (m, 1H); ^13^C NMR (100 MHz, CDCl_3_): δ 169.5, 168.1, 167.6, 140.0, 135.9, 135.3, 131.4, 129.9, 129.4, 128.6, 127.9, 127.2, 126.7, 123.8, 119.8, 119.2, 117.3, 62.1, 51.9, 51.8, 38.4, and 28.7; ES + HRMS calculated for C_28_H_27_Cl_2_N_3_O_3_SNa = 578.1048 and obtained = 578.1039.

##### 2.2.1.30 *N*-(*tert*-butyl)-2-(*N*-(4-methoxyphenyl)-2-(3-oxo-3,4-dihydro-2*H*-benzo [*b*][1,4]-thiazin-2-yl)-acetamido)-butanamide (8kG)

The reaction was carried out as mentioned for the synthesis of 8. The desired compound was obtained as a pale brown solid in 52% yield (109 mg), R_*f*_ = 0.35 (hexane:EtOAc, 7:3); ^1^H NMR (400 MHz, CDCl_3_): δ 8.34 (bs, 1H), 7.30–7.28 (m, 1H), 7.21–7.14 (m, 2H), 7.06–6.99 (m, 1H), 6.90–6.79 (m, 4H), 6.55 (bs, 1H), 4.90–4.87 (m, 1H), 4.14–4.10 (m, 1H), 3.78 (s, 3H), 2.63 (dd, *J*_*1*_ = 16.0 Hz, *J*_*2*_ = 7.2 Hz, 1H), 2.33 (dd, *J*_*1*_ = 16.0 Hz, *J*_*2*_ = 7.2 Hz, 1H), 1.84–1.6 (m, 2H), 1.37 (s, 9H), and 0.89 (t, *J* = 7.2 Hz, 3H); ^13^C NMR (100 MHz, CDCl_3_): δ 170.8, 169.6, 167.2, 159.4, 135.9, 130.7, 127.9, 127.3, 123.8, 119.8, 117.1, 114.5, 60.8, 55.4, 51.2, 38.4, 33.8, 28.6, 21.5, and 10.8; ES + HRMS calculated for C_25_H_31_N_3_O_4_SNa = 492.1933 and obtained = 492.1938.

### 2.3 Computational studies

#### 2.3.1 Molecular modeling studies

In this study, we designed and synthesized novel 1,4-benzothiazine-based bisamides that might act as peptide deformylase inhibitors. After the pre-treatment of the peptide deformylase enzyme (PDB ID: 1Q1Y), the structure was corrected and completed. The natural product actinonin was used as the reference standard and was fully displayed. According to the docking score and drug-likeness, 28 candidate molecules were selected, and all of them could bind to the active site.

#### 2.3.2 Protein preparation

The X-ray crystallographic structure of the PDF protein was procured from the Research Collaboratory of Structural Bioinformatics (RCSB) Protein Data Bank (http://www.rcsb.org) (PDB ID: 1Q1Y), having a resolution of 1.90 Å (Yoon et al., 2004). MGLTools 1.5.6 (Molecular Graphics Laboratory, The Scripps Research Institute, La Jolla, United States) was used to model the protein. Energy minimization was performed using Swiss-PdbViewer (SPDBV 4.1.0, Swiss Institute of Bioinformatics). To remove the interference from water molecules, the protein molecule was desolvated. To free up the binding cavity, extra chains were cut out, and the co-crystal ligand was removed. After attaching polar hydrogens and assigning bond order, Gasteiger partial atomic charges were applied. The protein PDB format was changed to PDBQT by adding charges (Q) and switching the compatibility to AutoDock4 (T) type.

#### 2.3.3 Ligand preparation

All the ligands were sketched using ChemDraw 15.0, subjected to energy minimization using the MM2 minimization protocol, and saved in PDB format. MGLTools version 1.5.6 was used for ligand modeling. The ligands were subjected to the software program, and the torsional degrees of freedom (torsdof) were added to each molecule. The Gasteiger–Marsili approach was then used to add partial atomic charges (Q) and make AutoDock4 (T) compatible, transforming each ligand to the PDBQT format.

#### 2.3.4 Molecular docking and analysis

Molecular docking studies were performed using AutoDock Vina (Trott and Olson, 2010). The active site domain was encircled by a grid box with a spacing of 1 Å and 24 × 24 × 24 dimensions in all directions for x, y, and z planes with the resolution set to −18.189, 142.096, and 39.78 at the x, y, and z centers, respectively. The compounds were compared to the standard actinonin molecule based on the binding energy (kcal/mol), and the highest actives were determined. Using PyMOL, the docked complex was retrieved and saved in PDB format. In order to analyze the docked complexes, BIOVIA Discovery Studio Visualizer version 20 was used. Prior to docking, the docking studies were validated using AutoDock Vina. For this, the co-crystal ligand and actinonin were removed and then redocked again within the active site pocket of the PDF receptor. The root mean square deviation (RMSD) value between the redocked conformer and the initial X-ray crystallographic conformation of the co-crystal was found to be 0.048. Validation using AutoDock Vina showed no appreciable differences. The least binding energy and the ligand–receptor interactions were considered for the docking analysis.

#### 2.3.5 Predicted pharmacokinetic properties

The pharmacokinetic properties of the synthesized benzothiazine-3-one derivatives were analyzed using SwissADME (http://www.swissadme.ch/) and toxicity prediction utilizing the ProTox-II server (https://tox-new.charite.de/protox_II/). The program calculated the results automatically after inserting the smiles of the compounds.

#### 2.3.6 Molecular dynamics simulation

To determine the stability of the docked complex and the ligand–protein time of contacts and residence interaction percentage, the molecular dynamics (MD) simulation was performed for compound **8bE** bound with the PDF receptor using the GROningen Machine for Chemical Simulations (GROMACS) (Van Der Spoel et al., 2005) 2021.2 package. Ligand and protein topologies were created using Discovery Studio software and submitted for final simulation using CHARMM36 force field parameters. The energy minimization parameters were set for 5,000 steps using the steepest descent method. The MD simulation was conducted for 100 ns with constant temperature “T” (300 K), volume “V,” number of atoms “N” (NVT), and pressure “P” (1.0 bar) (NPT) GROMACS equilibration parameters. The leap-frog MD integrator was used, with an estimated frame rate of 1,000 per simulation. MD trajectories were analyzed for the protein and ligand: root mean square deviation **RMSD**, root mean square fluctuation (**RMSF**), radius of gyration (**Rg**), protein–ligand hydrogen bonding (**H-bonds**), and solvent-accessible surface area (**SASA**) during a 100-ns timeframe.

### 2.4 Biological assay

#### 2.4.1 Protocols for microbiological assay

The protocols used here are a step-by-step adjustment and follow the guidelines described by the Clinical and Laboratory Standards Institute (CLSI), United States.

Media: The culture media were prepared using Luria-Bertani (LB) broth (Miller) without any supplements at a concentration of 25 g/L.

Bacteria: The assays were performed on MTCC 3160 *S. aureus*, which was cultured in LB broth 24 h before the experiment from a previously prepared bacterial colony plate. It was normalized to 0.08–0.13 OD_600_ as a density equivalent to 10^8^ CFU/mL. This was used for inoculation.

Preparation of test molecules: All molecules were dissolved in DMSO to obtain a stock solution of 5 mg/mL. Before the experiment, aliquots of different concentrations were prepared by diluting the required quantity with dd water and then filtered via a 0.22-µm filter. To avoid any effect of DMSO, its concentration was kept below 1% at each well.

#### 2.4.2 Bacterial cell viability assay

Experimental procedure: All the experiments were done inside laminar air flow using pre-sterilized equipment like micropipettes, PBS solution, and LB medium microtiter plates. Here, 100 µL of 1 mg/mL drug solution was added to 100 µL of LB broth in the first column of a 48-well plate, and two-fold serial dilution was performed thereafter. Then, 100 µL of bacterial suspension (10^6^ CFU/mL) was added to each well to obtain a final concentration of 5 × 10^5^ CFU/mL. The well without drug was used as the positive control, the well with the media alone was used as the blank, and 10 μg/mL amoxicillin was used as the negative control. The plate was incubated at 37°C for 16 h. The optical density of the bacterial cultures was measured at 600 nm using a Multiskan SkyHigh Microplate Spectrophotometer (Thermo Fisher Scientific). Data are presented as the mean standard deviation with n = 6 (Wiegand et al., 2008 **)**.

#### 2.4.3 MBIC determination by crystal violet assay

Experimental procedure: All the experiments were done inside laminar air flow using pre-sterilized equipment like micropipettes, PBS solution, and LB medium microtiter plates. In a pre-sterilized microtiter plate, 75 µL of pre-sterilized Luria-Bertani broth (Miller) was added. To this, 20 µL of previously prepared aliquots of the drug/molecule was added with vigorous pipetting to ensure complete solubilization. To this, 5 µL of bacterial culture (OD = 0.01) was added, and the plate was incubated at 37°C for 16–18 h. The culture media were carefully pipetted and washed with 1× PBS (100 µL *3). Then, 100 µL solution of 4% paraformaldehyde was added to dd water and incubated for 30 min. Then, the solution was carefully removed and washed with 1× PBS (100 µL *3). Then, 105 µL of 0.1% crystal violet solution was added, incubated for 30 min, and then washed with 1× PBS (100 µL *3). It was left to dry for 30 min, so no traces of the crystal violet solution were left. In most cases, a fine layer of a violet-colored film will be visible.

Then, 105 µL ethanol was added and incubated for 10 min (a 33% solution of acetic acid in dd water can also be used, but it requires incubation of 30 min). Absorbance was read at 570 nm (O'Toole, 2011).

#### 2.4.4 Catheter-associated biofilm formation

Experimental procedure: A natural rubber latex silicone catheter was used for this assay. The circular disks (0.2 cm height and 0.25 cm diameter) of the catheter were excised and kept in long-range (350 nm) UV radiation 12 h before the experiment. These were incubated in a 48-well microtiter plate along with the cultured *S. aureus* (0.01 OD_600_) and test molecule for 24 h at 37°C under static conditions. After 24 h, crystal violet assay was used to quantify biofilm formation (Colomer-Winter et al., 2019).

## 3 Results and discussions

### 3.1 Design of molecules

**FIGURE 3:**
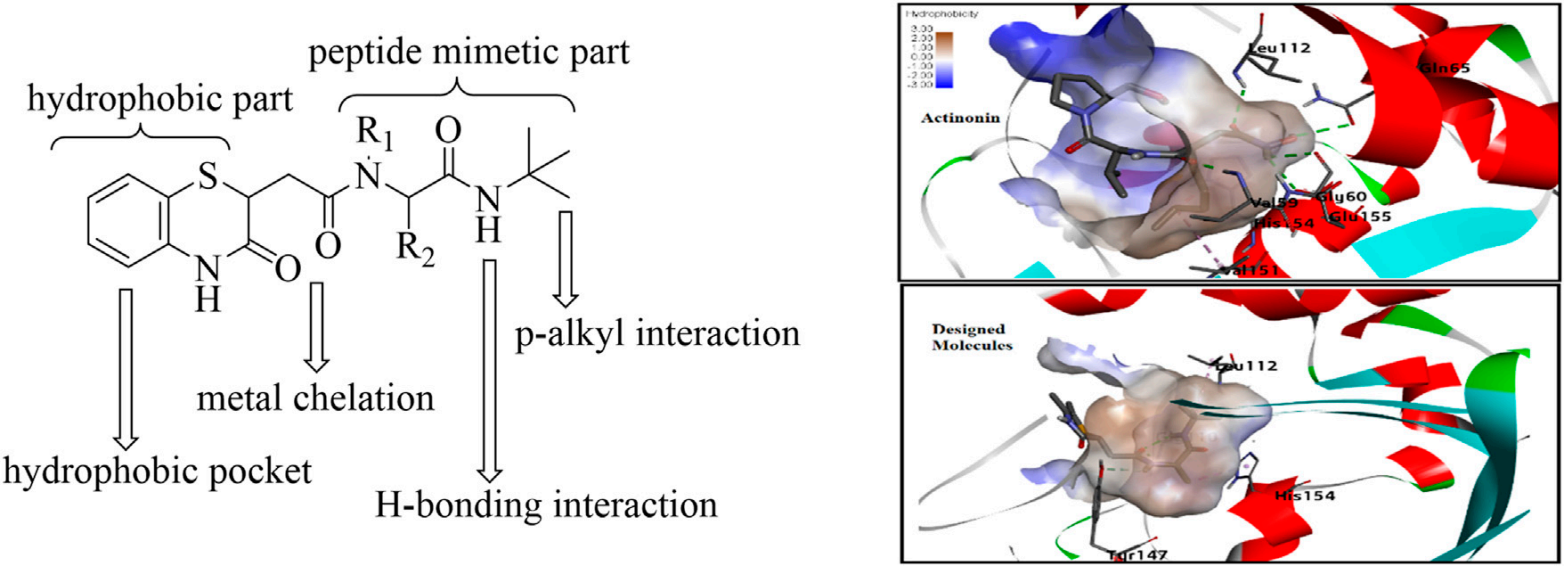
Design of the prototype structure as plausible PDF inhibitors.

As supported by the molecular modeling data, the bisamide side chain could act as the therapeutically significant peptidomimetics part. The compound with prototype structure I was docked with the crystal structures of PDF from *S. aureus*, and the binding of the molecule was compared with the standard actinonin (Figure 3) (Yoon et al., 2004). The computational data showed that the molecules could fit well within the binding pocket of the PDF receptor, and like the standard actinonin, the benzothiazine molecules would bind to the hydrophobic pocket of the receptor, in which the peptidomimetic part would be more adhered toward the pocket. The *tert*-butyl group could impart pi–alkyl interactions, and the –NH part of the amine could form a stable hydrogen bond with amino acid residues. In molecular modeling, another significant feature observed was the interaction with the Gly110 residues, and a literature report indicates that this interaction is essential for peptide deformylase (Kumar et al., 2014). Therefore, modifications only at R_1_ and R_2_ of the peptidomimetic part are planned with the hypothesis that it may provide better van der Waals interaction.

### 3.2 Synthesis

**SCHEME 1:**
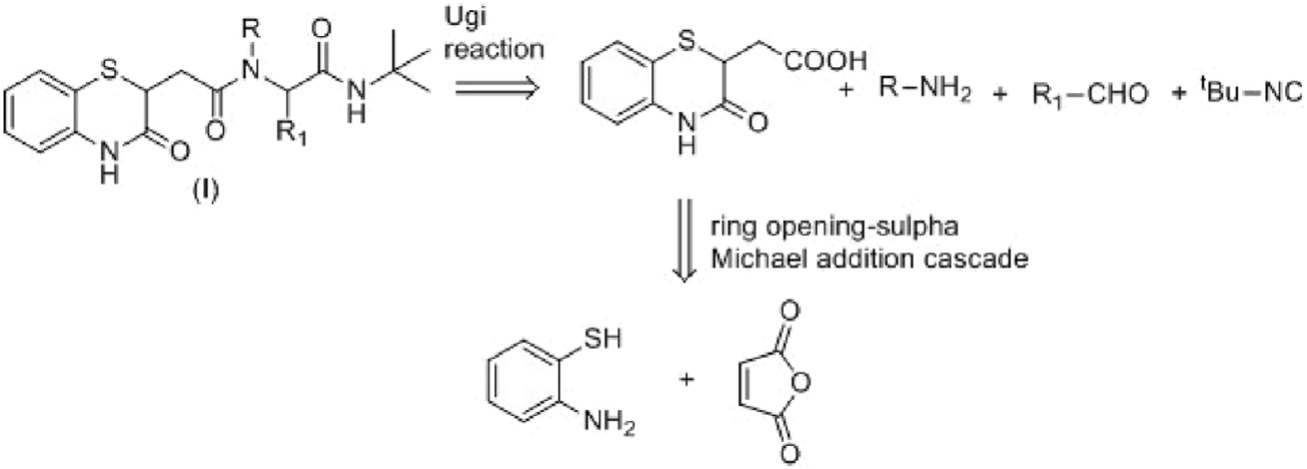
Retrosynthetic plan for the synthesis of the designed molecules.

Maleic anhydride is a fascinating cyclic anhydride with vast applications in polymer science, biotechnology, or API synthesis (Kamada et al., 2003). In the presence of amines, maleic anhydride undergoes ring opening, followed by dehydrative cyclization to form *N*-substituted maleimides (Scheme 1) (Kaur et al., 2013). We recently demonstrated that the cyclization path of maleic anhydride can be controlled to produce benzoxazole derivatives (Kumar et al., 2023). Herein, we envisage that maleic anhydride in the presence of 2-aminothiophenol would undergo cascade anhydride ring opening, followed by intramolecular sulfa-Michael addition to produce the desired 1,4-benzothiazine-3-carboxylic acid derivative in one pot (Scheme 1), which could further be employed in the Ugi four-component reaction of 3 with a suitable amine, aldehyde, and isocyanide to achieve the designed molecule **8**. The Ugi four-component reaction is one of the useful multicomponent reactions for synthesizing diverse heterocyclic scaffolds, natural products, and biologically active molecules (Fouad et al., 2020).

**SCHEME 2:**
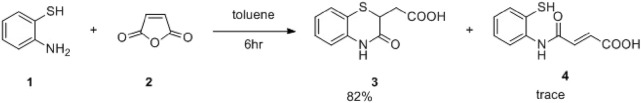
One-pot synthesis of 1,4-benzothiazine-3-carboxylic acid.

**SCHEME 3:**
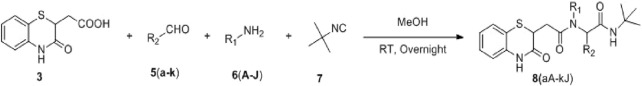
Synthesis of benzothiazine-3-one containing bisamide derivatives.

With that hypothesis, the reaction was carried out, and 1,4-benzothiazine-3-carboxylic acid was obtained in 82% yield along with 15% of the non-cyclized product. Then, to achieve better conversion, 1 equivalent of Et_3_N was used, and within 3 h, the yield of the desired product improved significantly to 96% (Scheme 2), and no non-cyclized product was obtained. Then, as hypothesized, the Ugi reaction of 3 with *tert*-butyl isocyanide, benzaldehyde, and benzyl amine in MeOH was carried out at room temperature. As aimed, within 14 h, the reaction was completed, and isolation of the compound afforded compound **8aA** in 60% yield (Scheme 3). Then, the reaction scope was explored by using different aldehydes and amines containing electron-donating and electron-withdrawing groups at the different positions of the aromatic ring (Table 1). The isocyanides were not changed as in molecular docking, the *tert*-butyl group exerts three pi–alkyl interactions, which is responsible for good docking scores. Aliphatic amines like tryptamine or benzyl amines were used to check the effect of the carbon chain on the activities.

**TABLE 1:** Docking scores, yield, and antibacterial activities of the synthesized compounds.

Entry	Compound	Docking score	Yield (%)	Antibacterial activity MIC (μg/mL)			
				Sa	Bc	Ec	Vc
1	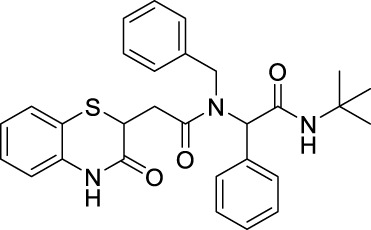 **8aC**	−7.8	60	55–60	>100	>100	>100
2	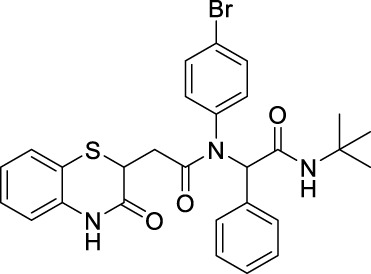 **8aC**	−8.0	54	55–60	>100	>100	>100
3	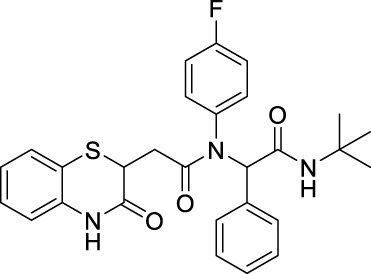 **8aD**	−8.2	51	55–60	>100	>100	>100
4	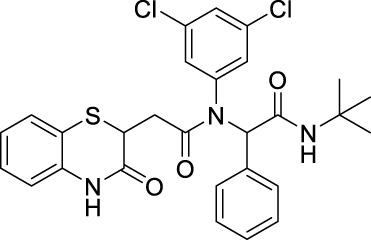 **8aJ**	−8.6	65	55–60	>100	>100	>100
5	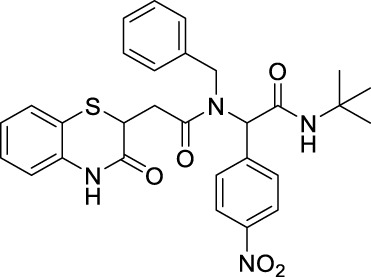 **8bA**	−8.7	60	50.48 ± 1.28	>100	>100	>100
6	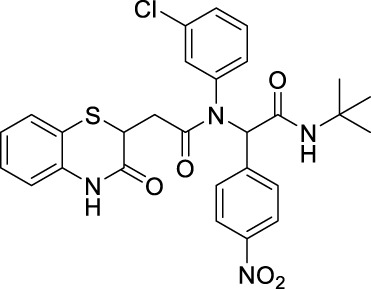 **8bB**	−9.1	55	30.48 ± 1.55	>100	>100	>100
7	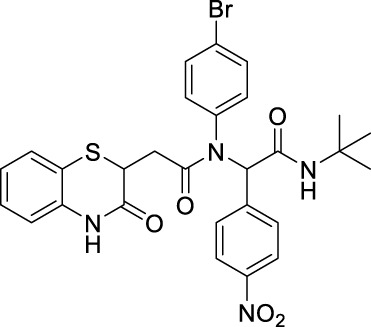 **8bC**	−8.5	58	47.5 ± 2.5	>100	>100	>100
8	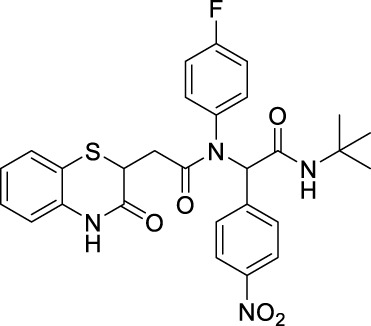 **8bD**	−8.6	56	46.25 ± 2.25	>100	>100	>100
9	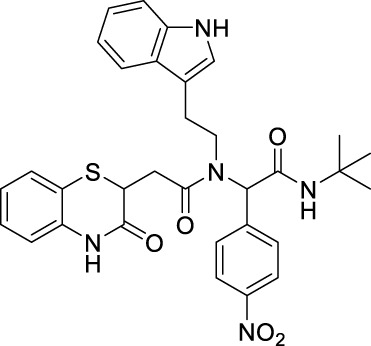 **8bE**	−8.3	64	6.16 ± 2.17	>100	>100	>100
10	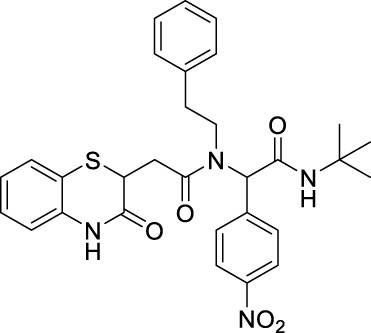 **8bF**	−7.0	60	34.15 ± 2.42	>100	>100	>100
11	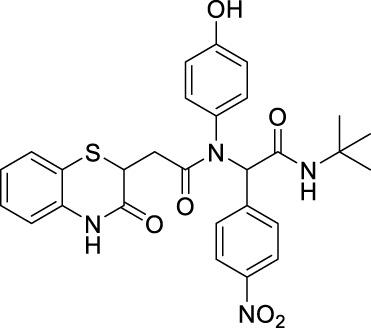 **8bI**	−8.4	51	42.62 ± 3.45	>100	>100	>100
12	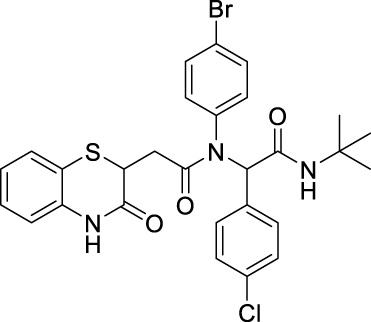 **8cC**	−7.9	52	15.56 ± 1.44	>100	>100	>100
13	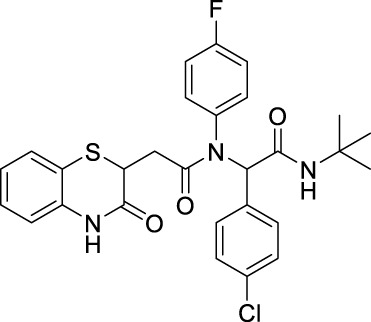 **8cD**	−8.4	51	25.55 ± 2.45	>100	>100	>100
14	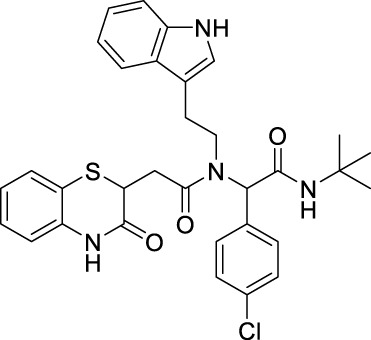 **8cE**	−9.0	52	12.39 ± 1.64	>100	>100	>100
15	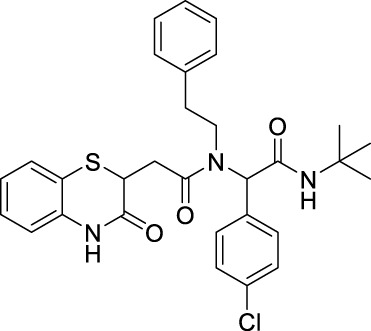 **8cF**	−8.1	55	38.47 ± 1.14	>100	>100	>100
16	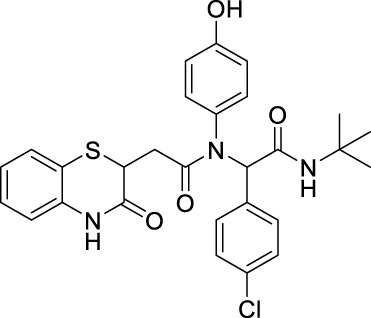 **8cI**	−7.8	55	48.47 ± 2.13	>100	>100	>100
17	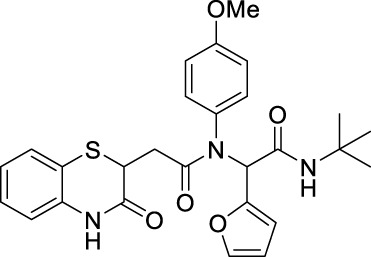 **8dG**	−7.7	48	28.36 ± 3.24	>100	>100	>100
18	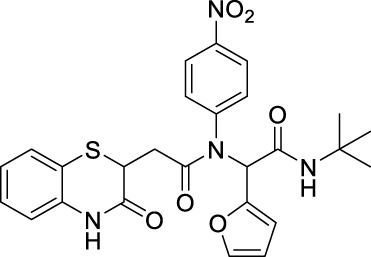 **8dH**	−8.0	48	18.47 ± 1.28	>100	>100	>100
19	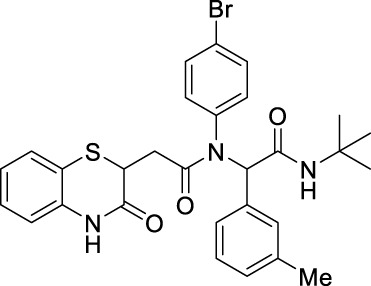 **8eC**	−8.3	52	22.45 ± 0.85	>100	>100	>100
20	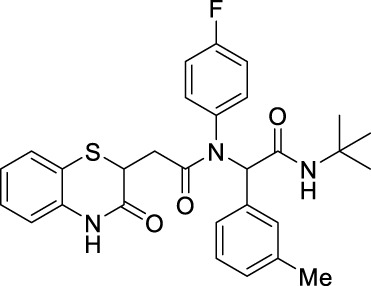 **8eD**	−7.5	55	47.62 ± 1.75	>100	>100	>100
21	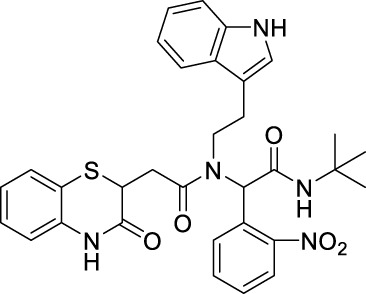 **8fE**	−7.5	59	>100	>100	>100	>100
22	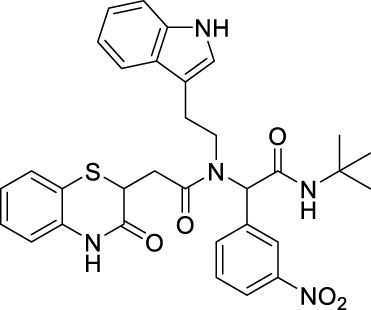 **8gE**	−8.4	55	>100	>100	>100	>100
23	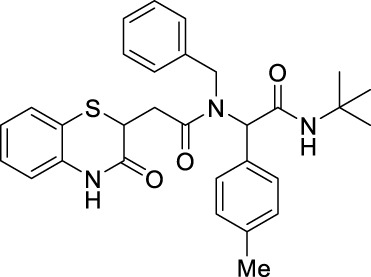 **8hA**	−8.1	65	55–60	>100	>100	>100
24	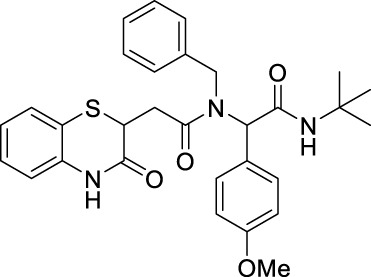 **8iA**	−7.7	62	55–60	>100	>100	>100
25	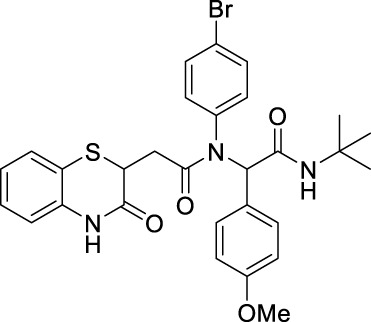 **8iC**	−7.3	58	55–60	>100	>100	>100
26	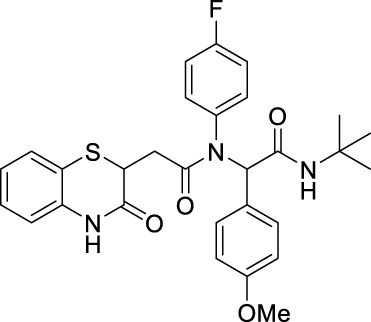 **8iD**	−7.4	55	55–60	>100	>100	>100
27	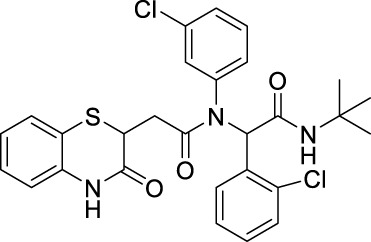 **8jB**	−7.4	45	>100	>100	>100	>100
28	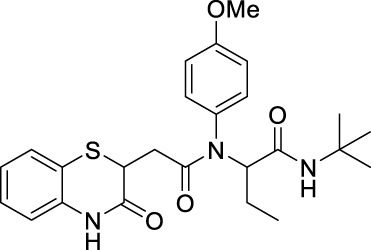 **8kG**	−7.3	52	>100	>100	>100	>100
29 (Margolis et al., 2000; Clements et al., 2001; Jung et al., 2016)	Actinonin	−7.1		16	0.192	64	-
30 (Scazzocchio et al., 2006; Coutinho et al., 2010; Bier et al., 2015)	Gentamicin	−7.3		12.5	4	4	2

### 3.3 Antibacterial screening

After the structural characterization by HRMS and NMR spectra, all the newly synthesized molecules were evaluated for *in vitro* antibacterial activity (Table 1) against both Gram-positive and Gram-negative bacteria, including *S. aureus* (MTCC 3160), *Bacillus cereus* (MTCC 1272), *Escherichia coli* (MTCC 1667), and *Vibrio cholerae*. Among the compounds screened, nine compounds exhibited very good antibacterial activities against *S. aureus*, of which compound **8bE** showed better activity than the references gentamicin and actinonin. In these compounds, it was observed that the substitution of aromatic rings at the *para* position leads to maximal efficacy. Of all the compounds tested, **8bE** showed the highest activity with an MIC_50_ value of 6.16 ± 2.17 *μ*g/mL (Table 1, **entry 9)**. It contained tryptamine as amine and 4-nitrobenzaldehyde as the aldehyde part. Furthermore, in most cases, alkyl-substituted anilines showed better inhibition except in the cases of 4-chlorobenzaldehyde (Table 1, **entry 12**). So, it can be assumed that alkyl amines are a better substrate in this case, along with the *para*-substituted benzaldehyde substrate. It is crucial to note that heteroaromatic rings, such as furfuryl, are also active **(** Table 1 **, entry 17** & **18)**, while aliphatic aldehydes do not show activity **(** Table 1, **entry 28)**. Another important feature is that other substitutions at *ortho* or *meta* in the benzaldehyde ring do not show potent activity, with only the *m*-methyl analog showing potential activity **(** Table 1, **entries 19** and **20)**.

### 3.4 Biofilm inhibition screening

After checking the MIC values, the compounds were further screened against *S. aureus* biofilms. It was found that compounds **8bE** and **8cE**, which were the most potent molecules against *S. aureus*, also demonstrated excellent biofilm inhibitory activities (Table 2). This result indicated that compounds **8bE** and **8cE** can display dual inhibitory properties.

**TABLE 2:** Biofilm inhibitory activities of the synthesized compounds.

Entry	Compound	MBIC (µg/mL)	Entry	Compound	MBIC (µg/mL)	Entry	Compound	MBIC (µg/mL)
1	**8aA**	80	11	**8bI**	80	21	**8fE**	>100
2	**8aC**	80	12	**8cC**	34.26 ± 1.74	22	**8gE**	>100
3	**8aD**	80	13	**8cD**	80	23	**8hA**	80
4	**8aJ**	80	14	**8cE**	14.31 ± 2.44	24	**8iA**	80
5	**8bA**	80	15	**8cF**	42.16 ± 1.86	25	**8iB**	80
6	**8bB**	80	16	**8cI**	80	26	**8iD**	80
7	**8bC**	50–55	17	**8dG**	36.49 ± 1.87	27	**8jB**	>100
8	**8bD**	50–55	18	**8dH**	24.32 ± 2.54	28	**8kG**	>100
9	**8bE**	**10.42 ± 2.18**	19	**8eC**	45 ± 1.07	29 (Al-Musawi et al., 2020)	Trans-chalcone	20
10	**8bF**	45.36 ± 3.24	20	**8eD**	80	30 (Park et al., 2022)	Kanamycin	50

The bold value highlights the highest MBIC values obtained by prepared derivatives.

#### 3.4.1 Validation of biofilm inhibitory properties

**FIGURE 4:**
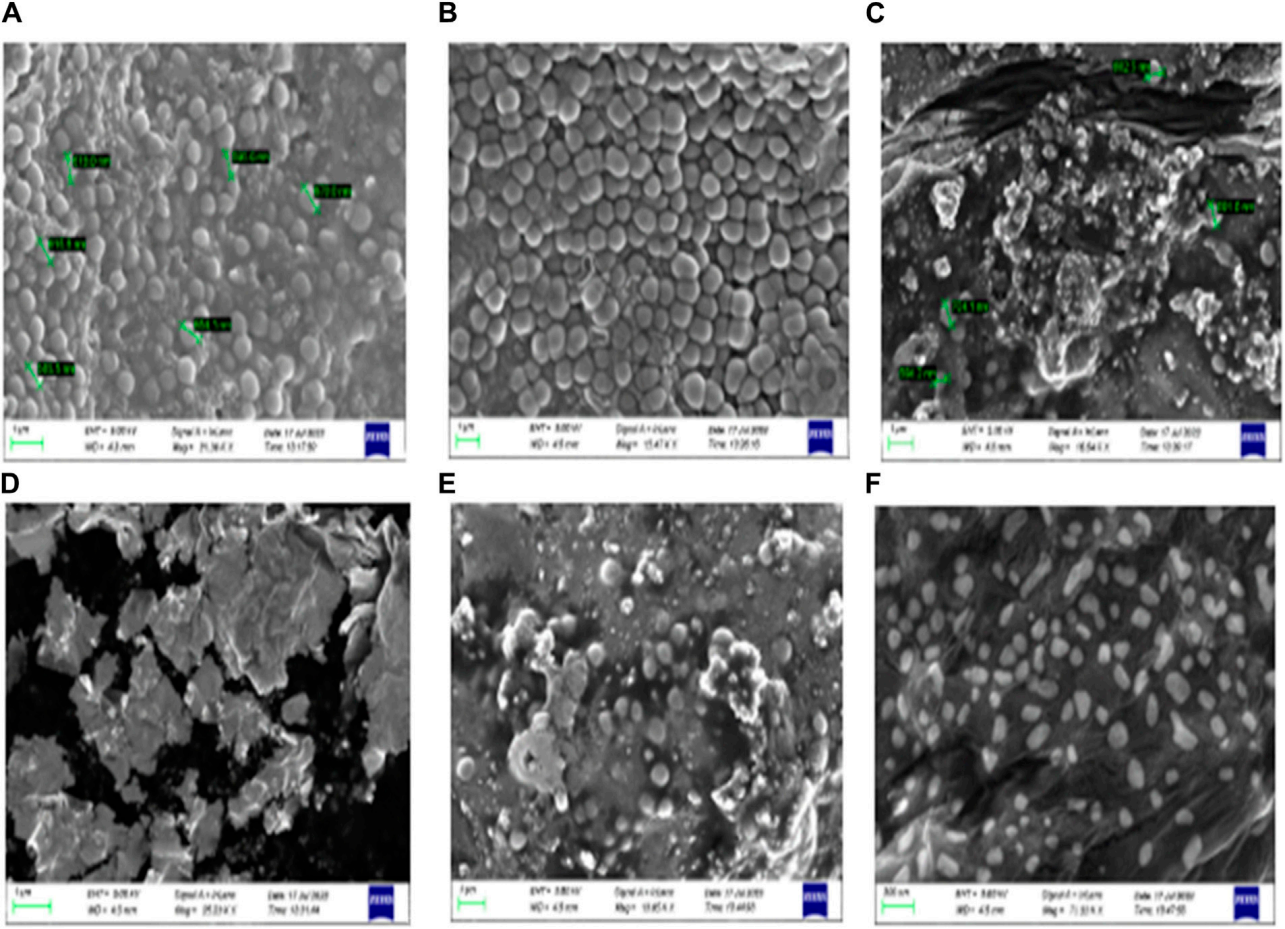
**(A)** Untreated *Staphylococcus aureus* biofilm. **(B)** Treated with a carrier or blank solvent. **(C)** After incubation with 6 μg/mL 8bE. **(D)** After incubation with 12 μg/mL 8bE. **(E)** After incubation with 12 μg/mL 8bE at 1,000× magnification. **(F)** After incubation with 12 μg/mL 8bE at 7,000× magnification.

Furthermore, a field emission scanning electron microscope (SEM) was used to validate the anti-biofilm effect of **8bE**. A *S. aureus* biofilm developed on the surface of glass slides in a multi-well plate (Kong et al., 2018), and to this plate, compound **8bE** was incubated at concentrations of 6 μg/mL and 12 μg/mL. The morphology of the untreated *S. aureus* biofilm is shown in Figures 4A,B. On incubation with 6 μg/mL of 8bE, disruption of the biofilm was visible (Figure 4C). On increasing the concentration to 12 μg/mL, patches of the remaining biofilm were visible (Figure 4D). On 1,000× magnification, the biofilm appeared as a monolayer with cells scattered throughout the layer (Figure 4E). On further magnification of 7,000×, bacterial cells appeared as finely dispersed with no visible sign of clumping (Figure 4F). The SEM pictures supported the ability of the molecule to inhibit biofilm formation.

#### 3.4.2 Biofilm inhibitory assay on the urinary catheter

**FIGURE 5:**
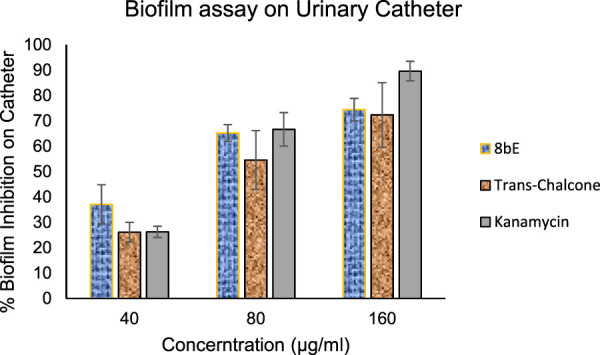
Biofilm inhibition on the urinary catheter.

After confirming the biofilm inhibitory properties of 8bE, we extended the scope of the study to controlling biofilm inhibition in medical devices like urinary catheters. The impact of catheter-associated infections on hospitalized patients is enormous. As per the CDC, approximately 75% of UTIs for hospitalized patients are associated with catheter and catheter-related bloodstream infection (CRBSI), which causes about 18.2% mortality each year (Zhong et al., 2021). Therefore, developing a molecule that can control the biofilm formation on urinary catheters is urgently needed. Here, we checked the effect of our lead molecule, **8bE**, on biofilm formation associated with viable but non-culturable (VBNC) *S. aureus* on urinary catheters **(** Figure 5) (Colomer-Winter et al., 2019). For this purpose, we opted to observe biofilm formation in the presence of our lead molecule, **8bE**, at different concentrations and compare it with the antibacterial drug molecule kanamycin and non-specific moiety trans-chalcone.

At higher concentrations (160 *μ*g/mL), kanamycin shows excellent inhibition of biofilm formation, which can be attributed to its potent antibacterial activity. However, as the concentration is decreased to 40 *μ*g/mL, the effect is reduced to ∼25%, whereas synthesized molecule **8bE** shows around 37% inhibition at this concentration, outperforming the reference standard kanamycin or trans-chalcone. This indicates the remarkable application of **8bE** against VBNC species.

### 3.5 Molecular docking studies

**FIGURE 6:**
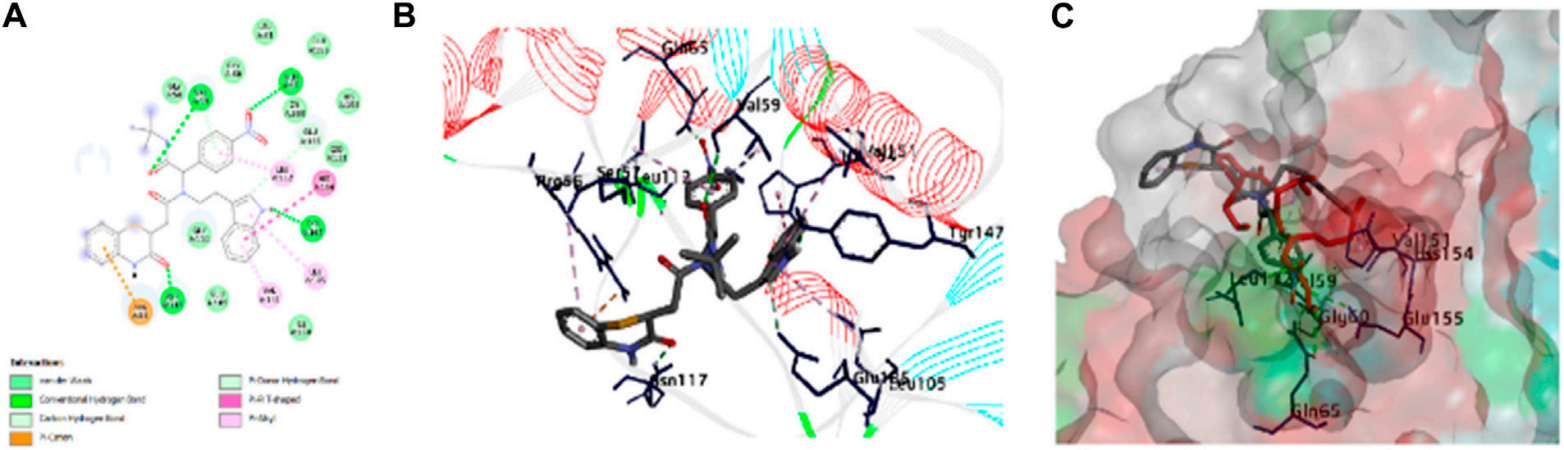
Molecular docking of compound 8bE against peptide deformylase. **(A)** 2D interaction image of compound 8bE within the PDF-binding pocket. The different interactions are highlighted in the figure; **(B)** 3D interaction image of compound 8bE within the PDF-binding pocket. Protein reported in line-ribbon. The blue stick model indicates the interacting amino acid residues. The elemental color stick model indicates the ligand (gray indicates hydrocarbons; blue indicates N; red indicates O; white indicates polar H; and yellow indicates S). **(C)** Superimposition of compound 8bE (elemental color stick model) and co-crystal ligand actinonin (red stick model) within the same binding pocket of the PDF receptor. The protein reported on the molecular surface.

We then attempted to find the interactions responsible for the activity of compounds. Among the actives, one is compound **8bE** (p-nitrophenyl substituent), which is highlighted here (Figure 6). Compound **8bE** has a binding energy of −8.3 kcal/mol, whereas the standard actinonin has a binding energy of −7.1 kcal/mol. It offers a strong hydrogen bond between one of the carbonyl oxygen atoms with –NH of Val:59, with a distance of 2.93 Å. One more hydrogen bond was observed between the carbonyl oxygen of the benzothiazine moiety with the –NH of Asn:117, having a distance of 2.27 Å. Another H-bond was observed between –N and O of the nitro with –NH of Gln:65, with a distance of 2.37 Å. The last H-bond had a distance of 1.87 Å between –NH of indole and the hydroxyl “O” of Tyr:147. Apart from van der Waals interaction, three pi–alkyl interactions were noted between the compound and Val:151, Leu:105, and Leu:112, respectively. One pi–pi T-shaped interaction was observed between the compound and the His:154 residue. A pi–cation interaction was also observed with the Arg:56 residue and two weak C-H bond interactions with amino acid residues Val:59 and Glu:185, respectively. The standard molecule, actinonin, shows five hydrogen bonds and two pi–alkyl interactions. All the molecules showed better docking interactions than the standard. Figure 6 highlights the 2D and 3D interaction images of compound **8bE** against *S. aureus* PDF. **8fE** (o-nitrophenyl substituent) has a low docking score of −7.5 kcal/mol. It shows a total of three hydrogen bonding interactions: one with –NH of Gly:110 and carbonyl oxygen of the molecule (2.90 Å), one with –C=O of Gly:110 and –NH of the benzothiazine part of the molecule (2.07 Å), and an H-bond with –N=O of the nitro and –NH of Arg:56 (2.23 Å). Other than van der Waals interaction, one pi–sigma interaction with Val:59; one pi–anion interaction with Glu:155; three pi–alkyl interactions with Val:59, Pro:78, and Leu:112; one pi–sulfur interaction with the modified cysteine residue Csd:111; and one weak carbon–hydrogen bond with Gly:110 were observed. Compound **8gE** (m-nitrophenyl substituent) has a comparable docking score of −8.4 kcal/mol. It showed two H-bonds with –N = O of nitro and –NH of both Val:59 (2.29 Å) and Gly:60 (2.32 Å). One H-bond with –NH of indole of the compound and hydroxyl–O- of Tyr:147 (1.77 Å); two H-bonds between C = O of benzothiazine of the compound and –NH of both Arg:56 (2.04 Å) and Asn:117 (2.91 Å); one pi–pi T-shaped interaction between the compound and His:154; four pi–alkyl/alkyl interactions with Leu:112, Val:59, Val:151, and Arg:56; and two weak carbon–hydrogen bonds with Gly:110 and Glu:185 were also observed. Compared to the interactions of actinonin, only three interacting amino acid residues were found similar with 8fE interactions, and all were weak interactions. With both 8bE and 8gE, five interactions were similar to actinonin, among which, in both cases, two were strong H-bonds. Detailed molecular modeling with other derivatives is provided in Supplementary Table S1 and Supplementary Figures S57 and S58. We observed that even small changes in the ligand molecule resulted in variations in the docking score. Upon comparing these docking results with the antimicrobial activity, we concluded that the molecules exhibiting strong activity (**8bE** and **8cE**) both interacted with Tyr:147 and His:154. These specific amino acids are part of the catalytic triad responsible for the deformylation process and are conserved across different organisms. It is noteworthy that in other organisms, Tyr:147 is substituted with Leu or Gly. This suggests that the synthesized molecules might have specificity toward Sa-PDF. Additionally, the authors of PDB ID: 1Q1Y noted that during attempts to crystallize, Cys:111 was oxidized to cysteine sulfonic acid (Cys-SO3H), mentioned as Csd:111 (Yoon et al., 2004). We observed that many of our molecules with high docking scores formed conventional hydrogen bonds with this modified amino acid. Providing strong interactions with catalytic triad amino acids and reducing off targeting interactions may show better activity and a good docking score. Some molecules like **8bB,** although having interactions with the catalytic triad, have more off-targeting interactions, thus leading to poor activity. We believe it is better to target the catalytic domains of target enzymes to improve the efficacy of lead molecules.

### 3.6 *In silico* prediction of pharmacokinetic and toxicity profiles

The pharmacokinetic properties of the molecules were predicted using the SwissADME web server (Daina et al., 2017), and the toxicity analysis was performed using the ProTox-II web server (Banerjee et al., 2018). The results indicated that actinonin is aqueous soluble (Log S = −2.28) with high gastrointestinal absorption, whereas compounds **8bE** (Log S = −6.16) and 8cE (Log S = −6.69) are poorly soluble with low gastrointestinal (GI) absorption. The skin permeability value (log kp in cm/s) for actinonin is −7.55, and for **8bE** and **8cE**, they are −6.54 and −5.90, respectively. The lower the skin permeability value, the lesser the skin permeability; hence, **8bE** and **8cE** have better skin permeability than actinonin. None of the molecules can penetrate the blood–brain barrier (BBB) similar to actinonin, thereby reducing the neurotoxicity, and thus, P-glycoprotein (P-gp) substrates (efflux pumps that protect the CNS from harmful xenobiotics) are safer molecules. Cytochrome P450 isoenzymes are important enzymes for drug metabolism, the inhibition of which may lead to drug accumulation in the body, causing adverse reactions (Das et al., 2023). Although actinonin does not inhibit any cytochrome isoenzymes, compound **8bE** shows inhibition for CYP2C19, CYP2C9, and CYP3A4 while not against CYP1A2 and CYP2D6, and compound 8cE inhibits all the cytochromes. All three have the same bioavailability score of 0.55. **8bE** and **8cE** fall under the type IV class of toxicity (2,000 < LD_50_ ≤ 5,000 mg/kg body weight), as per a globally harmonized system of classification of labeling of chemicals (OSHA, https://www.osha.gov/hazcom), similar to actinonin. Actinonin is non-hepatotoxic, non-mutagenic, and non-cytotoxic but active in carcinogenicity. **8bE** is active in carcinogenicity and mutagenicity but non-hepatotoxic and non-cytotoxic. **8cE** is non-hepatotoxic, non-mutagenic, non-carcinogenic, and also non-cytotoxic. Table 3 shows the properties in detail.

**TABLE 3:** Pharmacokinetics and toxicity profiling data.

S. No	Property	Actinonin	8bE	8cE
1	Log S (Aq solubility)	−2.28 (soluble)	−6.16 (poorly soluble)	−6.69 (poorly soluble)
2	Log Kp (skin permeability) cm/s	−7.55	−6.54	−5.90
3	BBB permeation	No	No	No
4	GI absorption	High	Low	Low
5	Bioavailability score	0.55	0.55	0.55
6	P-gp substrate	Yes	Yes	Yes
7	CYP1A2 inhibitor	No	No	Yes
8	CYP2C19 inhibitor	No	Yes	Yes
9	CYP2C9 inhibitor	No	Yes	Yes
10	CYP2D6 inhibitor	No	No	Yes
11	CYP3A4 inhibitor	No	Yes	Yes
12	Toxicity class	4	4	4
13	Hepatotoxicity	Inactive	Inactive	Inactive
14	Carcinogenicity	Active	Active	Inactive
15	Mutagenicity	Inactive	Active	Inactive
16	Cytotoxicity	Inactive	Inactive	Inactive

### 3.7 Molecular dynamics simulation data

**FIGURE 7:**
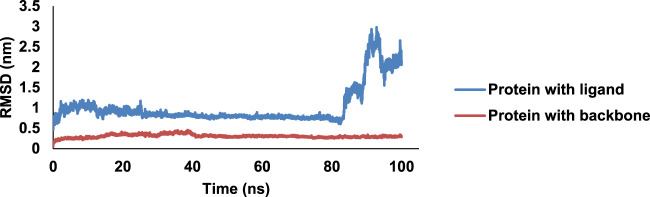
MD simulation data on the 8bE complex in 100-ns runtime; RMSD curve of the complex and protein.

One of the most active compounds, **8bE,** in complex with PDF was then subjected to MD simulations to determine the stability of the complex in a 100-ns MD runtime. RMSD is a measure of the protein–ligand complex stability. It determines the equilibration period of the MD runtime and denotes the dynamical behavior of both the protein and the ligand in the isobaric–isothermal simulation period (Sargsyan et al., 2017). The free protein with respect to the backbone has minimal deflections along the RMSD trajectory, initiating from 0.1 nm, having an average of approximately 0.3 nm, with an almost stable curve, throughout the 100-ns run. When the protein is in complex with ligand **8bE**, the RMSD curve drifts higher compared to the free protein, starting from 0.5 nm, indicating slightly more deviations. Initial instability was observed up to 27 ns; later, the curve stabilized rapidly with minimal or no deflections up to 83 ns (Figure 7). After 83 ns, large deflections and wobbles occur, determining the instability of the complex, with an RMSD increase to 2.5 nm after 90 ns. RMSF refers to the fluctuations or local changes that occur when a ligand is attached to a protein during the isobaric–isothermal MD runtime. More residue fluctuations can lead to poor ligand–protein binding and dissociation from the binding pocket during the MD runtime (Joshi et al., 2021). The protein–ligand complex has a lower RMSF value of 0.15 nm and reaches up to 0.7 nm. The RMSF curve represents a stable curve with minimal fluctuations up to residue number 1,050. A slight fluctuation occurs at around 1,220–1,386 residues and then stabilizes up to 2,500. Then, the RMSF curve shows major fluctuations. Overall, the RMSF curve stabilizes approximately at an average of 0.17 nm, indicating that ligand–protein binding is stable with minimal deflections. Rg assesses the structural compactness of the protein–ligand complex and decides whether the protein is stable or unstable based on fluctuation ranges during MD runtime. Rg values should not exhibit significant fluctuations during the MD run duration (Shahbaaz et al., 2019). The Rg curve is highly stable throughout the 100-ns runtime, having an average value of 1.7 nm. Hydrogen bonding (H-bonds) is the strongest bond involved in protein–ligand interaction, and it predicts the complex’s stability. The MD simulation relies heavily on hydrogen bonding. Greater the H-bonds, higher the stability of the complex throughout the MD runtime and *vice versa* (Das et al., 2023). During the 100-ns run, a maximum of 8 H-bonds were achieved within the 4-ns runtime. However, the complex stabilized and had a minimum of three H-bonds in average throughout the 100-ns runtime. After docking studies, four H-bonds were observed, and after MD simulation, three H-bonds were observed in average throughout the runtime, thus indicating a stable complex. The SASA is an important parameter of MD simulation study, which determines the surface of the protein accessible to solvent molecules when it forms a strong binding with the ligand and can thus predict the flexibility and important conformational changes occurring during the protein–ligand interaction (Marsh and Teichmann, 2011). The complex has a SASA range of an area of 102–116 nm^2^, with an average of 110 nm^2^. No major deflections were observed throughout the MD runtime; thus, the complex was bonded strongly (Supplementary Figures S59–S63 **)**.

## 4 Conclusion

In conclusion, based on computational design, we discovered a new series of (1,4)-benzothiazine-3-one containing bisamide derivatives as antibacterial and antibiofilm agents against *S. aureus*. Molecular modeling against peptide deformylase of *S. aureus* showed the important interactions of the molecule responsible for its activity. Furthermore, the application of these molecules is demonstrated in controlling the biofilm formation on urinary catheters. Further work to expand the scope of SAR (Structure Activity Relationship), toxicity, and *in vivo* applications is in progress, which will be explained soon.

## Data Availability

The original contributions presented in the study are included in the article/Supplementary Material; further inquiries can be directed to the corresponding author.
