# Supplementary material for: Design, synthesis, and evaluation of 1,4-benzothiazine-3-one containing bisamide derivatives as dual inhibitors of Staphylococcus aureus with plausible application in a urinary catheter
Source: Front Chem. 2024 Jun 26;12:1420593. doi: 10.3389/fchem.2024.1420593 (PMC11233542; doi:10.3389/fchem.2024.1420593)
Supplement: Supplementary file 1 [file DataSheet1.pdf]

Supporting information

Design, synthesis, and evaluation of 1,4-benzothiazine-3-one containing bis-amide derivatives as dual inhibitors of *Staphylococcus aureus* with plausible application in urinary catheter

Kaushal Naithani[1], Arka Das[1], Mamta Ushare[1], Subham Nath[1,2], Rashmita Biswas[1,2], Anirban Kundu[3], Kazi Tawsif Ahmed[4], Utpal Mohan[1,2], Subhendu Bhowmik[1]*

[1]*Department of Medicinal Chemistry, National Institute of Pharmaceutical Education and Research, 168 Maniktala Main Road, Kolkata-700054, West Bengal, India*

[2]*Microbiology Division, Department of Medicinal Chemistry, National Institute of Pharmaceutical Education and Research, 168 Maniktala Main Road, Kolkata-700054, West Bengal, India*

[3]*Department of Natural Product, National Institute of Pharmaceutical Education and Research, 168 Maniktala Main Road, Kolkata-700054, West Bengal, India*

[4] *Department of Botany, Visva Bharati University, Santiniketan, West Bengal, India*

Correspondence:
Dr. Subhendu Bhowmik
Email: sbhowmik@niperkolkata.edu.in

Table of Contents

General Information S3

Method for synthesis of 2-(3-oxo-3,4-dihydrobenzo[b][1,4]-thiazin-2-yl)-acetic acid S4

General procedure for synthesis of 8 S4

Characterization and analysis of synthesized compounds S5

Protocols for Microbiological Assay S19
- Bacterial Cell Viability Assay S20
- BIC determination by Crystal Violet Assay S20
- Catheter-Associated Biofilm formation S20

Copies of ^{1}H and ^{13}C spectra and docking datas S20

Supplementary References: S55

General Information:

Unless otherwise found, all starting materials and chemicals were obtained from commercial suppliers such as TCI and Merck and used as received without further purification. Thin-layer chromatography analyses were performed on pre-coated TLC plates (silica gel 60 HF254 - Merck 105554). TLC spots were visualized using Ultraviolet (UV) light at 254 nm and using ethanolic solution of PMA for the detection of the spots. Column chromatography was performed using Silica gel #230-400 using Hexane: EtOAc (9:1) to Hexane: EtOAc (1:1) as eluent. ^{1}H and ^{13}C NMR spectra were recorded on a JEOL-ECS400 spectrometer at 400 MHz and 100 MHz, respectively in deuterated solvents for example were recorded in $CDCl_3$, CD_3OD, or DMSO-d_6 obtained from CDH. ^{1}H and ^{13}C NMR chemical shifts were assigned in reference to tetramethyl silane [Si(CH3)$_4$] or residual solvent peak as an internal standard. Data for ^{1}H NMR are reported in the following order: chemical shift (δ) in ppm; multiplicities are indicated s (singlet), d (doublet), t (triplet), q (quartet), dd (doublet of doublets), dt (doublet of triplets), m (multiplet), bs (broad singlet); coupling constants (*J*) are obtained in Hertz (Hz). High-resolution mass spectra (HRMS) were recorded on Agilent Technologies, 1290 Infinity II UPLC System with Agilent 6545 Accurate-Mass Quadrupole Time-of-Flight (Q-TOF), with ion source Dual Agilent Jet Stream-Electrospray Ionization (Dual AJS-ESI) Technology. The operating mass parameters were run in positive ionization mode with Gas Temp., 320 °C; Gas flow, 8 L/min; Nebulizer, 35 psi; Sheath Gas Temp. and flow were 350 °C and 11 L/min, respectively. *S. aureus* MTCC 3160 was used as the reference pathogen. LB Culture media was obtained from Himedia. TD water was obtained from the In-house Milli Q system and sterilized via autoclave. Pre-sterilized microtiter culture plates were obtained from Genaxy. Absorbance was recorded using a synergy H1 hybrid multimode microplate reader. Readings were recorded at either 600 nm (for MIC determination) or 570 nm for crystal violet assay.

General Scheme

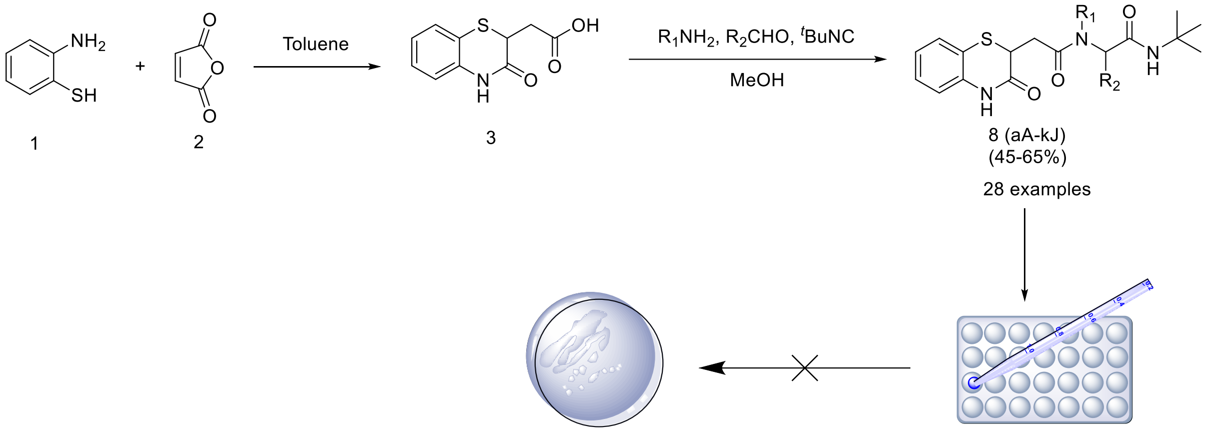

Method for synthesis of 2-(3-oxo-3,4-dihydrobenzo[*b*][1,4]-thiazin-2-yl)-acetic acid (3)

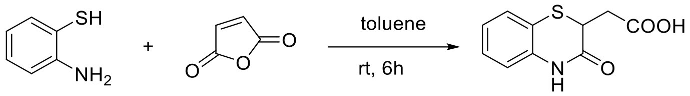

1 2 3

Dissolve Maleic Anhydride (2) (4.3g, 43.93 mM, 1.1eq) in toluene (~15 ml) and stir till a clear solution is obtained. Then add 2-amino-thiophenol (1) (5g, 39.94 mM, 1eq). Let the reaction stir for 6 h. till an off-white precipitate is obtained. Filter off the precipitate and wash it with $CHCl_3$. Dry at room temperature to remove traces of $CHCl_3$. The precipitate was used without further purification. The desired compound as white solid in 82% yield (7.13g). (Molteni et. al. 2004)

General procedure for synthesis of 8

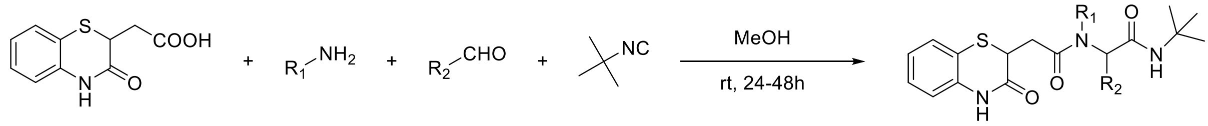

3 5 (a-k) 6 (A-J) 7 8((aA-kJ)

Where R_1 = Alkyl, Aryl; R_2 = Alkyl, Aryl or Heteroaryl (28 examples with 45-65% yield)

Amine derivative was dissolved in 5 ml reagent grade methanol and stirred until a clear solution was obtained. To this, carboxylic acid derivative, 3, was added resulting in a suspension. This suspension was cooled to 5 °C and *tert*-butyl isocyanide, 7, was added. To this mixture aldehyde derivative was added and the reaction was left to stir at room temperature (~30 °C) overnight.

<u>**NOTE**</u>: Reactions involving Furfuryl aldehyde, tryptamine, or phenyl ethyl amine require a temperature of 60 °C for completion of the reaction.
Workup: The reaction mixture is evaporated until a solid layer is obtained. It is then dissolved in $CHCl_3$ and washed with distilled water (3*15 ml), followed by brine solution (2*15 ml). The organic layer was then collected and dried using Sodium Sulphate. It is then evaporated in rota-vac until a thin film is obtained.

Characterization and analysis of synthesized compounds

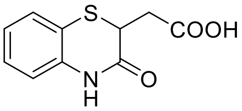

2-(3-oxo-3,4-dihydro-2*H*-benzo[*b*][1,4]-thiazin-2-yl)-acetic acid (3)

The reaction was carried out as mentioned above. Maleic Anhydride (2) (4.3g, 43.93 mM, 1.1 eq) was dissolved in toluene (~15 ml) and stir till a clear solution is obtained. Then add 2-amino-thiophenol (1) (5g, 39.94 mM, 1eq). Let the reaction stir for 6 h. till an off-white precipitate is obtained. Filter off the precipitate and wash it with $CHCl_3$. Dry at room temperature to remove traces of $CHCl_3$. The precipitate was used without further purification. The desired compound was obtained as white solid in 82% yield (7.13g), Percentage yield = 82% as off white solid, R_f = 0.1 (Hexane: EtOAc, 1:1); ^{1}H NMR (400 MHz, CD_3OD) δ: 7.29 (*d*, *J* =7.8hz, 1*H*), 7.18 (t, *J* =7.6hz, 1H),7.02 (t, *J*= 7.6hz, 1H), 6.97 (d, *J* =7.8hz, 1H), 3.86 (dd, *J*= 8Hz, 6.4Hz, 1H), 2.91 (dd, *J1*= 16.4Hz, *J2*= 6.3Hz, 1H), 2.52 (dd, *J*=16.4hz, 6hz, 1H); ^{13}C-NMR (100 MHz, CD_3OD) δ: 173.5, 168.6, 137.9, 129, 128.5, 124.9, 120.2, 118.4, 39.3, 34.9. [1]

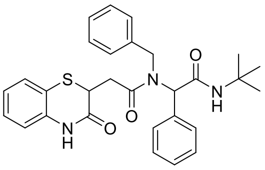

***N*-benzyl-N-(2-(*tert*-butylamino)-2-oxo-1-phenylethyl)-2-(3-oxo-3,4-dihydro-2*H*-benzo[*b*][1,4]-thiazin-2-yl)-acetamide (8aA)**

The reaction was carried out as mentioned in general procedure for synthesis of 8. Benzylamine (1eq, 0.448 mM, 50μl) was dissolved in 5 ml reagent grade methanol and stirred until clear solution was obtained. To this, 3 (1eq, 0.448 mM, 100 mg), was added resulting in a suspension. This suspension was cooled to 5°C and *tert*-butyl isocyanide (7) (1eq, 0.448 mM, 51 μl), was added. To this mixture, benzaldehyde (1eq, 0.448 mM, 46 μl) was added and the reaction was left to stir at room temperature (~30°C) overnight. The desired compound was obtained as pale brown solid in 60% yield (135mg) as, R_f = 0.3 (Hexane: EtOAc, 7:3); ^{1}H NMR (400 MHz, $CDCl_3$) δ: 8.77 (bs, 1H), 7.39 – 7.29 (m, 3H), 7.23-7.2 (m, 3H), 7.15 – 7.06 (m, 4H), 7.03-6.92 (m, 2H), 6.85– 6.81 (m, 2H), 6.01 – 5.86 (m, 2H), 4.79– 4.47 (m, 2H), 4.24 – 4.19 (m, 1H), 3.08-2.9(m, 1H), 2.63-2.43 (m, 1H), 1.33 (s, 9H); ^{13}C NMR (100 MHz, $CDCl_3$) δ: 171.1, 168.7, 167.5, 137.1, 136, 134.8, 129.7, 128.5,128.3, 128, 127.2, 126.8, 126, 125.8, 123.8, 119.7, 117.2, 63.2, 51.6, 49.8, 38.5, 33.4, 28.6; ES+HRMS calculated for $C_{29}H_{31}N_3O_3SNa$ = 524.1978, Obtained = 524.1997.

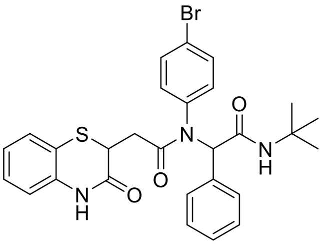

***N*-(4-bromophenyl)-*N*-(2-(tert-butylamino)-2-oxo-1-phenylethyl)-2-(3-oxo-3,4-dihydro-2*H*-benzo[*b*][1,4]-thiazin-2-yl)-acetamide (8aC)**

The reaction was carried out as mentioned in general procedure for synthesis of 8. 4-bromo-aniline (1eq, 0.448 mM, 77 mg) was dissolved in 5 ml reagent grade methanol and stirred until clear solution was obtained. To this, 3 (1eq, 0.448 mM, 100 mg), was added resulting in a suspension. This suspension was cooled to 5°C and *tert*-butyl isocyanide (7) (1eq, 0.448 mM, 51 µl), was added. To this mixture, benzaldehyde (1eq, 0.448 mM, 46 µl) was added and the reaction was left to stir at room temperature (~30 °C) overnight. The desired compound was obtained as pale yellow solid in 54% yield (137 mg), R_f = 0.3 (Hexane: EtOAc, 7:3); ^{1}H NMR (400 MHz, $CDCl_3$) δ: 8.67 (s, 1H), 7.68 (s, 1H), 7.26 (dd, J_1 = 7.79 Hz, J_2 =1.30 Hz, 1H), 7.20-7.10 (m, 6H), 6.98 (td, J_1 = 7.60 Hz, J_2 =1.27 Hz, 1H), 6.82 (dd, J_1 = 7.95 Hz, J_2 =1.07 Hz, 1H), 6.21-6.75 (m, 2H), 5.94-6.05 (m, 1H), 5.76-5.91 (m, 1H), 4.15 (dd, J = 8.02 Hz, 6.19 Hz, 1H), 2.76 (dd, J = 16.20 Hz, 6.19 Hz, 1H), 2.26 (q, J = 8.07 Hz, 1H), 1.31-1.36 (s, 9H); ^{13}C NMR (100 MHz, $CDCl_3$) δ: 169.8, 168.5, 167.3, 135.9, 134.3, 130.3, 128.4, 127.9, 127.3, 123.8, 119.5, 117.2, 65.5, 51.6, 38.5, 34.1, 28.6; ES+HRMS calculated for $C_{28}H_{28}BrN_3O_3SNa$ = 590.0912, Obtained = 590.0912.

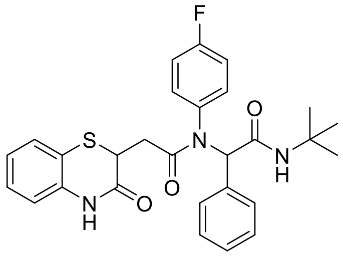

***N*-(*tert*-butyl)-2-(*N*-(4-fluorophenyl)-2-(3-oxo-3,4-dihydro-2*H*-benzo[*b*][1,4]-thiazin-2-yl)-acetamido)-2-phenylacetamide (8aD)**

The reaction was carried out as mentioned in general procedure for synthesis of 8. 4-fluoroaniline (1eq, 0.448 mM, 43µl) was dissolved in 5 ml reagent grade methanol and stirred until clear solution was obtained. To this, 3 (1eq, 0.448 mM, 100 mg), was added resulting in a suspension. This suspension was cooled to 5 °C and *tert*-butyl isocyanide (7) (1eq, 0.448 mM, 51 µl), was added. To this mixture, 4-methoxybenzaldehyde (1eq, 0.448 mM, 68 mg) was added and the reaction was left to stir at room temperature (~30 °C) overnight. The desired compound was obtained as grey solid in 51% yield (115mg), R_f = 0.3 (Hexane: EtOAc, 7:3); ^{1}H NMR (400 MHz, $CDCl_3$) δ: 8.67 (s, 1H), 7.27 (d, J = 1.4 Hz, 1H), 7.25 (d, J = 1.4 Hz, 1H), 7.24 – 7.13 (m, 4H), 7.13 – 7.07 (m, 3H), 6.98 (td, J_1 = 7.6, J_2 =1.3 Hz, 2H), 6.82 (dd, J_1 = 8.0, J_2 = 1.1 Hz, 1H), 6.00 (s, 1H), 5.85 (s, 1H), 4.15 (dd, J_1 = 8.0, J_2 = 6.2 Hz, 1H), 2.75 (dd, J_1 = 16.2, J_2 = 6.2 Hz, 1H), 2.26 (dd, J_1 = 16.2, J_2 = 8.0 Hz, 1H), 1.33 (s, 9H); ^{13}C NMR (100 MHz, $CDCl_3$) δ: 169.8, 168.5, 167.3, 163.2, 160.7, 135.9, 135.2, 135.1, 134.3, 130.3, 128.4, 127.9, 127.3, 123.8, 119.5, 117.2, 65.5, 51.7, 38.5, 34.1, 28.6; ES+HRMS calculated for $C_{28}H_{28}FN_3O_3SNa$ = 528.1733, Obtained = 528.1727.

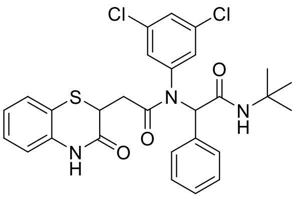

N-(tert-butyl)-2-(N-(3,5-dichlorophenyl)-2-(3-oxo-3,4-dihydro-2H-benzo[b][1,4]-thiazin-2-yl)-acetamido)-2-phenylacetamide (8aJ)

The reaction was carried out as mentioned in general procedure for synthesis of 8. 3,5-dichloroaniline (1eq, 0.448 mM, 72 mg) was dissolved in 5 ml reagent grade methanol and stirred until clear solution was obtained. To this, 3 (1eq, 0.448 mM, 100 mg), was added resulting in a suspension. This suspension was cooled to 5 °C and *tert*-butyl isocyanide (7) (1eq, 0.448 mM, 51 µl), was added. To this mixture, benzaldehyde (1eq, 0.448 mM, 46 µl) was added and the reaction was left to stir at room temperature (~30 °C) overnight. The product was obtained as white solid in 65% yield (162 mg), R_f = 0.3 (Hexane: EtOAc, 7:3); ^{1}H NMR (400 MHz, DMSO-d_6) δ: 10.57 (bs, 1H), 7.78 (s, 1H),7.30 (d, J =7.6Hz, 2H), 7.25-7.13 (m, 4H), 7.05 (d, J =8.4Hz, 2H), 6.98 (t, J = 7.2Hz, 2H), 6.87 (d, J = 7.6Hz, 1H), 6.41 (bs, 1H), 6.03 (s, 1H), 3.82 (dd, J_1 = 9.2Hz, J_2 = 4.4Hz, 1H), 2.56-2.49 (m, 1H), 2.11-2.04 (m, 1H), 1.20 (s, 9H); ^{13}C NMR (100 MHz, DMSO-d_6) δ: 168.38, 168.31, 165.9, 138.2, 136.6, 134.4, 132.5, 131.8, 131.5, 128.1, 127.9, 127.2, 123.2, 121.1, 117.8, 117.1, 63.0, 50.5, 38.0, 33.8, 28.4; ES+HRMS calculated for $C_{28}H_{27}Cl_2N_3O_3SNa$ = 578.1048, Obtained = 578.1040.

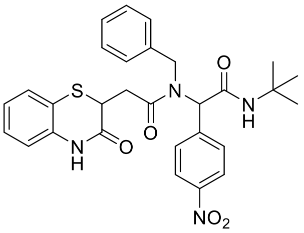

N-benzyl-N-(2-(tert-butylamino)-1-(4-nitrophenyl)-2-oxoethyl)-2-(3-oxo-3,4-dihydro-2H-benzo[b][1,4]-thiazin-2-yl)-acetamide (8bA)

The reaction was carried out as mentioned in general procedure for synthesis of 8. Benzylamine (1eq, 0.448 mM, 50 µl) was dissolved in 5 ml reagent grade methanol and stirred until clear solution was obtained. To this, 3 (1eq, 0.448 mM, 100 mg), was added resulting in a suspension. This suspension was cooled to 5 °C and *tert*-butyl isocyanide (7) (1eq, 0.448 mM, 51 µl), was added. To this mixture, 4-nitrobenzaldehyde (1eq, 0.448 mM, 68 mg) was added and the reaction was left to stir at room temperature (~30 °C) overnight. The desired compound was obtained as pale yellow solid in 60% yield (147mg), R_f = 0.3(Hexane: EtOAc, 7:3); ^{1}H NMR (400 MHz,$CDCl_3$) δ: 8.23 (d, J = 8.4 Hz, 2H), 8.09 (d, J = 31.5 Hz, 2H), 7.69 (d, J = 8.4 Hz, 2H), 7.49 (d, J = 7.5 Hz, 1H), 7.36 – 7.30 (m, 2H), 7.19 (d, J = 7.0 Hz, 1H), 7.08 (dt, J_1 = 13.4, J_2 = 7.5 Hz, 3H), 6.35 (d, J = 26.5 Hz, 1H), 4.68 (d, J = 18.0 Hz, 2H), 4.33 – 4.18 (m, 1H), 3.17 (dd, J_1 = 16.1, J_2 = 5.3 Hz, 1H), 2.90 (dd, J_1 = 16.1, J_2 = 8.6 Hz, 1H), 1.49 (s, 9H); ^{13}C NMR (100 MHz, $CDCl_3$) δ: 171.8, 168.7, 167.3, 147.6, 142.9, 137, 136.4, 130.5, 128.1, 127.8, 127.1, 126.8, 125.7, 123.7, 122.9, 120.1, 117.1, 113.2, 62.1, 51.3, 49.2, 38.7, 33.4, 27.4; ES+HRMS calculated for $C_{29}H_{30}N_4O_5SNa$ = 569.1829, Obtained = 569.1842.

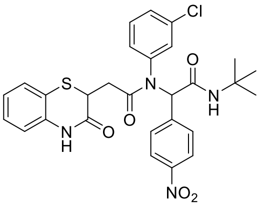

***N*-(*tert*-butyl)-2-(*N*-(3-chlorophenyl)-2-(3-oxo-3,4-dihydro-2*H*-benzo[*b*][1,4]-thiazin-2-yl)-acetamido)-2-(4-nitrophenyl)-acetamide (8bB)**

The reaction was carried out as mentioned in general procedure for synthesis of 8. 3-chloroaniline (1eq, 0.448 mM, 57 mg) was dissolved in 5 ml reagent grade methanol and stirred until clear solution was obtained. To this, 3 (1eq, 0.448 mM, 100 mg), was added resulting in a suspension. This suspension was cooled to 5 °C and *tert*-butyl isocyanide (7) (1eq, 0.448 mM, 51 µl), was added. To this mixture, 4-nitrobenzaldehyde (1eq, 0.448 mM, 68 mg) was added and the reaction was left to stir at room temperature (~30 °C) overnight. The desired compound was obtained as greyish-white solid in 55% yield (140mg), R_f = 0.3 (Hexane: EtOAc, 7:3); ^{1}H NMR (400 MHz, $CDCl_3$) δ: 8.86 (bs, 1H), 8.02 (d, *J* =7.2Hz, 2H), 7.38 (d, *J* =8.4Hz, 2H), 7.29-7.27 (m, 2H), 7.21 (d, *J* = 6.8Hz, 1H), 7.14-7.10 (m, 2H), 7.03-6.9 (m, 2H), 6.82 (d, *J* = 8Hz, 1H), 6.19 (s, 1H), 5.99 (bs, 1H), 4.15-4.11(m, 1H), 2.7 (q, *J* = 8Hz, 1H), 2.32 (dd, J_1 =16Hz, J_2=6.4Hz, 1H), 1.36 (s, 9H); ^{13}C NMR (100 MHz, $CDCl_3$) δ: 170, 167.3, 147.6, 141.4, 140.1, 135.8, 131.1, 130.3, 129.2, 128.5, 128, 127.4, 124.1, 123.3, 119.7, 117.2, 64.8, 52, 38.4, 33.9, 28.6; ES+HRMS calculated for $C_{28}H_{27}ClN_4O_5S$ =567.1361, Obtained = 567.1359.

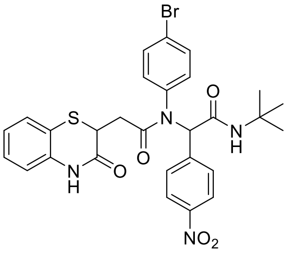

***N*-(4-bromophenyl)-*N*-(2-(*tert*-butylamino)-1-(4-nitrophenyl)-2-oxoethyl)-2-(3-oxo-3,4-dihydro-2*H*-benzo[*b*][1,4]-thiazin-2-yl)-acetamide (8bC)**

The reaction was carried out as mentioned in general procedure for synthesis of 8. 4-bromo-aniline (1eq, 0.448 mM, 77 mg) was dissolved in 5 ml reagent grade methanol and stirred until clear solution was obtained. To this, 3 (1eq, 0.448 mM, 100 mg), was added resulting in a suspension. This suspension was cooled to 5 °C and *tert*-butyl isocyanide (7) (1eq, 0.448 mM, 51 µl), was added. To this mixture, 4-nitrobenzaldehyde (1eq, 0.448 mM, 68 mg) was added and the reaction was left to stir at room temperature (~30°C) overnight. The product was obtained as pale yellow solid in 58% yield (148mg), R_f = 0.3 (Hexane: EtOAc, 7:3); ^{1}H NMR (400 MHz, $CDCl_3$) δ: 8.07-8.05 (m, 3H), 7.47-7.27 (m, 6H), 7.17 (t, *J* =7.6Hz, 1H), 7.01 (t, *J* = 8Hz, 1H), 6.82 (d, *J* =8 Hz, 1H), 6.32 (bs, 1H), 6.16 (s, 1H), 4.17 (d, *J*= 6Hz, 2Hz) 1H), 2.69 (dd, J_1 = 8.4Hz, J_2 = 7.6Hz, 1H), 2.28 (dd, J_1 = 10 Hz, J_2 = 6Hz), 1.39 (s, 9H); ^{13}C NMR (100 MHz, $CDCl_3$) δ: 170.1, 167.3, 167.1, 147.6, 141.5, 137.9, 135.8, 132.6, 131.8, 131.5, 131.3, 127.9, 127.4, 124, 123.4, 119.8, 117.2, 64.4, 52, 38.5, 33.8, 28.6; ES+HRMS calculated for $C_{28}H_{27}BrN_4O_5SNa$ = 633.0783, Obtained = 633.0812.

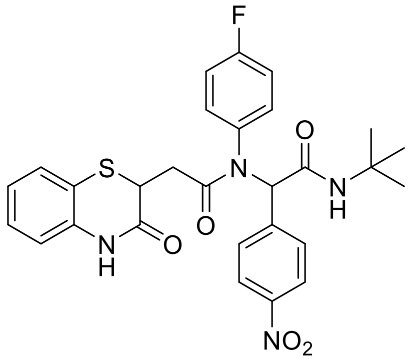

N-(*tert*-butyl)-2-(N-(4-fluorophenyl)-2-(3-oxo-3,4-dihydro-2H-benzo[*b*][1,4]-thiazin-2-yl)-acetamido)-2-(4-nitrophenyl)-acetamide (8bD)

The reaction was carried out as mentioned in general procedure for synthesis of 8. 4-fluoroaniline (1eq, 0.448 mM, 43 µl) was dissolved in 5 ml reagent grade methanol and stirred until clear solution was obtained. To this, 3 (1eq, 0.448 mM, 100 mg), was added resulting in a suspension. This suspension was cooled to 5 °C and *tert*-butyl isocyanide (7) (1eq, 0.448 mM, 51 µl), was added. To this mixture, 4-nitrobenzaldehyde (1eq, 0.448 mM, 68 mg) was added and the reaction was left to stir at room temperature (~30 °C) overnight. The desired compound was obtained as pale white solid in 56% yield (138 mg), R_f = 0.3 (Hexane: EtOAc, 7:3);^{1}H-NMR (400 MHz, $CDCl_3$) δ 8.28 (s, 1H), 8.04 (d, J = 8.86 Hz, 2H), 7.35 (d, J = 8.75 Hz, 2H), 7.28 (d, J = 1.26 Hz, 1H), 7.17-7.13 (m, 1H), 7.00 (td, J_1 = 7.63, J_2 = 1.24 Hz, 1H), 6.82 (dd, J_1 = 7.95, J_2 = 0.86 Hz, 1H), 6.35 (s, 1H), 6.16 (s, 1H), 4.16 (dd, J = 7.98, 6.37 Hz, 1H), 2.70 (q, J = 7.97 Hz, 1H), 2.28 (dd, J_1 = 15.87, J_2 = 6.32 Hz, 1H), 1.38 (s, 9H); ^{13}C NMR (100 MHz, $CDCl_3$) δ 170.4, 167.4, 167.1, 147.6, 141.6, 135.8, 131.8, 131.7, 131.3, 127.9, 127.5, 124.0, 123.3, 117.2, 64.4, 52.0, 38.5, 33.8, 28.6; ES+HRMS calculated for $C_{28}H_{27}FN_4O_5SNa$ = 551.1686, Obtained= 551.1653.

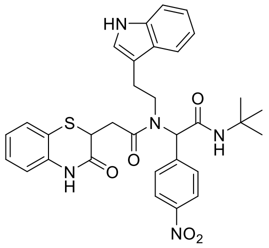

N-(2-(1H-indol-3-yl)-ethyl)-N-(2-(*tert*-butylamino)-1-(4-nitrophenyl)-2-oxoethyl)-2-(3-oxo-3,4-dihydro-2H-benzo[*b*][1,4]-thiazin-2-yl)-acetamide (8bE)

The reaction was carried out as mentioned in general procedure for synthesis of 8. Tryptamine (1eq, 0.448 mM, 72 mg) was dissolved in 5 ml reagent grade methanol and stirred until clear solution was obtained. To this, 3 (1eq, 0.448 mM, 100 mg), was added resulting in a suspension. This suspension was cooled to 5°C and *tert*-butyl isocyanide (4) (1eq, 0.448 mM, 51 µl), was added. To this mixture, 4-nitrobenzaldehyde (1eq, 0.448 mM, 68 mg) was added and the reaction was left to stir at 60 °C overnight. The desired compound was obtained as a greyish-white solid in 64% yield (172mg), R_f = 0.15 (Hexane: EtOAc, 7:3); ^{1}H-NMR (400 MHz, $CDCl_3$) δ: 8.42-8. (m, 4H), 7.63-7.57 (m, 2H), 7.31-7.29 (m, 2H), 7.16-7.01 (m, 4H), 6.85-6.77 (m, 2H), 6.47 (bs,1H), 5.89 (s, 1H), 4.24-4.19 (m, 1H), 3.86-3.50 (m, 2H), 3.12-2.50 (m, 4H); ^{13}C-NMR (100 MHz, $CDCl_3$) δ: 170.9, 167.8, 167.2, 147.4, 143.1, 136.1, 135.9,

130.0, 129.6, 128.0, 127.5, 126.7, 125.0, 124.2, 123.6, 122.3, 122.1, 120.0, 119.7, 118.2, 117.2, 111.5, 111.3, 63.3, 51.9, 48.4, 38.6, 32.7, 31.9, 28.6; ES+HRMS calculated for $C_{32}H_{33}N_5O_5SNa$ = 622.2055, Obtained = 622.2056.

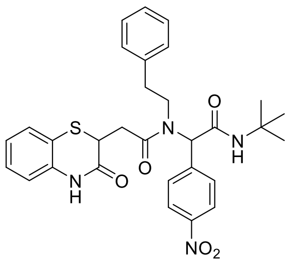

***N*-(*tert*-butyl)-2-(4-nitrophenyl)-2-(2-(3-oxo-3,4-dihydro-2*H*-benzo[*b*][1,4]-thiazin-2-yl)-*N*-phenethylacetamido)-acetamide (8bF)**

The reaction was carried out as mentioned in general procedure for synthesis of 8. Phenylethyl amine (1eq, 0.448 mM, 56µl) was dissolved in 5 ml reagent grade methanol and stirred until clear solution was obtained. To this, 3 (1eq, 0.448 mM, 100 mg), was added resulting in a suspension. This suspension was cooled to 5 °C and *tert*-butyl isocyanide (7) (1eq, 0.448 mM, 51 µl), was added. To this mixture, 4-nitrobenzaldehyde (1eq, 0.448 mM, 68 mg) was added and the reaction was left to stir at 60 °C overnight. The product was obtained as grey solid in 60% yield (151mg), R_f = 0.2 (Hexane: EtOAc, 7:3); ^{1}H-NMR (400 MHz, $CDCl_3$) δ: 8.82 (bs, 1H), 8.25-8.14 (m, 3H), 7.62 (dd, J_1 = 10.8Hz, J_2 =8.8 Hz, 2H), 7.3 (t, J =6.8Hz, 1H), 7.19-7.14 (m, 5H), 7.03-6.99 (m, 1H), 6.89-6.84 (m, 3H), 6.52 (bs, 1H), 5.99 (s, 1H), 4.22 (t, J =7Hz, 1H), 3.70-3.47 (m, 2H), 3.13-3.06 (m, 1H), 2.91-2.31 (m, 3H), 1.39 (s, 9H); ^{13}C NMR (100 MHz, $CDCl_3$) δ: 170.8, 167.6, 167.3, 147.5, 143.0, 137.3, 137.1, 135.9, 129.9, 129.6, 128.7, 128.4, 128.0, 127.5, 127.1, 126.8, 124.2, 123.9, 123.7, 119.7, 117.3, 62.9, 51.9, 49.2, 38.7, 36.6, 32.5, 28.5; ES+HRMS calculated for $C_{30}H_{32}N_4O_5S$ = 561.2093, Obtained = 561.2061.

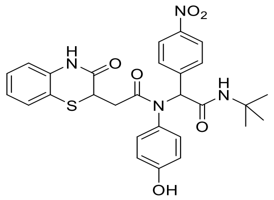

***N*-(*tert*-butyl)-2-(*N*-(4-hydroxyphenyl)-2-(3-oxo-3,4-dihydro-2*H*-benzo[*b*][1,4]-thiazin-2-yl)-acetamido)-2-(4-nitrophenyl)-acetamide (8bI)**

The reaction was carried out as mentioned in general procedure for synthesis of 8. 4-aminophenol (1eq, 0.448 mM, 49 mg) was dissolved in 5 ml reagent grade methanol and stirred until clear solution was obtained. To this, 3 (1eq, 0.448 mM, 100 mg), was added resulting in a suspension. This suspension was cooled to 5 °C and *tert*-butyl isocyanide (7) (1eq, 0.448 mM, 51 µl), was added. To this mixture, 4-nitrobenzaldehyde (1eq, 0.448 mM, 68 mg) was added and the reaction was left to stir at room temperature (~30 °C) overnight. The product was obtained as pale brown solid in 55% yield (155mg), R_f = 0.1 (Hexane: EtOAc, 7:3); ^{1}H NMR (400 MHz, $CDCl_3$) δ: 8.06 – 8.03 (m, 3H), 7.43-7.27 (m, 3H), 7.18-7.13 (m, 1H), 7.03-6.99 (m, 1H), 6.81-6.78 (m, 1H), 6.52-6.29 (m, 2H), 6.14-5.94 (m, 2H), 4.19-4.10 (m, 1H), 2.80-2.72 (m, 1H), 2.34-2.23 (m, 1H), 1.40 (s, 9H); ^{13}C NMR (100 MHz, $CDCl_3$) δ: 170.9, 168.0, 167.4, 156.6, 147.5, 141.7, 135.7, 131.2, 131.1, 131.0, 130.9, 130.8, 127.9, 127.4, 124.1,

123.2, 120.0, 117.3, 116.2, 65.3, 52.1, 38.4, 33.7, 28.6; ES+HRMS calculated for $C_{28}H_{28}N_4O_6SNa$ = 571.1627, Obtained = 571.1631.
`

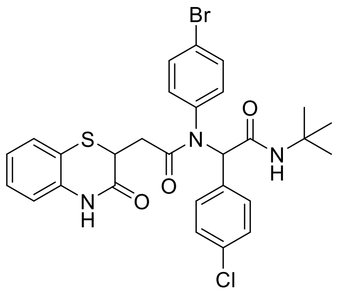

N-(4-bromophenyl)-N-(2-(*tert*-butylamino)-1-(4-chlorophenyl)-2-oxoethyl)-2-(3-oxo-3,4-dihydro-2*H*-benzo[*b*][1,4]-thiazin-2-yl)-acetamide (8cC)

The reaction was carried out as mentioned in general procedure for synthesis of 8. 4-bromo-aniline (1eq, 0.448 mM, 77mg) was dissolved in 5 ml reagent grade methanol and stirred until clear solution was obtained. To this, 3 (1eq, 0.448 mM, 100 mg), was added resulting in a suspension. This suspension was cooled to 5°C and *tert*-butyl isocyanide (7) (1eq, 0.448 mM, 51 µl), was added. To this mixture, 4-nitrobenzaldehyde (1eq, 0.448 mM, 68 mg) was added and the reaction was left to stir at room temperature (~30°C) overnight. The product was obtained as pale white solid in 52% yield (140mg), R_f = 0.3 (Hexane: EtOAc, 7:3); ^{1}H-NMR (400 MHz, $CDCl_3$) δ: 8.96 (d, J = 9.38 Hz, 1H), 7.28 (dd, J_1 = 7.86, J_2 =1.34 Hz, 1H), 7.14-7.08 (m, 4H), 7.05 (d, J = 8.52 Hz, 2H), 6.99 (td, J_1 = 7.58, J_2 = 1.26 Hz, 1H), 6.82 (d, J = 7.78 Hz, 1H), 5.96 (d, J = 18.52 Hz, 2H), 4.09-4.18 (m, 1H), 2.68 (dd, J_1 = 16.09, J_2 =7.46 Hz, 1H), 2.27 (dd, J_1 = 16.07, J_2 = 6.58 Hz, 1H), 1.32 (s, 9H); ^{13}C-NMR (100 MHz, $CDCl_3$) δ 170.0, 168.2, 167.4, 135.9, 134.4, 132.8, 132.1, 132.0, 132.0, 131.6, 128.5, 127.8, 127.3, 123.8, 119.3, 117.3, 64.5, 51.7, 38.5, 34.0, 28.5; ES+HRMS calculated for $C_{28}H_{27}BrClN_3O_3SNa$ =622.0543, Obtained = 622.0554.

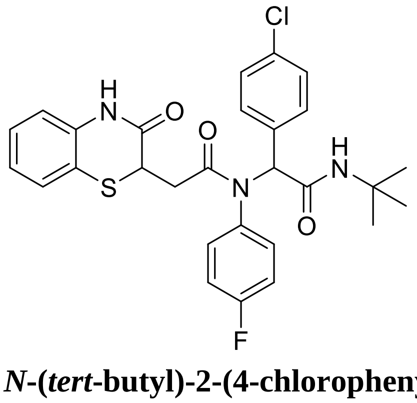

N-(*tert*-butyl)-2-(4-chlorophenyl)-2-(N-(4-fluorophenyl)-2-(3-oxo-3,4-dihydro-2*H*-benzo[*b*][1,4]-thiazin-2-yl)-acetamido)-acetamide (8cD)

The reaction was carried out as mentioned in general procedure for synthesis of 8. 4-fluoroaniline (1eq, 0.448 mM, 43 µl) was dissolved in 5 ml reagent grade methanol and stirred until clear solution was obtained. To this, 3 (1eq, 0.448 mM, 100 mg), was added resulting in a suspension. This suspension was cooled to 5 °C and *tert*-butyl isocyanide (7) (1eq, 0.448 mM, 51 µl), was added. To this mixture, 4-chlorobenzaldehyde (1eq, 0.448 mM, 63 mg) was added and the reaction was left to stir at room temperature (~30 °C) overnight. The desired

compound was obtained as pale white solid in 51% yield (121 mg), R_f = 0.2 (Hexane: EtOAc, 7:3); ^{1}H-NMR (400 MHz, $CDCl_3$) δ: 8.06 (d, *J* = 24.53 Hz, 1H), 7.28 (d, *J* = 1.07 Hz, 1H), 7.19-7.13 (m, 3H), 7.07 (d, *J* = 8.48 Hz, 1H), 7.00 (td, J_1 = 7.60, J_2 = 1.20 Hz, 1H), 6.80 (dd, J_1 = 7.95, J_2 = 1 Hz, 1H), 5.99 (d, *J* = 4.28 Hz, 1H), 4.15 (t, *J* = 7.11 Hz, 1H), 2.72 (dd, J_1 = 16.09, J_2 = 7.22 Hz, 1H), 2.25 (dd, J_1 = 16.05, J_2 =7.03 Hz, 1H), 1.35 (s, 9H); ^{13}C NMR (100 MHz, $CDCl_3$) δ: 169.8, 168.0, 167.0, 138.1, 135.8, 134.5, 132.8, 131.9, 131.7, 128.6, 128.0, 127.4, 123.9, 119.8, 117.1, 64.5, 51.8, 38.5, 33.9, 28.6; ES+HRMS calculated for $C_{28}H_{27}ClFN_3O_3SNa$= 562.1343, Obtained = 562.1328.

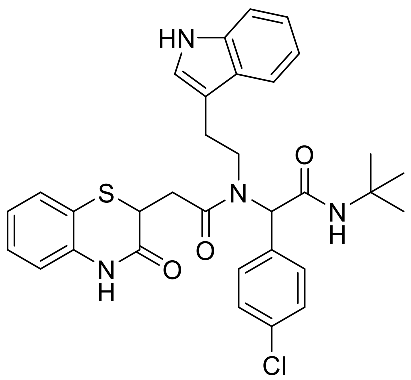

_N_-(2-(1_H_-indol-3-yl)-ethyl)-_N_-(2-(_tert_-butylamino)-1-(4-chlorophenyl)-2-oxoethyl)-2-(3-oxo-3,4-dihydro-2_H_-benzo[_b_][1,4]-thiazin-2-yl)-acetamide (8cE)

The reaction was carried out as mentioned in general procedure for synthesis of 8. Tryptamine (1eq, 0.448 mM, 72 mg) was dissolved in 5 ml reagent grade methanol and stirred until clear solution was obtained. To this, 3 (1eq, 0.448 mM, 100 mg), was added resulting in a suspension. This suspension was cooled to 5 °C and *tert*-butyl isocyanide (7) (1eq, 0.448 mM, 51 µl), was added. To this mixture, 3-nitrobenzaldehyde (1eq, 0.448 mM, 68 mg) was added and the reaction was left to stir at 60 °C overnight. The desired compound was obtained as off-white solid in 62% yield(mg), R_f = 0.25 (Hexane: EtOAc, 7:3); ^{1}H-NMR (400 MHz, $CDCl_3$) δ: 8.73 (bs, 1H), 7.44-7.29 (m, 5H), 7.19-7.14 (m, 4H), 7.02-6.91 (m, 1H), 6.90-6.76 (m, 1H), 6.21-5.94 (m, 2H), 4.25 (q, *J* =8.8Hz, 1H), 3.58-3.40 (m, 2H), 3.12-3.08 (m, 1H), 2.74-2.49 (m, 2H), 2.23-2.15 (m, 1H), 1.36 (s, 9H); ^{13}C-NMR (100 MHz, $CDCl_3$) δ: 170.4, 168.3, 167.4, 137.7, 135.9, 134.4, 134.2, 133.9, 131.1, 130.8, 128.9, 128.6, 128.4, 128.1 127.5, 126.7, 126.6, 124.2, 119.7, 117.2, 61.9, 51.7, 48.3, 38.7, 36.3, 32.7, 28.6; ES+HRMS calculated for $C_{32}H_{33}ClN_4O_3SNa$ = 611.1854, Obtained = 611.1864.

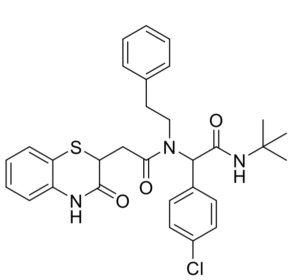

_N_-(_tert_-butyl)-2-(4-chlorophenyl)-2-(2-(3-oxo-3,4-dihydro-2_H_-benzo[b][1,4]-thiazin-2-yl)-_N_-phenethylacetamido)-acetamide (8cF)

The reaction was carried out as mentioned in general procedure for synthesis of 8. Phenylethyl amine (1eq, 0.448 mM, 56 µl) was dissolved in 5 ml reagent grade methanol and stirred until clear solution was obtained. To this, 3 (1eq, 0.448 mM, 100 mg), was added resulting in a suspension. This suspension was cooled to 5 °C and *tert*-butyl isocyanide (7) (1eq, 0.448 mM,

51 µl), was added. To this mixture, 4-chlorobenzaldehyde (1eq, 0.448 mM, 63 mg) was added and the reaction was left to stir at room temperature (~30 °C) overnight. The product was obtained as white solid in 55% yield (134mg), R_f = 0.25 (Hexane: EtOAc, 7:3); ^{1}H NMR (400 MHz, $CDCl_3$) δ: 9.16 (bs, 1H), 8.25 (s, 1H), 7.41-7.28 (m, 5H), 7.13-7.11 (m, 3H), 7.04-6.94 (m, 2H), 6.90-6.87 (m, 1H), 6.63 (dd, J_1 =10Hz, J_2 =2Hz, 1H), 6.24 (bs, 1H), 5.93 (s, 1H), 4.23-4.19 (m, 1H), 3.62-3.47 (m, 2H), 3.08-2.56 (m, 3H), 2.46-2.40 (m, 1H), 1.36 (s, 9H); ^{13}C NMR (100 MHz, $CDCl_3$) δ: 170.5, 168.6, 167.5, 136.0, 134.4, 134.1, 133.9, 130.9, 128.9, 127.9, 127.4, 126.8, 124.0, 122.0, 119.5, 118.2, 117.3, 111.7, 111.2, 62.5, 51.7, 47.5, 38.6, 32.8, 28.6, 25.8; ES+HRMS calculated for $C_{30}H_{32}ClN_3O_3S$ = 550.1811, Obtained = 550.1816.

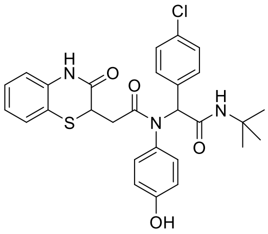

***N*-(*tert*-butyl)-2-(4-chlorophenyl)-2-(*N*-(4-hydroxyphenyl)-2-(3-oxo-3,4-dihydro-2*H*-benzo[*b*][1,4]-thiazin-2-yl)-acetamido)-acetamide (8cI)**

The reaction was carried out as mentioned in general procedure for synthesis of 8. 4-aminophenol (1eq, 0.448 mM, 49 mg) was dissolved in 5 ml reagent grade methanol and stirred until clear solution was obtained. To this, 3 (1eq, 0.448 mM, 100 mg), was added resulting in a suspension. This suspension was cooled to 5 °C and *tert*-butyl isocyanide (7) (1eq, 0.448 mM, 51 µl), was added. To this mixture, 4-chlorobenzaldehyde (1eq, 0.448 mM, 63 mg) was added and the reaction was left to stir at room temperature (~30 °C) overnight. The product was obtained as off white solid with 55% yield (108mg), R_f = 0.1 (Hexane: EtOAc, 7:3); ^{1}H NMR (400 MHz, $CDCl_3$) δ: 9.07 (bs, 1H), 7.35 – 7.26 (m, 1H), 7.20 (d, J = 7.6, 1H), 7.12 – 7.04 (m, 5H), 6.95 (t, J =7.4Hz, 1H), 6.82-6.39 (m, 3H), 6.21 (bs, 1H), 5.87 (s, 1H), 4.14 – 4.07 (m, 1H), 2.80-2.71 (m, 1H), 2.36-2.27 (m, 1H), 1.31 (s, 9H);^{13}C NMR (100 MHz, $CDCl_3$) δ: 170.8, 168.8, 167.6, 156.7, 135.7, 134.3, 132.9, 131.6, 130.8, 128.4, 127.8, 127.3, 123.9, 119.6, 117.4, 60.5, 51.9, 51.8, 38.4, 28.5; ES+HRMS calculated for $C_{28}H_{28}ClN_3O_4SNa$ = 560.1387, Obtained = 560.1390.

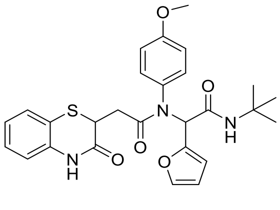

***N*-(*tert*-butyl)-2-(furan-2-yl)-2-(*N*-(4-methoxyphenyl)-2-(3-oxo-3,4-dihydro-2*H*-benzo[*b*][1,4]-thiazin-2-yl)-acetamido)-acetamide (8dG)**

The reaction was carried out as mentioned in general procedure for synthesis of 8. 4-methoxyaniline (1eq, 0.448mM, 55mg) was dissolved in 5ml reagent grade methanol and stirred until clear solution was obtained. To this, 3 (1eq, 0.448 mM, 100 mg), was added resulting in a suspension. This suspension was cooled to 5 °C and *tert*-butyl isocyanide (7) (1eq, 0.448 mM, 51 µl), was added. To this mixture, furfuraldehyde (1eq, 0.448 mM, 37 µl)

was added and the reaction was left to stir at 60 °C overnight. The desired compound was obtained as pale-brown solid in 48% yield (109mg), R_f = 0.25 (Hexane: EtOAc, 7:3); ^{1}H -NMR (400 MHz, $CDCl_3$) δ: 8.86 (bs, 1H), 7.28-7.25 (m, 3H), 7.12-7.10 (m, 1H), 7.00-6.98 (m, 1H), 6.83-6.81 (m, 1H), 6.72-6.56 (m, 2H), 6.30-6.25 (m, 2H), 6.18-6.15 (m, 2H), 4.16-4.11 (m, 1H), 3.73 (s, 3H), 2.78-2.71 (m, 1H), 2.35-2.27 (m, 1H), 1.37 (s, 9H); ^{13}C NMR (100 MHz, $CDCl_3$) δ: 170.3, 167.5, 166.5, 159.3, 147.8, 142.3, 135.9, 131.6, 127.9, 127.2, 123.7, 119.8, 117.2, 114.2, 112.1, 112.0, 110.6, 59.0, 55.3, 51.6, 38.5, 33.7, 28.6; ES+HRMS calculated for $C_{27}H_{29}N_3O_5S$ = 508.1861, Obtained = 508.1854.

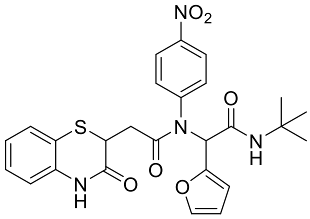

***N*-(*tert*-butyl)-2-(furan-2-yl)-2-(*N*-(4-nitrophenyl)-2-(3-oxo-3,4-dihydro-2*H*-benzo[*b*][1,4]-thiazin-2-yl)-acetamido)-acetamide (8dH)**

The reaction was carried out as mentioned in general procedure for synthesis of 8. 4-nitroaniline (1eq, 0.448 mM, 62 mg) was dissolved in 5 ml reagent grade methanol and stirred until clear solution was obtained. To this, 3 (1eq, 0.448 mM, 100 mg), was added resulting in a suspension. This suspension was cooled to 5°C and *tert*-butyl isocyanide (7) (1eq, 0.448 mM, 51 µl), was added. To this mixture, furfuraldehyde (1eq, 0.448 mM, 37 µl) was added and the reaction was left to stir at 60 °C overnight. The desired compound was obtained as reddish-brown solid in 48% yield (112 mg), R_f = 0.3 (Hexane: EtOAc, 7:3); ^{1}H-NMR (400 MHz, $CDCl_3$) δ: 8.06 (m, 3H), 7.86 (s, 1H), 7.30-7.26 (m, 2H), 7.17-7.13 (m, 1H), 7.03-6.98 (m, 1H), 6.77 (d, J = 8.00 Hz, 1Hz), 6.30-6.17 (m, 3H), 6.04 (s, 1H), 4.16 (t, J = 7.06 Hz, 1H), 2.71 (dd, J_1 = 16.07Hz, J_2 =7.09 Hz, 1H), 2.28 (dd, J = 16.04 Hz, 7.06 Hz, 1H), 1.37 (s, 9H); ^{13}C NMR (100 MHz, $CDCl_3$) δ: 169, 167.2, 166.1, 147.3, 146.9, 145.0, 143.2, 135.8, 131.2, 128.0, 127.3, 124.1, 124.0, 119.7, 117.2, 112.6, 110.8, 58.9, 51.9, 38.2, 33.8, 29.7, 28.5; ES+HRMS calculated for $C_{26}H_{26}N_4O_6SNa$ = 545.1471, Obtained = 545.1484.

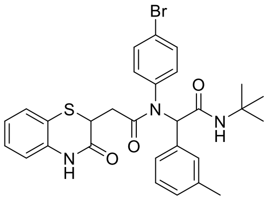

***N*-(4-bromophenyl)-*N*-(2-(*tert*-butylamino)-2-oxo-1-(*m*-tolyl)-ethyl)-2-(3-oxo-3,4-dihydro-2*H*-benzo[*b*][1,4]-thiazin-2-yl)-acetamide (8eC)**

The reaction was carried out as mentioned in general procedure for synthesis of 8. 4-bromo-aniline (1eq, 0.448 mM, 77 mg) was dissolved in 5 ml reagent grade methanol and stirred until clear solution was obtained. To this, 3 (1eq, 0.448 mM, 100 mg), was added resulting in a suspension. This suspension was cooled to 5 °C and *tert*-butyl isocyanide (7) (1eq, 0.448 mM, 51 µl), was added. To this mixture, 3-methylbenzaldehyde (1eq, 0.448 mM, 68 mg) was added and the reaction was left to stir at room temperature (~30 °C) overnight. The product was obtained as pale yellow solid in 52% yield (135mg), R_f = 0.3 (30% EtOAc in Hexane); ^{1}H NMR (400 MHz, $CDCl_3$) δ: 8.42 (s, 1H), 7.43 – 7.27 (m, 1H), 7.13 (td, J = 7.7, 1.4 Hz, 1H),

7.06 (t, J = 7.5 Hz, 1H), 7.02 – 6.96 (m, 3H), 6.93 (s, 1H), 6.89 (d, J = 7.5 Hz, 2H), 6.80 (d, J = 7.9 Hz, 2H), 5.94 (s, 1H), 5.82 (s, 1H), 4.21 – 4.11 (m, 1H), 2.77 (dd, J = 16.2, 6.3 Hz, 1H), 2.26 (dd, J = 16.3, 7.9 Hz, 1H), 2.22 (s, 3H), 1.34 (s, 9H); ^{13}C-NMR (100 MHz, $CDCl_3$) δ: 169.8, 168.6, 167.3, 138.0, 135.8, 135.3, 134.2, 132.1, 131.0, 129.1, 128.2, 127.9, 127.3, 123.8, 119.7, 117.1, 65.6, 51.6, 38.5, 34.0, 28.6, 21.2; ES+HRMS calculated for $C_{29}H_{30}BrN_3O_3S$ = 580.1225, Obtained = 580.1200.

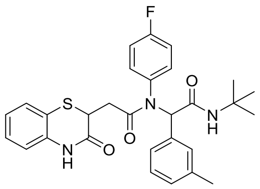

N-(*tert*-butyl)-2-(N-(4-fluorophenyl)-2-(3-oxo-3,4-dihydro-2H-benzo[*b*][1,4]-thiazin-2-yl)-acetamido)-2-(*m*-tolyl)-acetamide (8eD)

The reaction was carried out as mentioned in general procedure for synthesis of 8. 4-fluoroaniline (1eq, 0.448 mM, 43 µl) was dissolved in 5 ml reagent grade methanol and stirred until clear solution was obtained. To this, 3 (1eq, 0.448 mM, 100 mg), was added resulting in a suspension. This suspension was cooled to 5 °C and *tert*-butyl isocyanide (7) (1eq, 0.448 mM, 51 µl), was added. To this mixture, 4-chlorobenzaldehyde (1eq, 0.448 mM, 63 mg) was added and the reaction was left to stir at room temperature (~30 °C) overnight. The desired compound was obtained as pale brown solid in 55% yield (127 mg), R_f = 0.3 (Hexane: EtOAc, 7:3); ^{1}H-NMR (400 MHz, $CDCl_3$) δ: 8.69 (s, 1H), 7.25 (d, J = 1.30 Hz, 1H), 7.13 (t, J = 7.68 Hz, 1H), 7.08-6.96 (m, 3H), 6.94 (s, 1H), 6.89 (d, J = 7.41 Hz, 1H), 6.82 (dd, J_1 = 7.99 Hz, J_2 =1.11 Hz, 1H), 5.93 (s, 1H), 5.80 (s, 1H), 4.15 (dd, J_1 = 7.91 Hz, J_2 =6.23 Hz, 1H), 2.77 (dd, J_1 = 16.24 Hz, J_2 = 6.23 Hz, 1H), 2.28-2.22 (m, 4H), 1.32 (s, 9H); ^{13}C NMR (100 MHz, $CDCl_3$) δ: 169.8, 168.7, 167.5, 163.2, 160.7, 138.0, 136.0, 135.2, 134.1, 132.4, 131.0, 129.1, 128.2, 127.9, 127.3, 123.8, 120.0, 117.1, 115.7, 115.5, 65.4, 51.6, 38.3, 28.6, 21.2; ES+HRMS calculated for $C_{29}H_{30}FN_3O_3SNa$ =542.1890, Obtained = 542.1899.

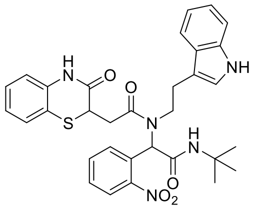

N-(2-(1H-indol-3-yl)-ethyl)-N-(2-(*tert*-butylamino)-1-(2-nitrophenyl)-2-oxoethyl)-2-(3-oxo-3,4-dihydro-2H-benzo[*b*][1,4-]-thiazin-2-yl)-acetamide (8fE)

The reaction was carried out as mentioned in general procedure for synthesis of 8. Tryptamine (1eq, 0.448 mM, 72 mg) was dissolved in 5 ml reagent grade methanol and stirred until clear solution was obtained. To this, 3 (1eq, 0.448 mM, 100 mg), was added resulting in a suspension. This suspension was cooled to 5°C and *tert*-butyl isocyanide (7) (1eq, 0.448 mM, 51 µl), was added. To this mixture, 2-nitrobenzaldehyde (1eq, 0.448 mM, 68 mg) was added and the reaction was left to stir at 60 °C overnight. The desired compound was obtained as pale brown solid in 59% yield (158 mg), R_f = 0.15 (Hexane: EtOAc, 7:3); ^{1}H-NMR (400 MHz, $CDCl_3$) δ: 8.5-8.18 (m, 3H), 7.63 (dd, J_1 = 8.4Hz, J_2 = 2.4Hz, 2H), 7.33-7.3 (m, 1H), 7.22-7.17 (m, 4H), 7.02 (t, J =7.6Hz, 1H), 6.91-6.87 (m, 3H), 6.43 (bs, 1H), 5.95 (s, 1H), 4.26-4.21 (m, 1H), 3.71-3.48 (m, 2H), 3.14-3.07 (m, 2H), 2.93-2.38 (m, 3H), 1.40 (s, 9H);^{13}C NMR (100

MHz, $CDCl_3$) δ: 170.8, 167.6, 167.3, 147.5,143.1, 137.2, 135.9, 130, 129.7, 128.7, 128.5, 128.1, 127.6, 126.9, 124.2, 123.7, 117.3, 63.0, 51.9, 49.2, 38.7, 36.1, 32.5, 28.6; ES+HRMS calculated for $C_{32}H_{33}N_5O_5SNa$ = 622.2094, Obtained = 622.2103.

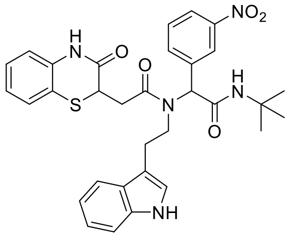

***N*-(2-(1*H*-indol-3-yl)-ethyl)-*N*-(2-(*tert*-butylamino)-1-(3-nitrophenyl)-2-oxoethyl)-2-(3-oxo-3,4-dihydro-2*H*-benzo[*b*][1,4]-thiazin-2-yl)-acetamide (8gE)**

The reaction was carried out as mentioned in general procedure for synthesis of 8. Tryptamine (1eq, 0.448 mM, 72 mg) was dissolved in 5 ml reagent grade methanol and stirred until clear solution was obtained. To this, 3 (1eq, 0.448 mM, 100 mg), was added resulting in a suspension. This suspension was cooled to 5 °C and *tert*-butyl isocyanide (7) (1eq, 0.448 mM, 51 µl), was added. To this mixture, 3-nitrobenzaldehyde (1eq, 0.448 mM, 68 mg) was added and the reaction was left to stir at 60 °C overnight. The desired compound was obtained as pale brown solid in 55% yield (148mg), R_f =0.15 (Hexane: EtOAc, 7:3); ^{1}H-NMR (400 MHz, $CDCl_3$) δ: 8.20-8.05 (m, 3H), 7.60 (q, *J* =7.2Hz, 2H), 7.31-7.28 (m, 2H), 7.16-7.01 (m, 5H), 6.86-6.80 (m, 2H),6.43 (bs, 1H), 5.91 (s, 1H), 4.24-4.19 (m, 1H), 3.81-3.54 (m, 2H), 3.12-2.51 (m, 4H), 1.39 (s, 9H); ^{13}C NMR (100 MHz, $CDCl_3$) δ: 170.9, 167.9, 167.3, 148.1, 137.9, 137.7, 136.0, 135.8, 135.2, 134.9, 134.3, 129.4, 127.9, 127.4, 124.1, 123.8, 122.9, 122.1, 119.5, 119.3 118.0, 117.5, 111.3, 62.6, 53.4, 47.9, 38.7, 32.6, 30.8, 28.5; ES+HRMS calculated for $C_{32}H_{33}N_5O_5SNa$ = 622.2100, Obtained = 622.2110.

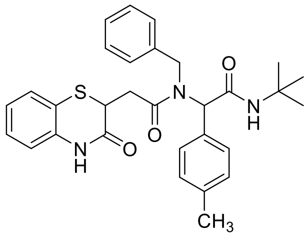

***N*-benzyl-*N*-(2-(*tert*-butylamino)-2-oxo-1-(*p*-tolyl)-ethyl)-2-(3-oxo-3,4-dihydro-2*H*-benzo[*b*][1,4]-thiazin-2-yl)-acetamide (8hA)**

The reaction was carried out as mentioned in general procedure for synthesis of 8. Benzylamine (1eq, 0.448 mM, 50 µl) was dissolved in 5ml reagent grade methanol and stirred until clear solution was obtained. To this, 3 (1eq, 0.448 mM, 100 mg), was added resulting in a suspension. This suspension was cooled to 5 °C and *tert*-butyl isocyanide (7) (1eq, 0.448 mM, 51 µl), was added. To this mixture, 4-methylbenzaldehyde (1eq, 0.448 mM, 46 µl) was added and the reaction was left to stir at room temperature (~30 °C) overnight. The desired compound was obtained as off white solid in 65% yield (150mg) as, R_f = 0.3 (Hexane: EtOAc, 7:3); ^{1}H NMR (400 MHz, $CDCl_3$) δ: 9.25 (bs, 1H), 7.30-7.28 (m, 1H), 7.25-7.22 (m, 2H), 7.10 – 7.06 (m, 5H), 7.01 – 6.91 (m, 3H), 6.87-6.82 (m, 2H), 5.99-5.92 (m, 2H) , 4.75 – 4.35 (m, 2H), 4.21-4.17 (m, 1H), 3.08 – 2.88 (m, 1H), 2.50-2.44 (m, 1H), 2.25 (s, 3H), 1.31 (*s*, 9*H*); ^{13}C NMR (100 MHz, $CDCl_3$) δ: 171.2, 168.9, 167.5, 138.2, 137.2, 135.9, 131.8, 129.7, 129.5, 129.2, 128.6, 128.3, 128, 128, 127.4, 127.2, 126.8, 126.1, 125.9, 123.8, 117.2, 117.1, 63.3, 51.6, 49.7, 38.6, 38.4, 33.5, 28.6, 21; ES+HRMS calculated for $C_{30}H_{33}N_3O_3SNa$ = 538.2135, Obtained = 538.2151.

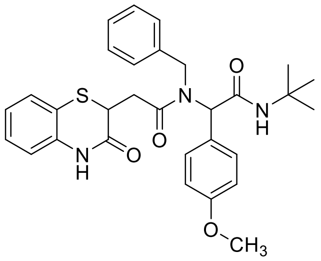

***N*-benzyl-*N*-(2-(*tert*-butylamino)-1-(4-methoxyphenyl)-2-oxoethyl)-2-(3-oxo-3,4-dihydro-2*H*-benzo[*b*][1,4]-thiazin-2-yl)-acetamide (8iA)**

The reaction was carried out as mentioned in general procedure for synthesis of 8. Benzylamine (1eq, 0.448 mM, 50 µl) was dissolved in 5 ml reagent grade methanol and stirred until clear solution was obtained. To this, 3 (1eq, 0.448 mM, 100 mg), was added resulting in a suspension. This suspension was cooled to 5 °C and *tert*-butyl isocyanide (7) (1eq, 0.448 mM, 51 µl), was added. To this mixture, 4-methoxybenzaldehyde (1eq, 0.448 mM, 46 µl) was added and the reaction was left to stir at room temperature (~30 °C) overnight. The desired compound was obtained as pale brown solid in 62% yield (147mg), R_f = 0.3 (Hexane: EtOAc, 7:3); ^{1}H NMR (400 MHz, $CDCl_3$) δ: 9.23 (bs, 1H), 7.27-7.26 (m, 2H), 7.25-7.24 (m, 1H), 7.13-7.06 (m, 4H), 7.01 – 6.96 (m, 1H), 6.94-6.91 (m, 1H), 6.88– 6.83 (m, 2H), 6.73 – 6.67 (m, 2H), 6.01 – 5.83 (m, 2H), 4.75 – 4.44 (m, 2H), 4.22 – 4.09 (m, 1H), 3.70 (s, 3H), 3.09-2.88 (m, 1H), 2.61-2.44 (m, 1H), 1.32 (s, 9H); ^{13}C NMR (100 MHz, $CDCl_3$) δ: 171.3, 171.1, 169, 169, 167.6, 159.5, 137.3, 137.2, 136.1, 135.9, 131.1, 131, 128.3, 127.9, 127.2, 127.2, 126.8, 126.7, 126.7, 126, 125.8, 123.8, 123.8, 119.9, 119.4, 117.3, 117.2, 113.9, 113.9, 99.9, 62.8, 62.4, 60.4, 55.2, 51.5, 49.5, 38.5, 38.3, 33.5, 33.4, 28.6; ES+HRMS calculated for $C_{30}H_{33}N_3O_4SNa$ = 554.2084, Obtained =554.2092.

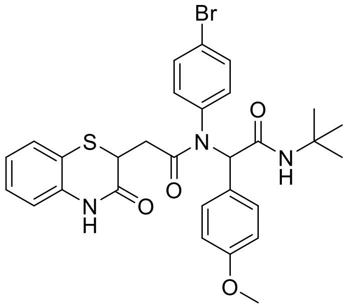

***N*-(4-bromophenyl)-*N*-(2-(*tert*-butylamino)-1-(4-methoxyphenyl)-2-oxoethyl)-2-(3-oxo-3,4-dihydro-2*H*-benzo[*b*][1,4]-thiazin-2-yl)-acetamide (8iC)**

The reaction was carried out as mentioned in general procedure for synthesis of 8. 4-bromo-aniline (1eq, 0.448 mM, 77 mg) was dissolved in 5 ml reagent grade methanol and stirred until clear solution was obtained. To this, 3 (1eq, 0.448 mM, 100 mg), was added resulting in a suspension. This suspension was cooled to 5 °C and *tert*-butyl isocyanide (7) (1eq, 0.448 mM, 51 µl), was added. To this mixture, benzaldehyde (1eq, 0.448 mM, 46 µl) was added and the reaction was left to stir at room temperature (~30 °C) overnight. The desired compound was obtained as pale yellow solid in 58% yield (155mg), R_f = 0.3 (Hexane: EtOAc, 7:3); ^{1}H NMR (400 MHz, $CDCl_3$) δ; 8.52 (s, 1H), 7.25 (d, J = 1.4 Hz, 1H), 7.24 (d, J = 1.3 Hz, 1H), 7.12 (td, J_1 = 7.7 Hz, J_2 = 1.4 Hz, 1H), 7.05 – 6.93 (m, 4H), 6.82 – 6.76 (m, 1H), 6.71 – 6.67 (m, 2H), 5.94 (s, 1H), 5.78 (s, 1H), 5.28 (s, 2H), 4.13 (dd, J_1 = 7.9, J_2 = 6.3 Hz, 1H), 3.73 (s, 3H), 2.73

(dd, J_1 = 16.2 Hz, J_2 = 6.3 Hz, 1H), 2.22 (dd, J_1 = 16.2 Hz, J_2 =7.9 Hz, 1H), 1.31 (s, 9H);^{13}C NMR (100 MHz, $CDCl_3$) δ: 169.5, 168.7, 167.2, 159.5, 138.3, 135.8, 132.1, 131.6, 127.9, 127.3, 126.1, 123.8, 122.4, 119.5, 117.2, 113.8, 64.7, 55.2, 53.4, 51.6, 38.5, 28.6; ES+HRMS calculated for $C_{29}H_{30}BrN_3O_4SNa$ =620.1018, Obtained = 620.1018.

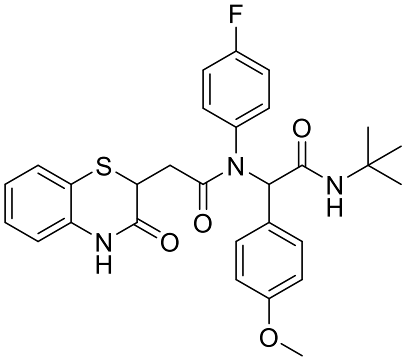

***N*-(*tert*-butyl)-2-(*N*-(4-fluorophenyl)-2-(3-oxo-3,4-dihydro-2*H*-benzo[*b*][1,4]-thiazin-2-yl)-acetamido)-2-(4-methoxyphenyl)-acetamide (8iD)**

The reaction was carried out as mentioned in general procedure for synthesis of 8. 4-fluoroaniline (1eq, 0.448 mM, 43μl) was dissolved in 5 ml reagent grade methanol and stirred until clear solution was obtained. To this, 3 (1eq, 0.448 mM, 100 mg), was added resulting in a suspension. This suspension was cooled to 5°C and *tert*-butyl isocyanide (7) (1eq, 0.448 mM, 51 µl), was added. To this mixture, 4-methoxybenzaldehyde (1eq, 0.448 mM, 68 mg) was added and the reaction was left to stir at room temperature (~30 °C) overnight. The desired compound was obtained as pale brown solid in 55% yield (132 mg), R_f = 0.3 (Hexane: EtOAc, 7:3); ^{1}H NMR (400 MHz, $CDCl_3$) δ 8.21 (s, 1H), 7.29 – 7.27 (m, 1H), 7.16 – 7.11 (m, 1H), 7.05 – 6.94 (m, 5H), 6.79 (d, J = 8.0 Hz, 1H), 6.69 (d, J = 8.8 Hz, 3H), 5.97 (s, 1H), 5.82 (s, 1H), 4.17 – 4.13 (m, 1H), 3.74 (s, 3H), 2.75 (dd, J_1 = 16.2 Hz, J_2 =6.3 Hz, 1H), 2.24 (dd, J_1 = 16.2 Hz, J_2 =7.9 Hz, 1H), 1.33 (s, 9H);^{13}C NMR (100 MHz, $CDCl_3$) δ: 169.7, 168.8, 167.2, 160.7, 159.4, 135.8, 135.2, 132.2, 131.6, 12, 127.3, 126.3, 123.8, 119.6, 117.1, 113.7, 64.7, 55.2, 51.6, 38.5, 34, 28.6; ES+HRMS calculated for $C_{29}H_{30}FN_3O_4SNa$ = 558.1839, Obtained = 558.1840.

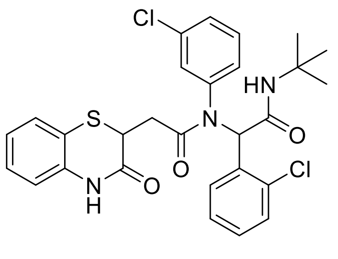

***N*-(*tert*-butyl)-2-(2-chlorophenyl)-2-(*N*-(3-chlorophenyl)-2-(3-oxo-3,4-dihydro-2*H*-benzo[*b*][1,4]-thiazin-2-yl)-acetamido)-acetamide (8jB)**

The reaction was carried out as mentioned in general procedure for synthesis of 5. 3-chloroaniline (1eq, 0.448 mM, 57 mg) was dissolved in 5 ml reagent grade methanol and stirred until clear solution was obtained. To this, 3 (1eq, 0.448 mM, 100 mg), was added resulting in a suspension. This suspension was cooled to 5°C and *tert*-butyl isocyanide (7) (1eq, 0.448 mM, 51 µl), was added. To this mixture, 2-chlorobenzaldehyde (1eq, 0.448 mM, 63 mg) was added

and the reaction was left to stir at room temperature (~30 °C) overnight. The desired compound was obtained as white solid in 45% yield (112mg), R_f = 0.3 (Hexane: EtOAc, 7:3); ^{1}H NMR (400 MHz, $CDCl_3$) δ: 9.12 (bs, 1H), 7.76 – 7.27 (m, 2H), 7.13 – 6.94 (m, 8H), 6.86 – 6.82 (m, 2H), 6.32 (s, 1H), 5.94 (bs, 1H), 4.17 – 4.12 (m, 1H), 2.81-2.70 (m, 1H), 2.36 – 2.25 (m, 1H); ^{13}C NMR (100 MHz, $CDCl_3$) δ: 169.5, 168.1, 167.6, 140.0, 135.9, 135.3, 131.4, 129.9, 129.4, 128.6, 127.9, 127.2, 126.7, 123.8, 119.8, 119.2, 117.3, 62.1, 51.9, 51.8, 38.4, 28.7; ES+HRMS calculated for $C_{28}H_{27}Cl_2N_3O_3SNa$ =578.1048, Obtained = 578.1039.

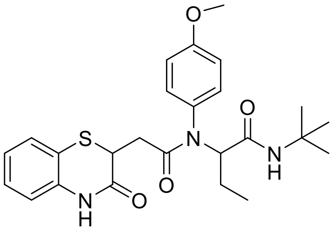

***N*-(*tert*-butyl)-2-(*N*-(4-methoxyphenyl)-2-(3-oxo-3,4-dihydro-2*H*-benzo[*b*][1,4]-thiazin-2-yl)-acetamido)-butanamide (8kG)**

The reaction was carried out as mentioned in general procedure for synthesis of 8. 4-methoxyaniline (1eq, 0.448 mM, 55 mg) was dissolved in 5 ml reagent grade methanol and stirred until clear solution was obtained. To this, 3 (1eq, 0.448 mM, 100 mg), was added resulting in a suspension. This suspension was cooled to 5 °C and *tert*-butyl isocyanide (7) (1eq, 0.448 mM, 51 µl), was added. To this mixture, Propionaldehyde (1eq, 0.448 mM, 32 µl) was added and the reaction was left to stir at room temperature (~30 °C) overnight. The desired compound was obtained as pale brown solid in 52% yield (109 mg), R_f = 0.35 (Hexane: EtOAc,7:3); ^{1}H NMR (400 MHz, $CDCl_3$) δ: 8.34 (bs, 1H), 7.30 – 7.28 (m, 1H), 7.21-7.14 (m, 2H), 7.06-6.99 (m, 1H), 6.90-6.79 (m, 4H), 6.55 (bs, 1H), 4.90-4.87 (m, 1H), 4.14-4.10 (m, 1H), 3.78 (s, 3H), 2.63 (dd, J_1 =16.0Hz, J_2 =7.2Hz, 1H), 2.33 (dd, *J1* = 16.0Hz, *J2*= 7.2Hz, 1H), 1.84 – 1.6 (m, 2H), 1.37 (s, 9H), 0.89 (t, *J* =7.2Hz, 3H);^{13}C NMR (100 MHz, $CDCl_3$) δ: 170.8, 169.6, 167.2, 159.4, 135.9, 130.7, 127.9, 127.3, 123.8, 119.8, 117.1, 114.5, 60.8, 55.4, 51.2, 38.4, 33.8, 28.6, 21.5, 10.8; ES+HRMS calculated for $C_{25}H_{31}N_3O_4SNa$ = 492.1933, Obtained = 492.1938.

Protocols for Microbiological Assay

Protocols used here are a step-by-step adjustment and follow guidelines as described by CLSI (Clinical and Laboratory Standards Institute, USA).

Media: Although, MHB (Mueller-Hinton Broth) supplemented with 2% w/v NaCl is recommended, we used LB (Luria-Bertani, Miller) Broth without any supplements.

Bacteria: The assays were performed on MTCC 3160 S. aureus which was cultured in LB broth 24 hrs before the experiment from a previously prepared bacterial colony plate. It was normalized to 0.08-0.13OD_{600} as a density equivalent to 10^8CFU/ml. This was used for inoculation.

Preparation of test molecules: All molecules were dissolved in DMSO to obtain a stock solution of 5 mg/ml. Before the experiment, aliquots of different concentrations were prepared by diluting the required quantity with dd water. To avoid any effect of DMSO its concentration was kept below 1% at each well.

Bacterial Cell Viability Assay

Experimental Procedure: 100µL of 1mg/mL drug solution was added to 100µL of LB broth in the first column of a 48 well plate and two-fold serial dilution was performed thereafter. Then, 100µL of bacterial suspension (10^6 CFU/mL) was added to each well to form a final of $5X10^5$ CFU/mL. Well with no drug is used as control. The plate was kept at an incubator at 37°C for 16 hours. The optical density of bacterial cultures was measured at 600nm using Multiskan SkyHigh Microplate Spectrophotometer (Thermo). Data are presented as mean standard deviation with n=6.[2]

BIC determination by Crystal Violet Assay

Experimental Procedure: In a pre-sterilized microtiter plate, add 75µl of pre-sterilized Luria Bertani broth (Miller). To this 20 µl of previously prepared aliquots of drug/molecule was added with vigorous pipetting to ensure complete solubilization. To this add 5µl of bacterial culture (OD= 0.01) and incubate the plate at 37°C for 16 to 18 hrs. Carefully pipette out culture media and wash with 1x PBS (100µl *3). Then add 100µl solution of 4% paraformaldehyde in dd water. Let it incubate for 30 min. Then carefully remove the solution and wash it with 1x PBS (100µl *3). Then add 105µl of 0.1% crystal violet solution. Let it incubate for 30 min and then wash with 1x PBS (100µl *3). Leave it to dry for 30 min so that no traces of crystal violet solution are left. In most cases, a fine layer of violet-coloured film will be visible.
Then add 105µl Ethanol and Incubate for 10min. (a 33% solution of acetic acid in dd water can also be used but it requires incubation of 30 min). Read absorbance at 570nm.[3]

Catheter-Associated Biofilm formation:
Experimental Procedure: Natural Rubber latex silicone catheter was used for this assay. The catheter's circular disks (0.2 cm height and 0.25 cm diameter) were excised and kept in long-range (350nm) UV radiation 12 hr before the experiment. These were incubated in 48 well microtiter plate along with cultured *S. aureus* (0.01 OD_{600}) and test molecule for 24 hr at 37 °C under static conditions. After 24 hr. crystal violet assay was used to quantify biofilm formation.[4]

Copies of ^{1}H and ^{13}C spectra

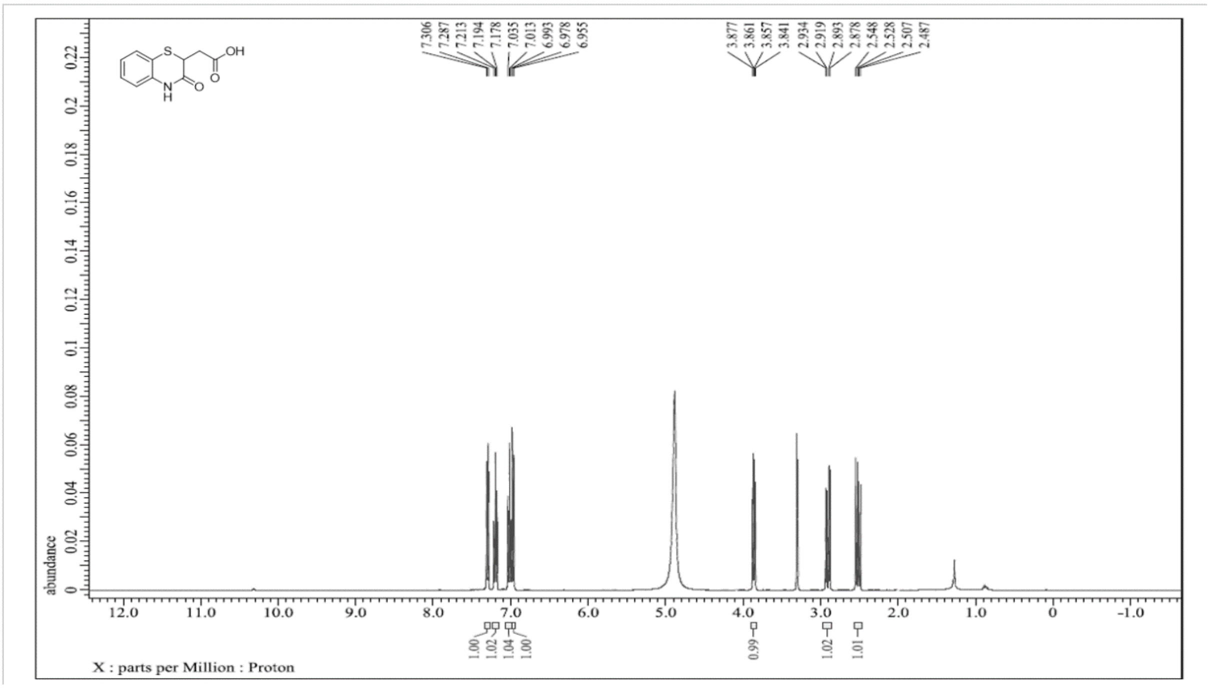

Figure S1: ^{1}H spectra of 2-(3-oxo-3,4-dihydro-2*H*-benzo[b][1,4]-thiazin-2-yl)-acetic acid (**3**)

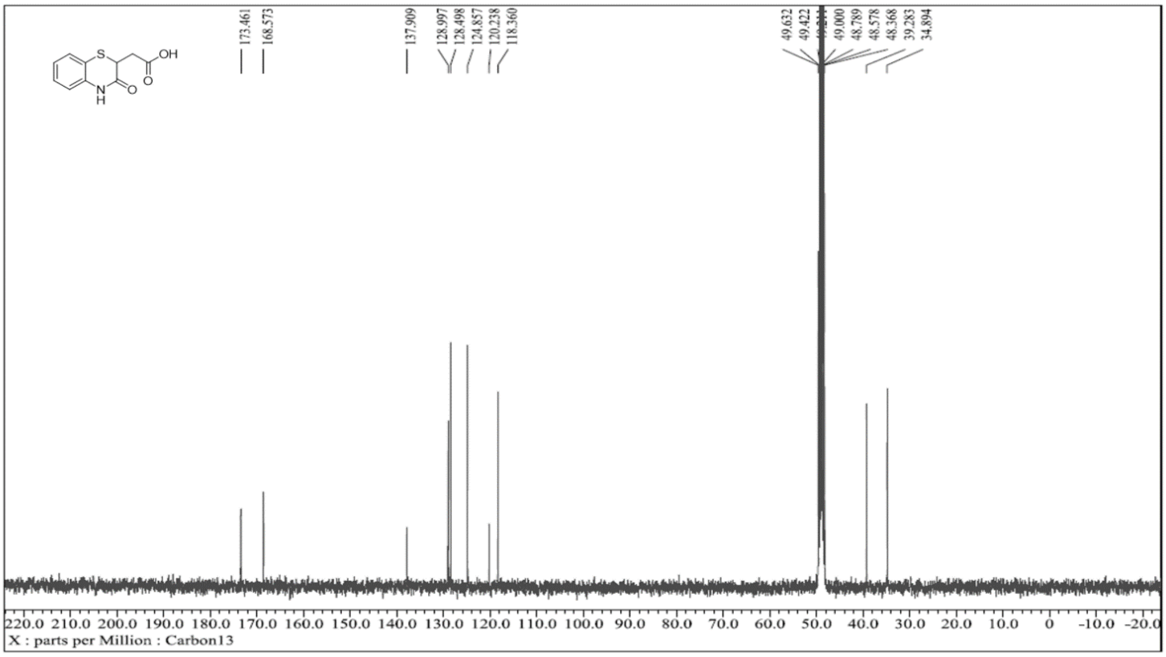

Figure S2: ^{13}C spectra of 2-(3-oxo-3,4-dihydro-2*H*-benzo[*b*][1,4]-thiazin-2-yl)-acetic acid (**3**)

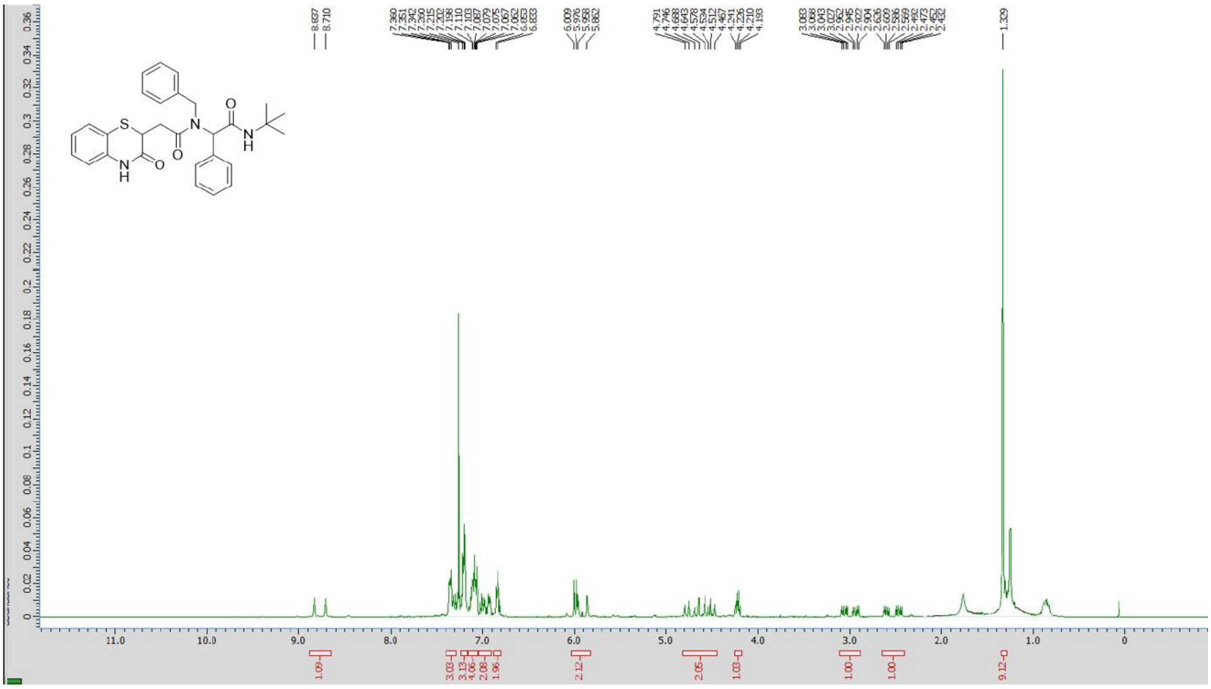

Figure S3: ^{1}H spectra of *N*-benzyl-*N*-(2-(*tert*-butylamino)-2-oxo-1-phenylethyl)-2-(3-oxo-3,4-dihydro-2*H*-benzo[*b*][1,4]-thiazin-2-yl)-acetamide (**8aA**)

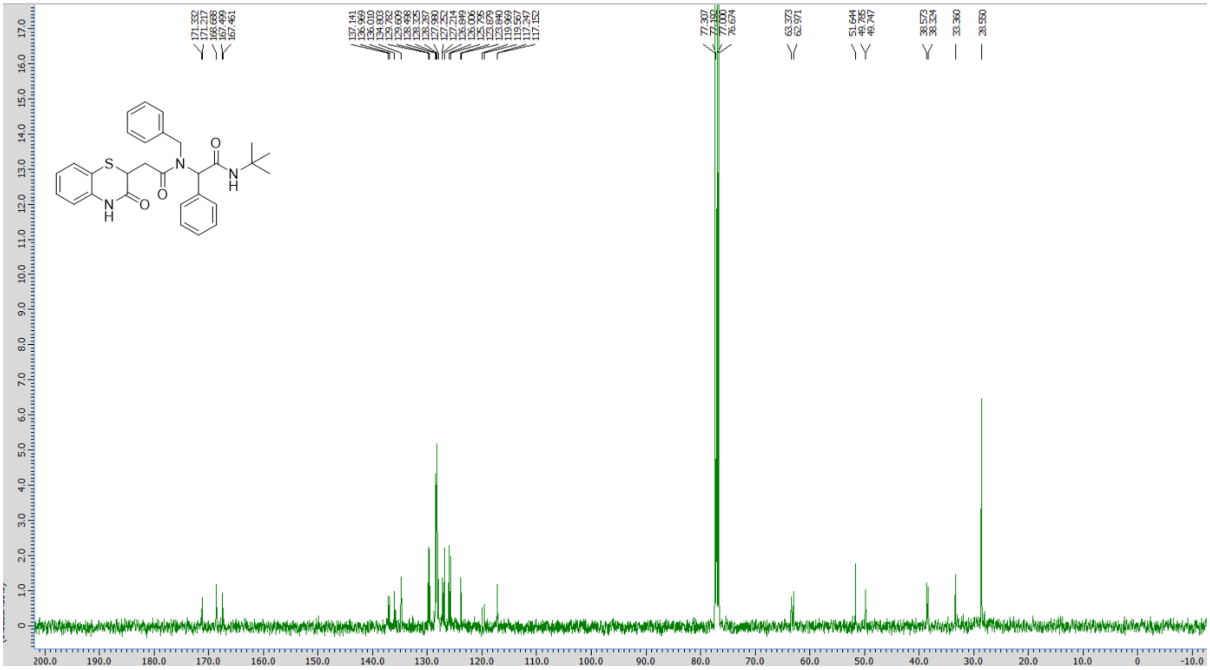

Figure S4: ^{13}C spectra of N-benzyl-N-(2-(tert-butylamino)-2-oxo-1-phenylethyl)-2-(3-oxo-3,4-dihydro-2H-benzo[b][1,4]-thiazin-2-yl)-acetamide (**8aA**)

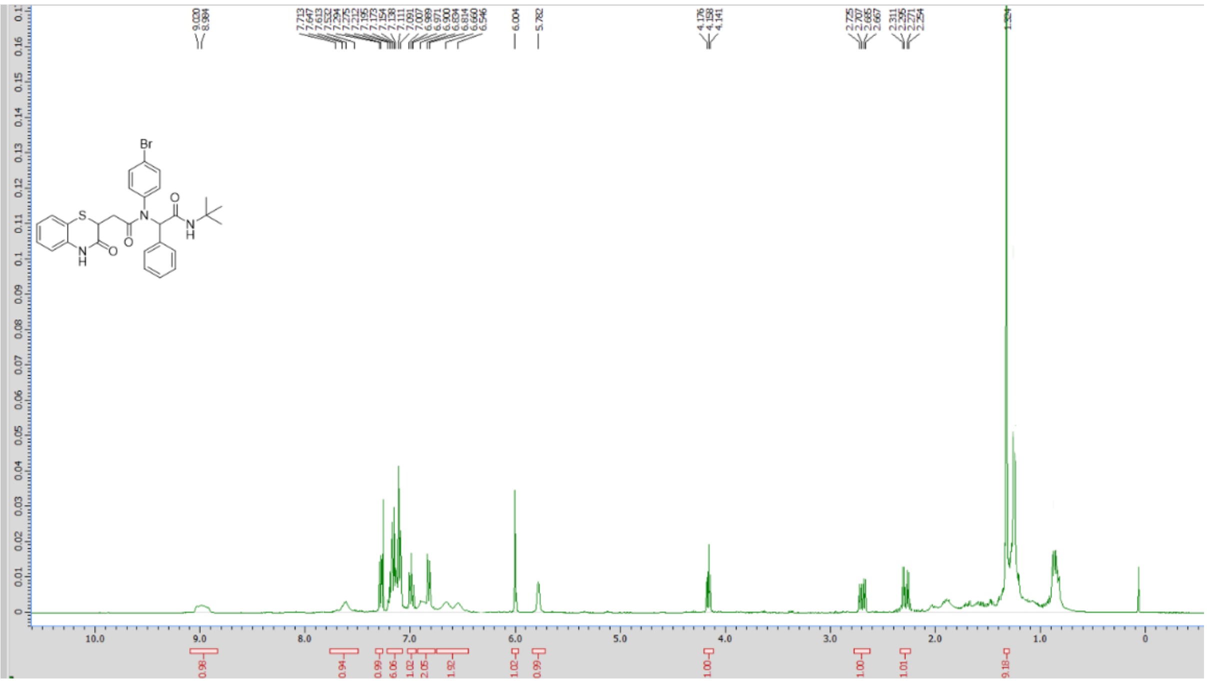

Figure S5: ^{1}H spectra N-(4-bromophenyl)-N-(2-(*tert*-butylamino)-2-oxo-1-phenylethyl)-2-(3-oxo-3,4-dihydro-2*H*-benzo[*b*][1,4]-thiazin-2-yl)-acetamide (**8aC**)

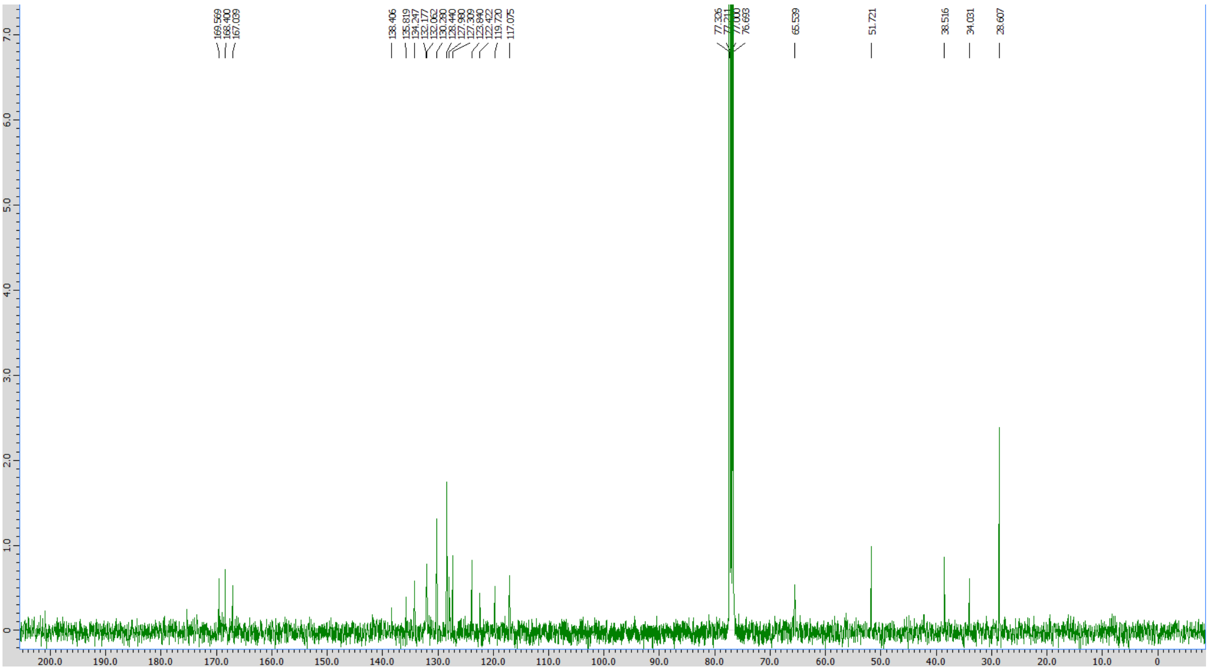

Figure S6: ^{13}C spectra of N-(4-bromophenyl)-N-(2-(*tert*-butylamino)-2-oxo-1-phenylethyl)-2-(3-oxo-3,4-dihydro-2*H*-benzo[b][1,4]-thiazin-2-yl)-acetamide (**8aC**)

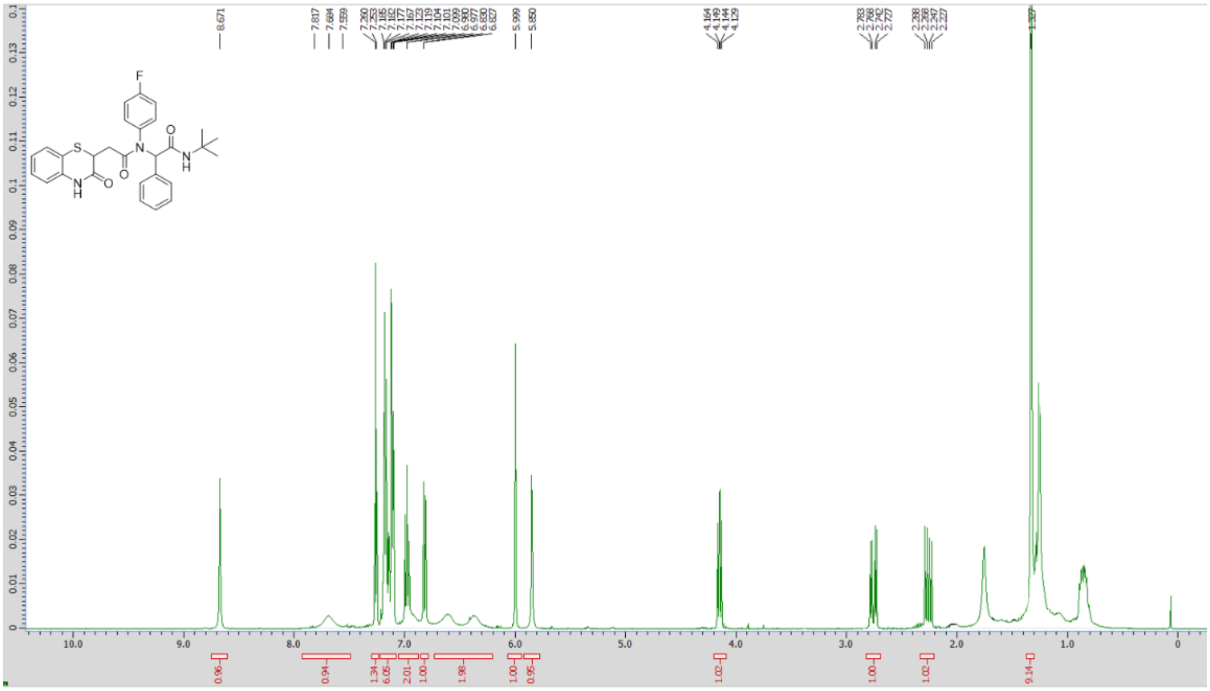

Figure S7: ^{1}H spectra of N-(*tert*-butyl)-2-(N-(4-fluorophenyl)-2-(3-oxo-3,4-dihydro-2*H*-benzo[*b*] [1,4]-thiazin-2-yl)-acetamido)-2-phenylacetamide (**8aD**)

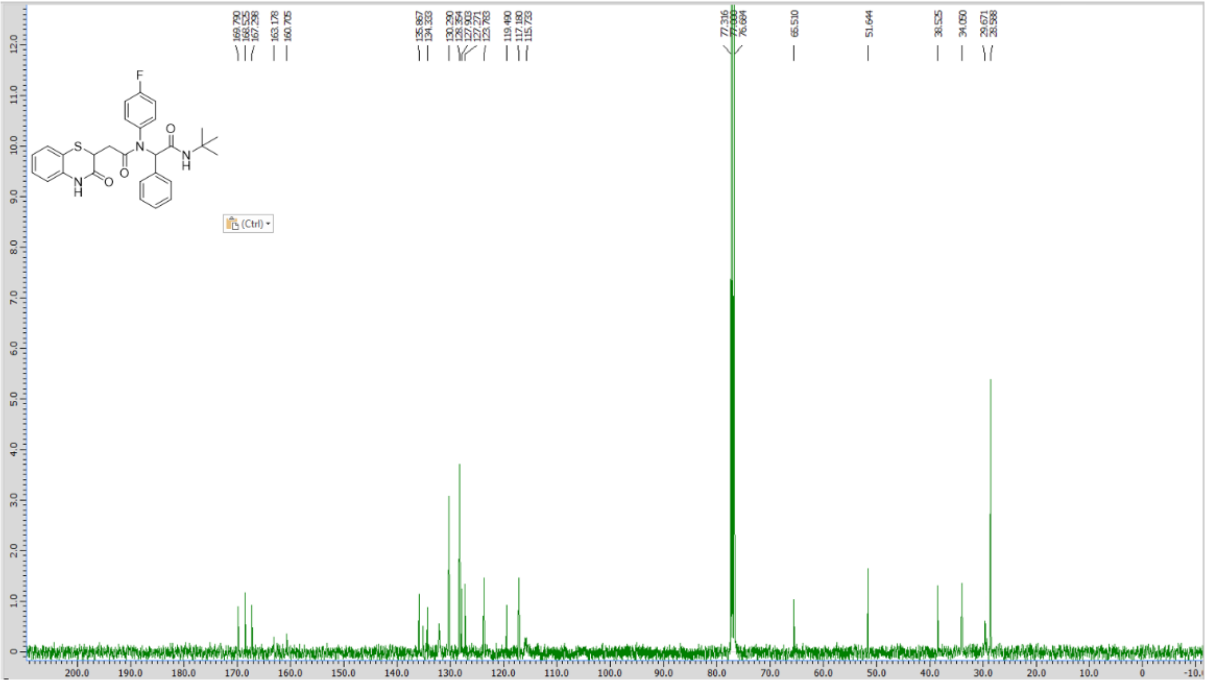

Figure S8: ^{13}C spectra of N-(*tert*-butyl)-2-(N-(4-fluorophenyl)-2-(3-oxo-3,4-dihydro-2*H*-benzo[*b*] [1,4]-thiazin-2-yl)-acetamido)-2-phenylacetamide (**8aD**)

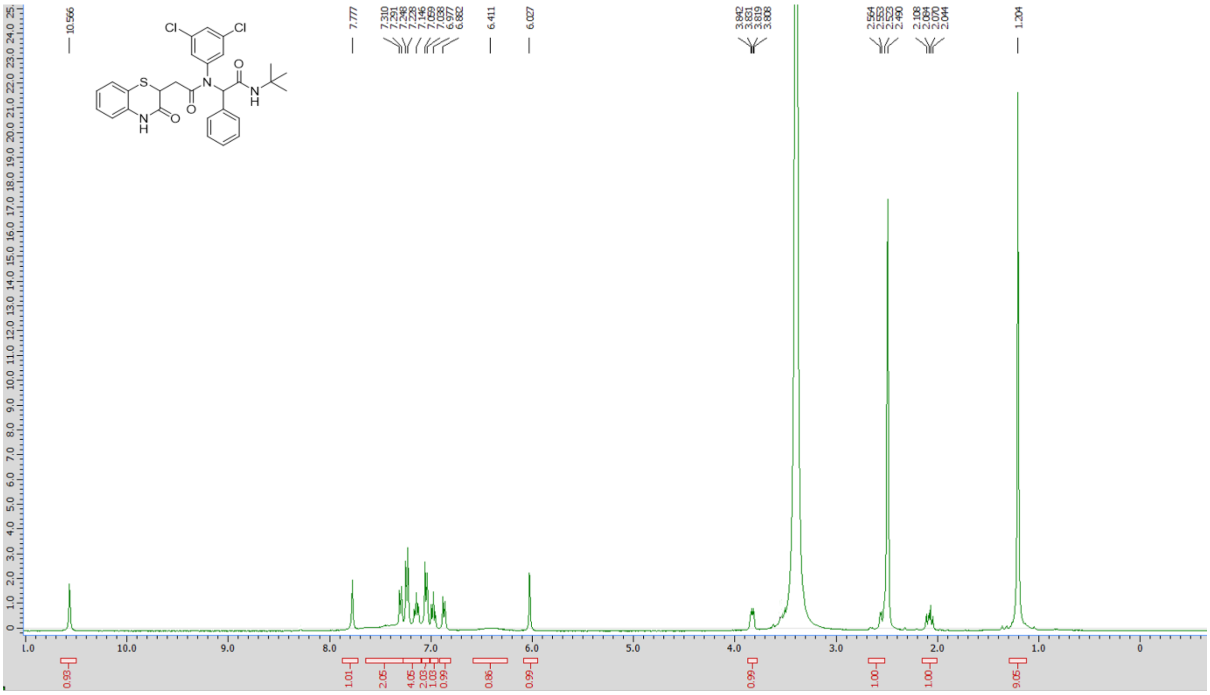

Figure S9: ^{1}H spectra of N-(*tert*-butyl)-2-(N-(3,5-dichlorophenyl)-2-(3-oxo-3,4-dihydro-2*H*-benzo[*b*][1,4]-thiazin-2-yl)-acetamido)-2-phenylacetamide (**8aJ**)

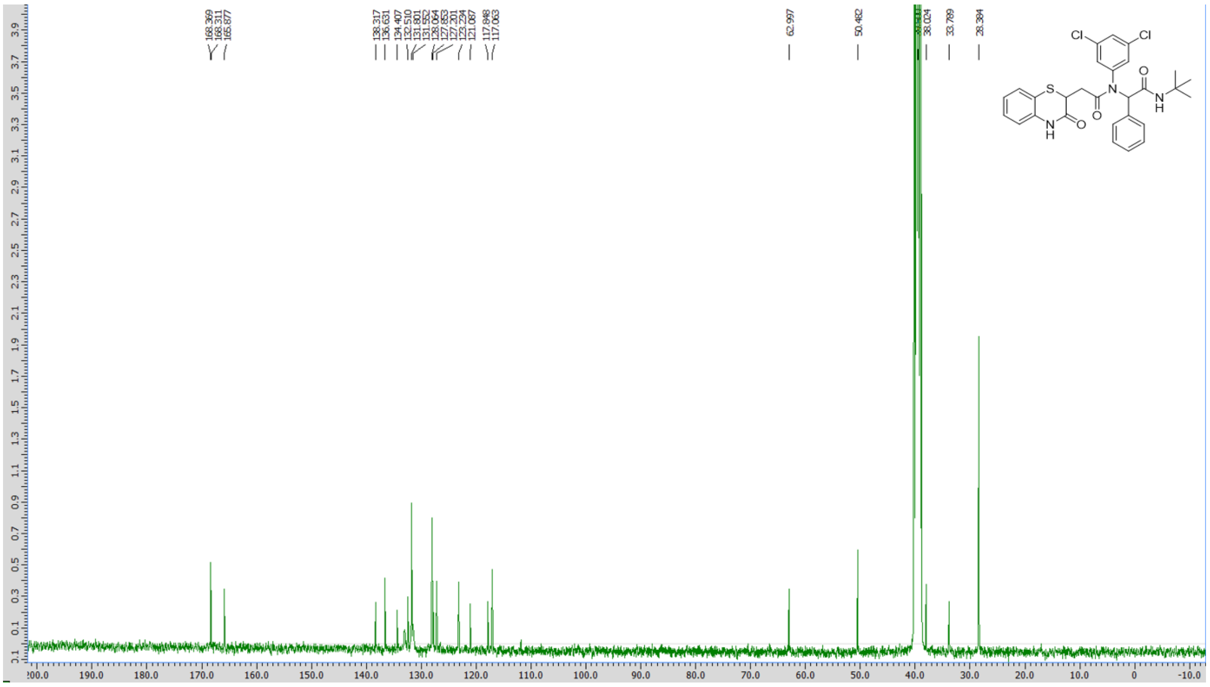

Figure S10: ^{13}C spectra of N-(*tert*-butyl)-2-(N-(3,5-dichlorophenyl)-2-(3-oxo-3,4-dihydro-2*H*-benzo[*b*][1,4]-thiazin-2-yl)-acetamido)-2-phenylacetamide (**8aJ**)

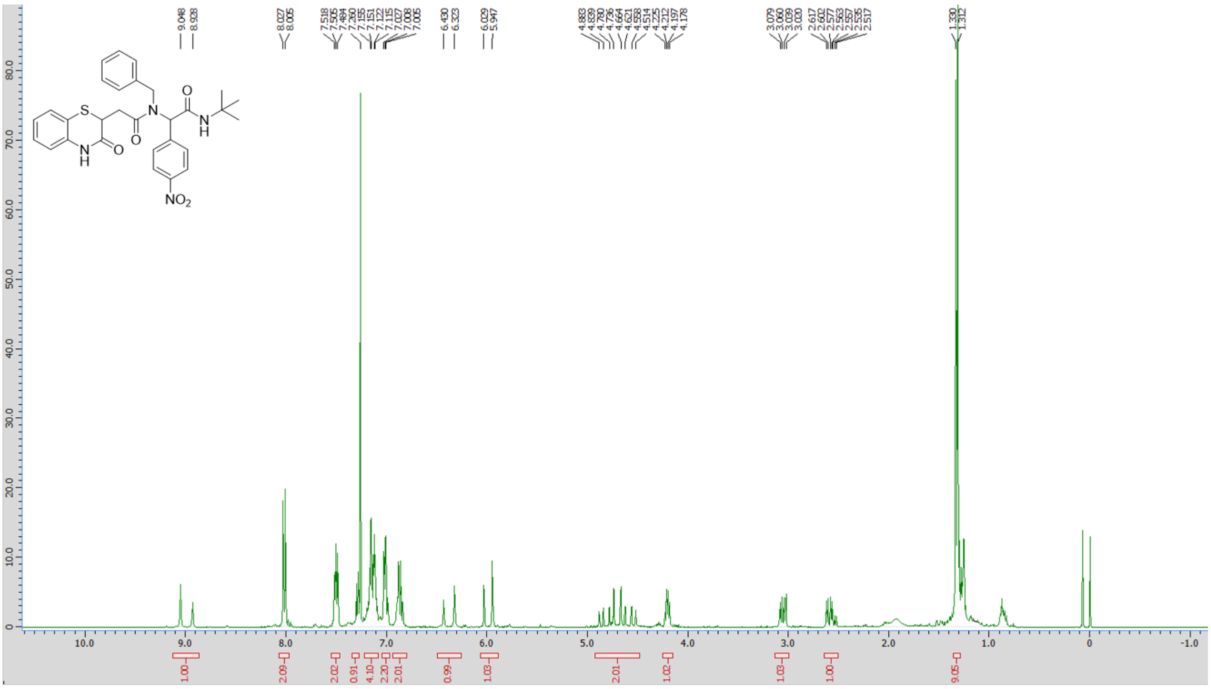

Figure S11: ^{1}H spectra of *N*-benzyl-*N*-(2-(*tert*-butylamino)-1-(4-nitrophenyl)-2-oxoethyl)-2-(3-oxo-3,4-dihydro-2*H*-benzo[*b*][1,4]-thiazin-2-yl)-acetamide (**8bA**)

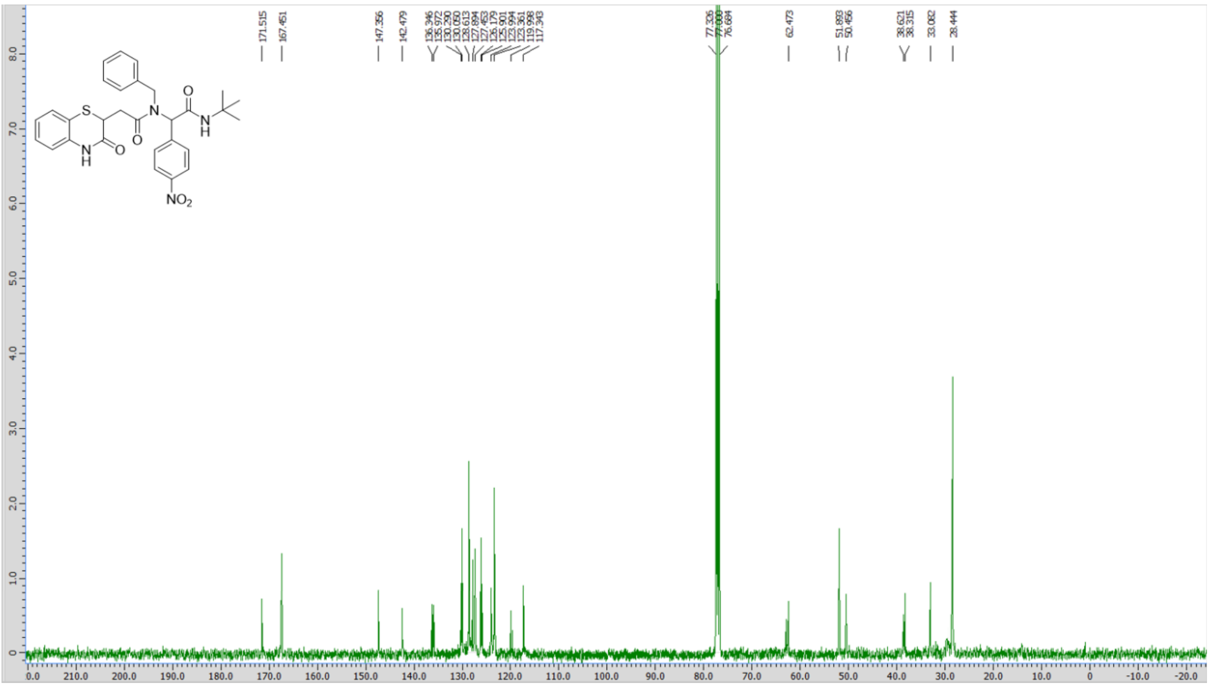

Figure S12: ^{13}C spectra of *N*-benzyl-*N*-(2-(*tert*-butylamino)-1-(4-nitrophenyl)-2-oxoethyl)-2-(3-oxo-3,4-dihydro-2*H*-benzo[*b*][1,4]-thiazin-2-yl)-acetamide (**8bA**)

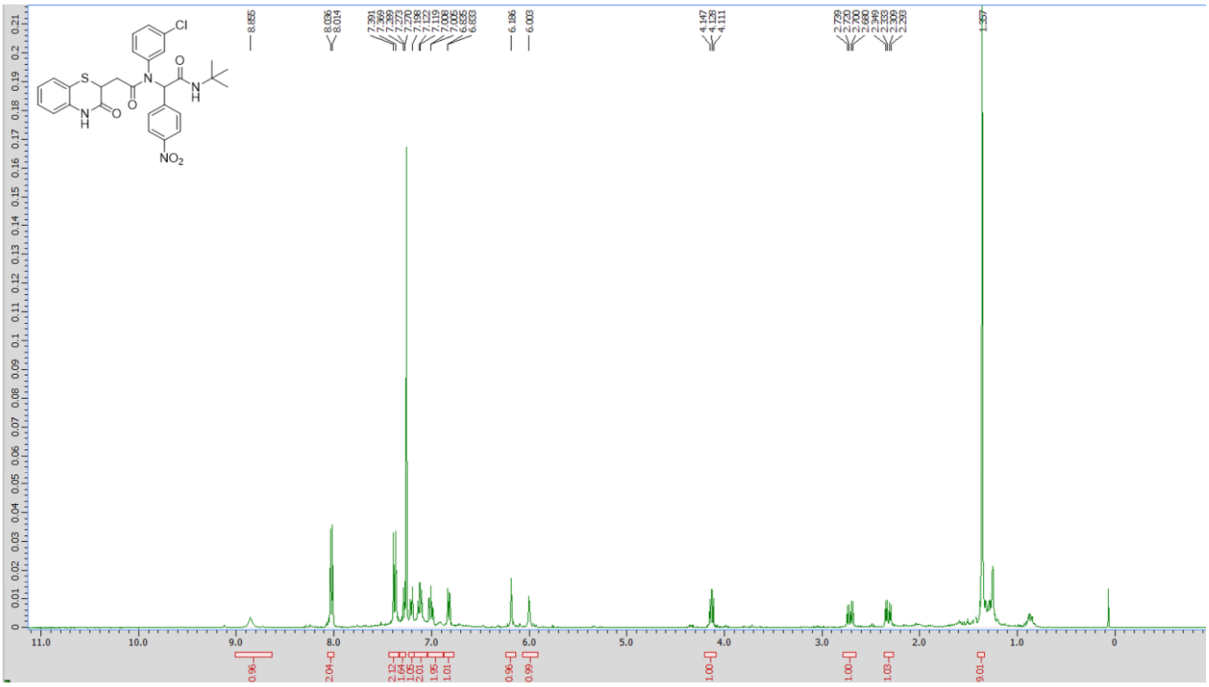

Figure S13: ^{1}H spectra of *N*-(*tert*-butyl)-2-(*N*-(3-chlorophenyl)-2-(3-oxo-3,4-dihydro-2*H*-benzo[*b*] [1,4]-thiazin-2-yl)-acetamido)-2-(4-nitrophenyl)-acetamide (**8bB**)

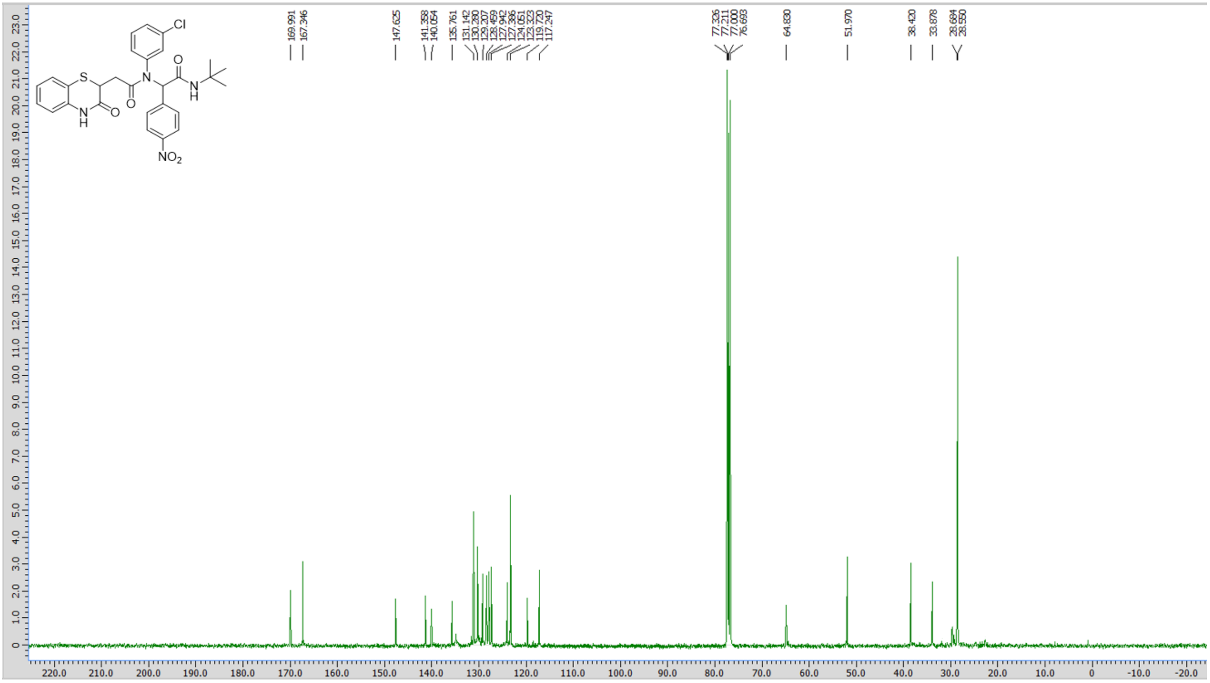

Figure S14: ^{13}C spectra of *N*-(*tert*-butyl)-2-(*N*-(3-chlorophenyl)-2-(3-oxo-3,4-dihydro-2*H*-benzo[*b*] [1,4]-thiazin-2-yl)-acetamido)-2-(4-nitrophenyl)-acetamide (**8bB**)

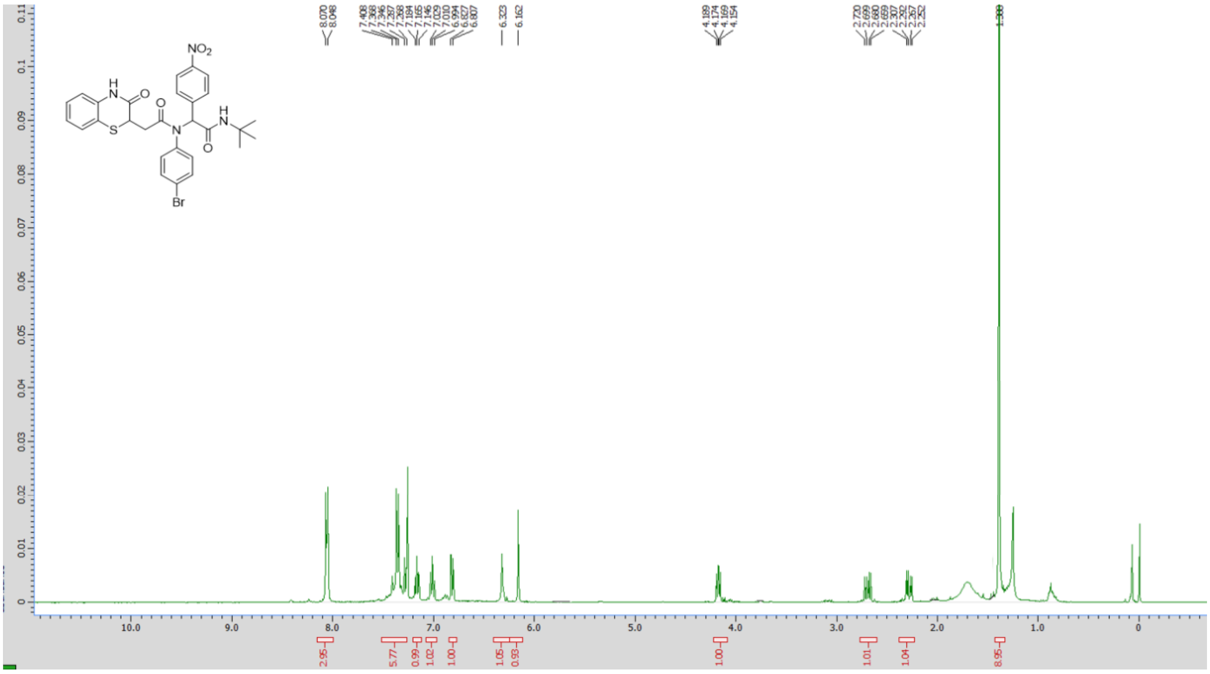

Figure S5: ^{1}H spectra of N-(4-bromophenyl)-*N*-(2-(*tert*-butylamino)-1-(4-nitrophenyl)-2-oxoethyl)-2-(3-oxo-3,4-dihydro-2*H*-benzo[*b*][1,4]-thiazin-2-yl)-acetamide (**8bC**)

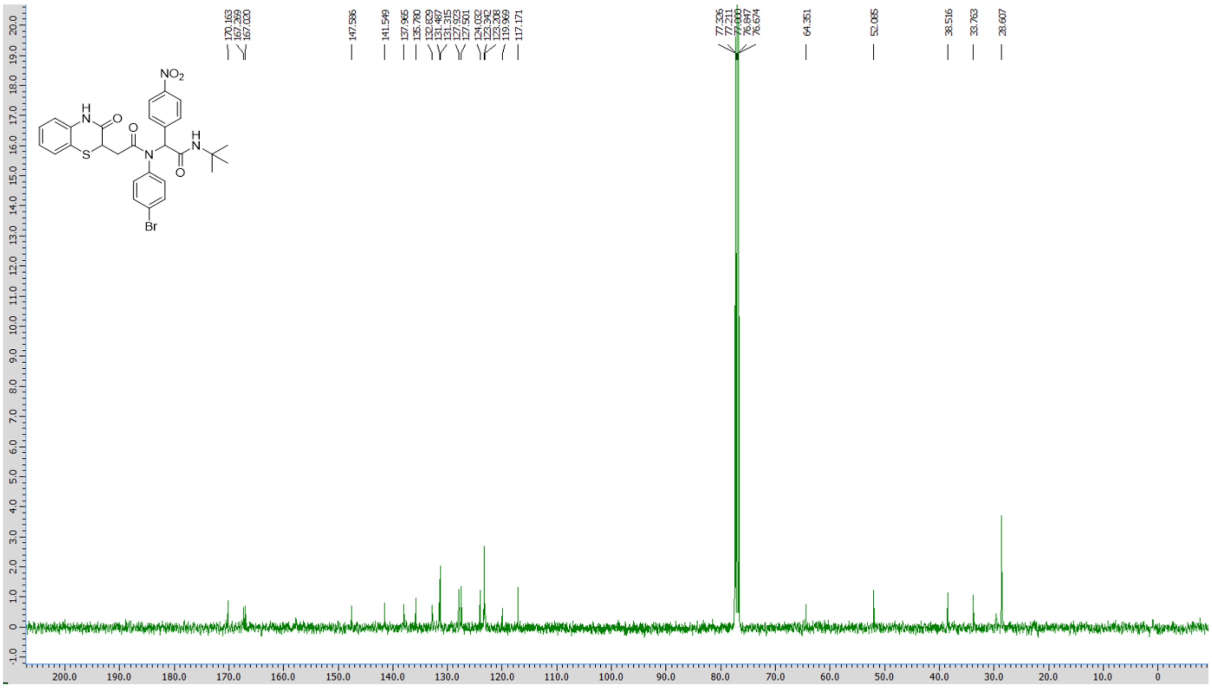

Figure S6:^{13}C spectra of *N*-(4-bromophenyl)-*N*-(2-(*tert*-butylamino)-1-(4-nitrophenyl)-2-oxoethyl)-2-(3-oxo-3,4-dihydro-2*H*-benzo[*b*][1,4]-thiazin-2-yl)-acetamide (**8bC**)

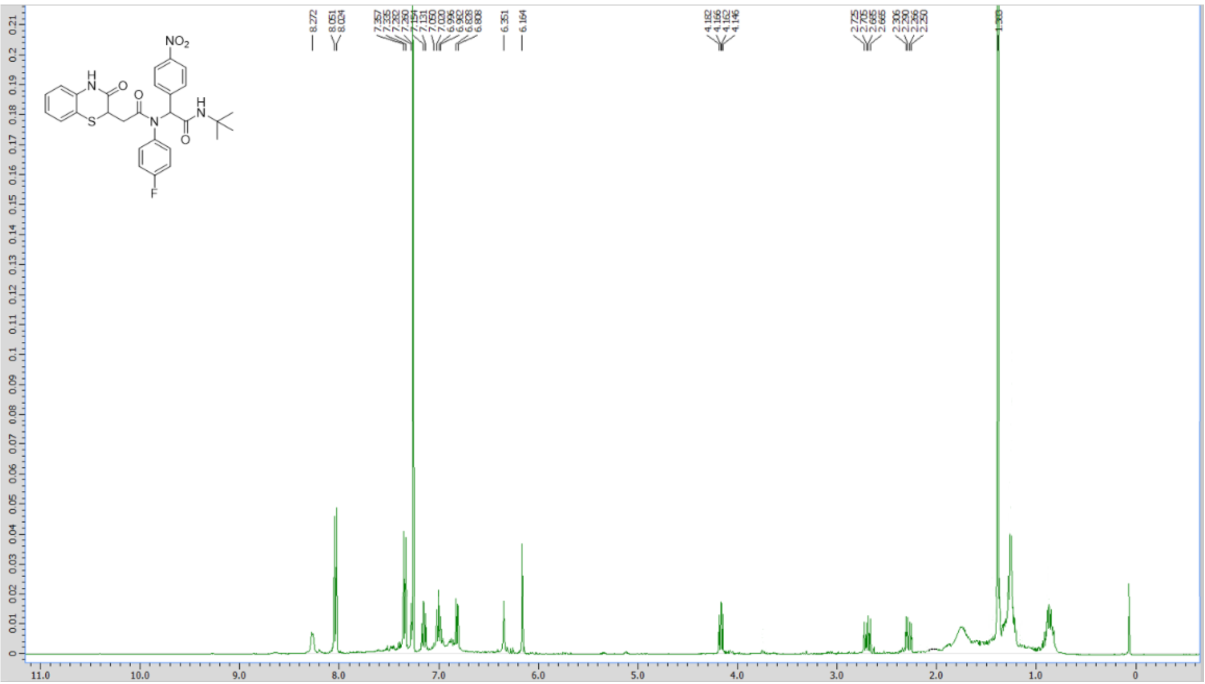

Figure S17: ^{1}H spectra of *N*-(*tert*-butyl)-2-(*N*-(4-fluorophenyl)-2-(3-oxo-3,4-dihydro-2*H*-benzo[*b*] [1,4]-thiazin-2-yl)-acetamido)-2-(4-nitrophenyl)-acetamide (**8bD**)

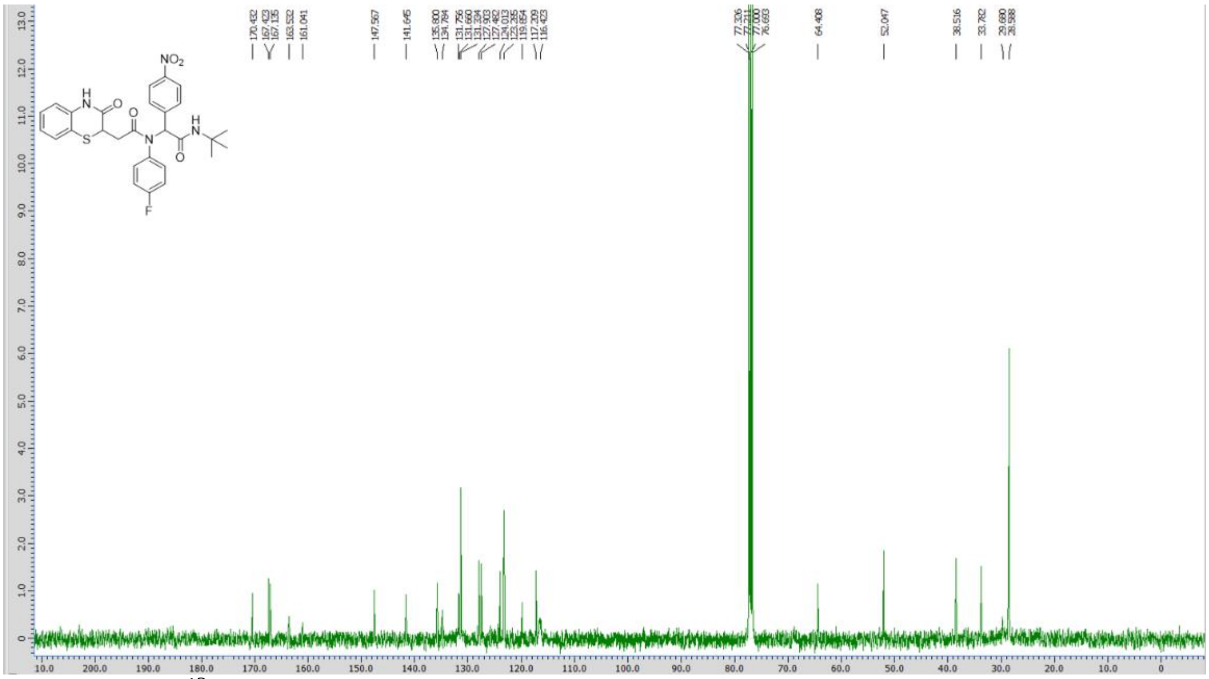

Figure S18: ^{13}C spectra of *N*-(*tert*-butyl)-2-(*N*-(4-fluorophenyl)-2-(3-oxo-3,4-dihydro-2*H*-benzo[*b*] [1,4]-thiazin-2-yl)-acetamido)-2-(4-nitrophenyl)-acetamide (**8bD**)

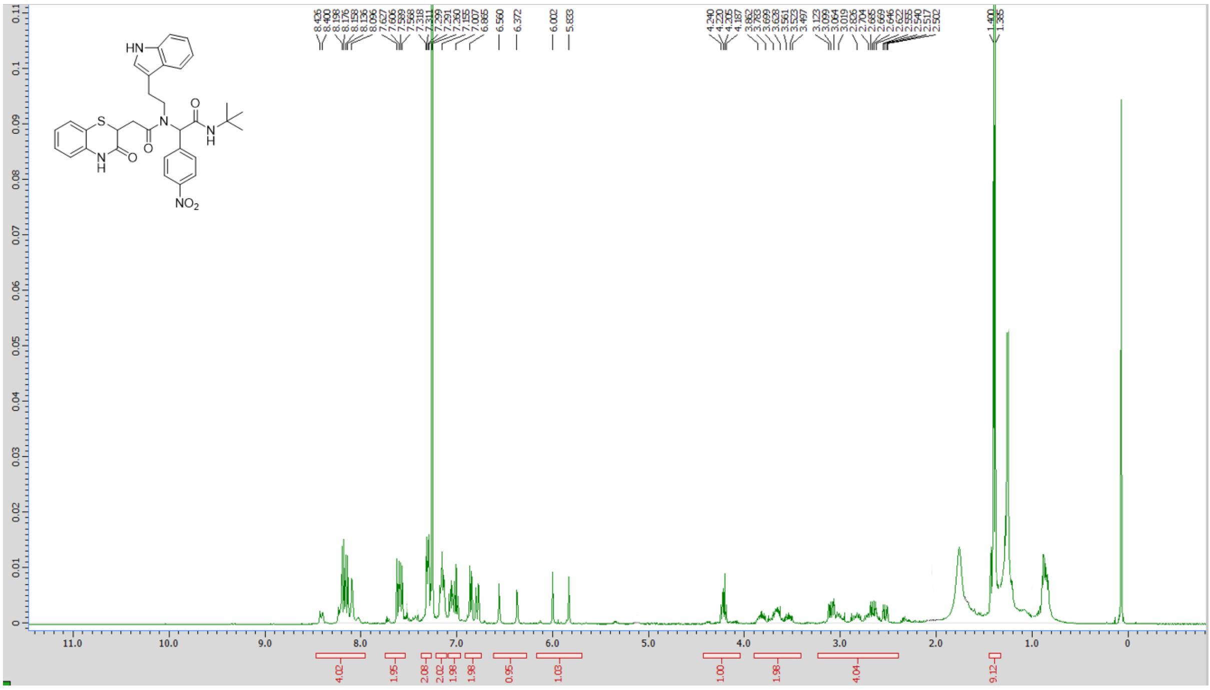

Figure S19: ^{1}H spectra of *N*-(2-(1*H*-indol-3-yl)-ethyl)-*N*-(2-(*tert*-butylamino)-1-(4-nitrophenyl)-2-oxoethyl)-2-(3-oxo-3,4-dihydro-2*H*-benzo[*b*][1,4]-thiazin-2-yl)-acetamide (**8bE**)

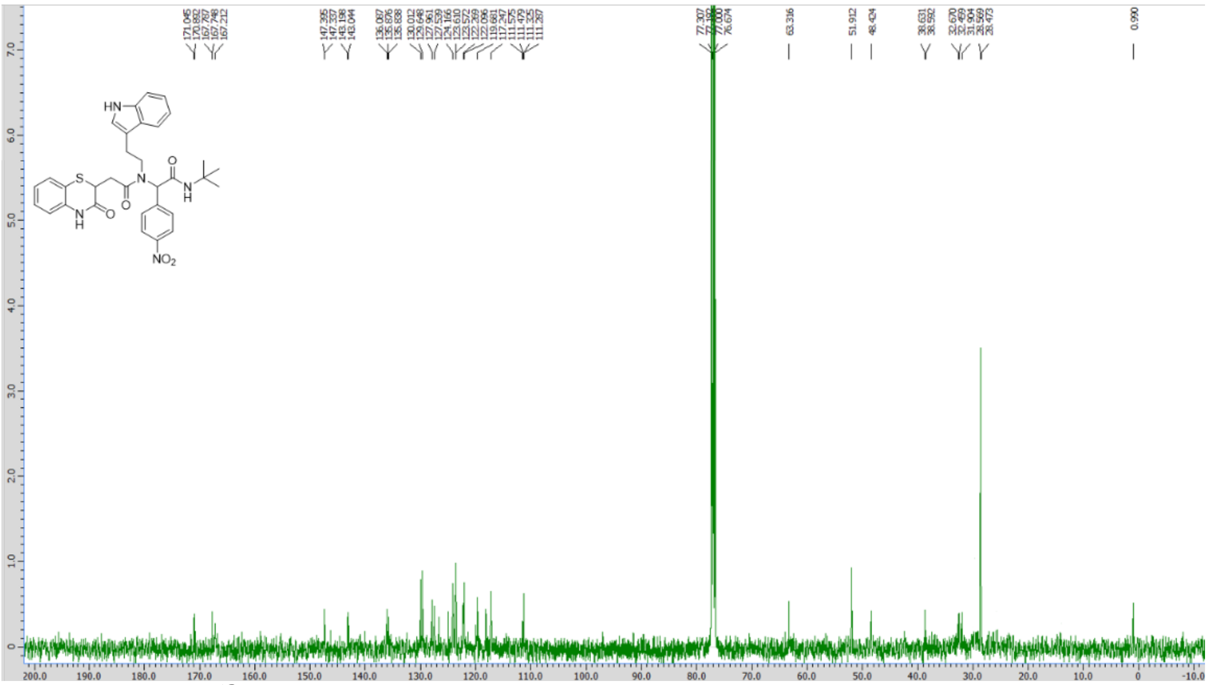

Figure S20: ^{13}C spectra of *N*-(2-(1*H*-indol-3-yl)-ethyl)-*N*-(2-(*tert*-butylamino)-1-(4-nitrophenyl)-2-oxoethyl)-2-(3-oxo-3,4-dihydro-2*H*-benzo[*b*][1,4]-thiazin-2-yl)-acetamide (**8bE**)

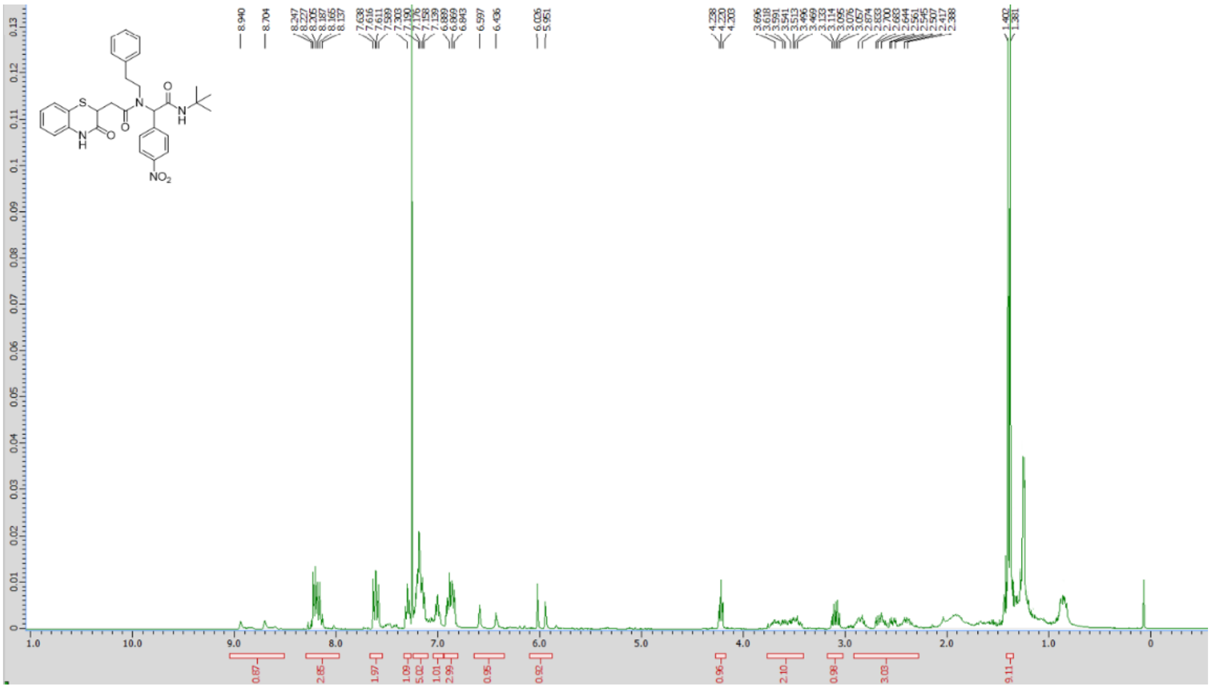

Figure S21: ^{1}H spectra of N-(*tert*-butyl)-2-(4-nitrophenyl)-2-(2-(3-oxo-3,4-dihydro-2*H*-benzo[*b*][1,4]-thiazin-2-yl)-N-phenethylacetamido)-acetamide (**8bF**)

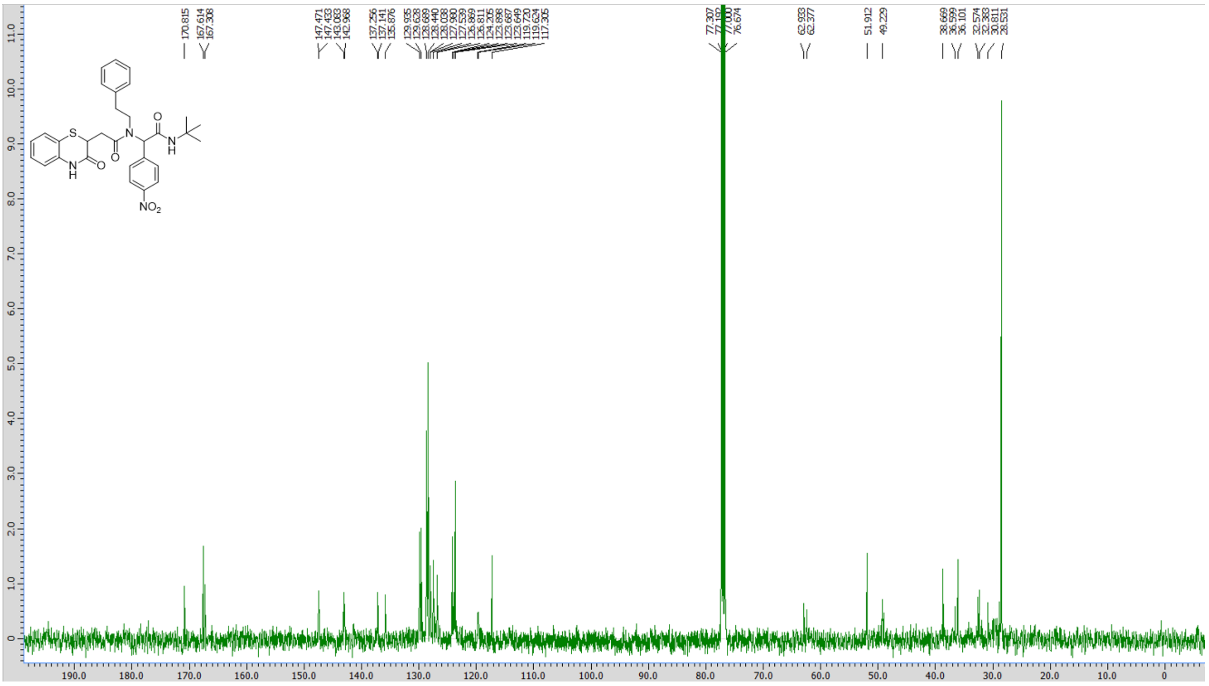

Figure S22: ^{13}C spectra of *N*-(*tert*-butyl)-2-(4-nitrophenyl)-2-(2-(3-oxo-3,4-dihydro-2*H*-benzo[*b*][1,4]-thiazin-2-yl)-*N*-phenethyl-acetamido)-acetamide (**8bF**)

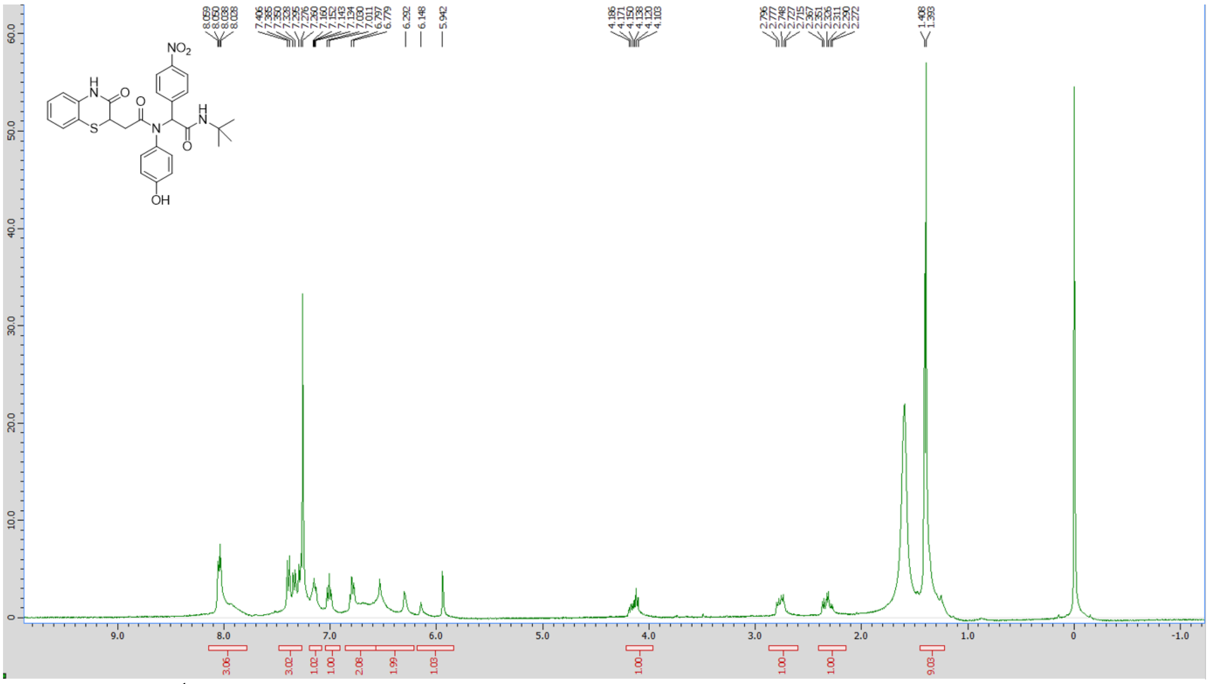

Figure S23: ^{1}H spectra of *N*-(tert-butyl)-2-(*N*-(4-hydroxyphenyl)-2-(3-oxo-3,4-dihydro-*2H*-benzo[*b*] [1,4]-thiazin-2-yl)-acetamido)-2-(4-nitrophenyl)-acetamide (**8bI**)

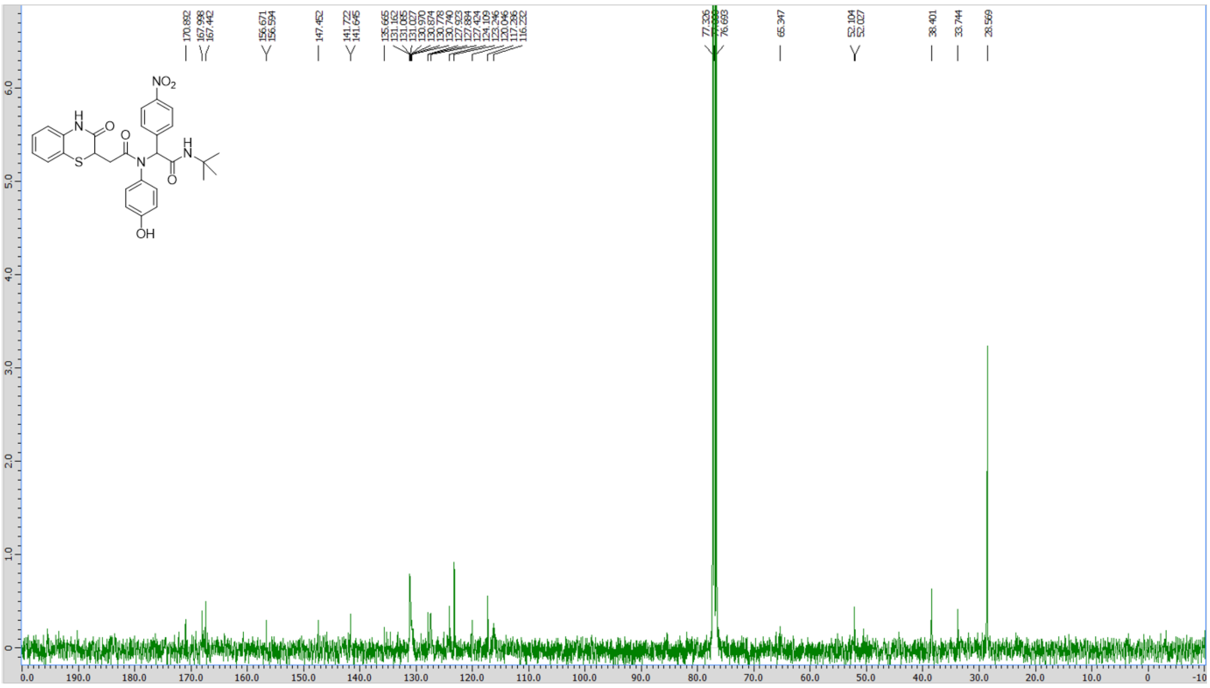

Figure S24: ^{13}C spectra of *N*-(*tert*-butyl)-2-(*N*-(4-hydroxyphenyl)-2-(3-oxo-3,4-dihydro-2*H*-benzo[*b*] [1,4]-thiazin-2-yl)-acetamido)-2-(4-nitrophenyl)-acetamide (**8bI**)

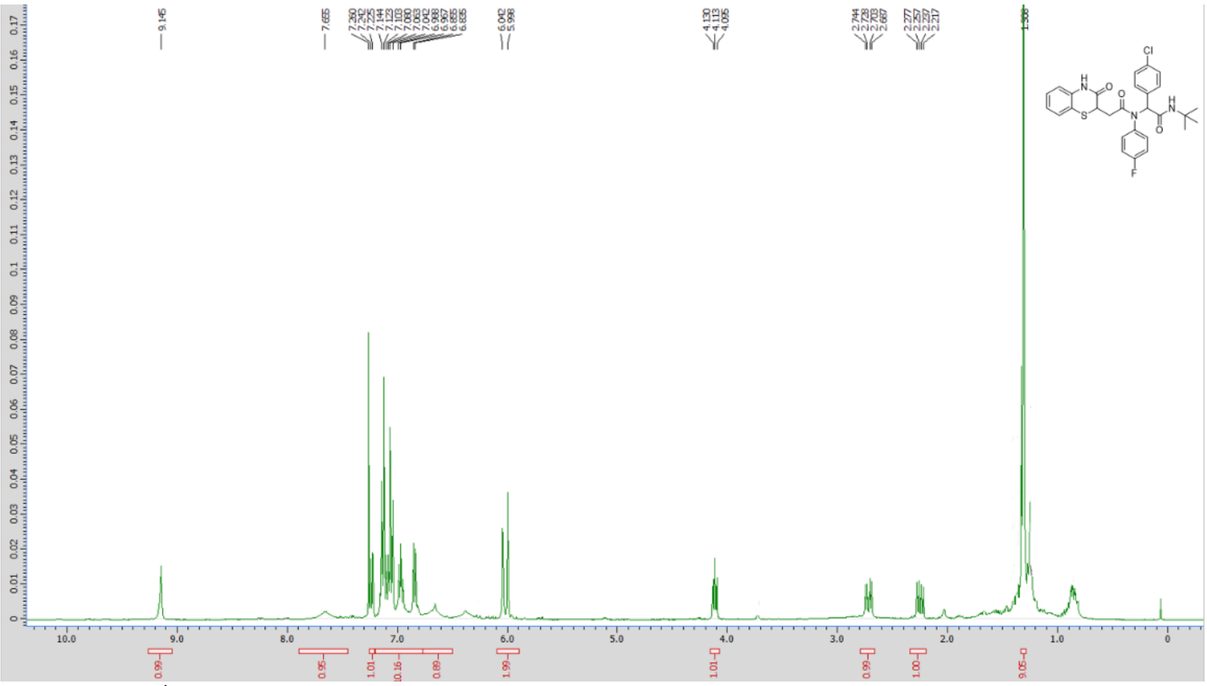

Figure S7: ^{1}H spectra of *N*-(*tert*-butyl)-2-(4-chlorophenyl)-2-(*N*-(4-fluorophenyl)-2-(3-oxo-3,4-dihydro-2*H*-benzo[*b*][1,4]-thiazin-2-yl)-acetamido)-acetamide (**8cC**)

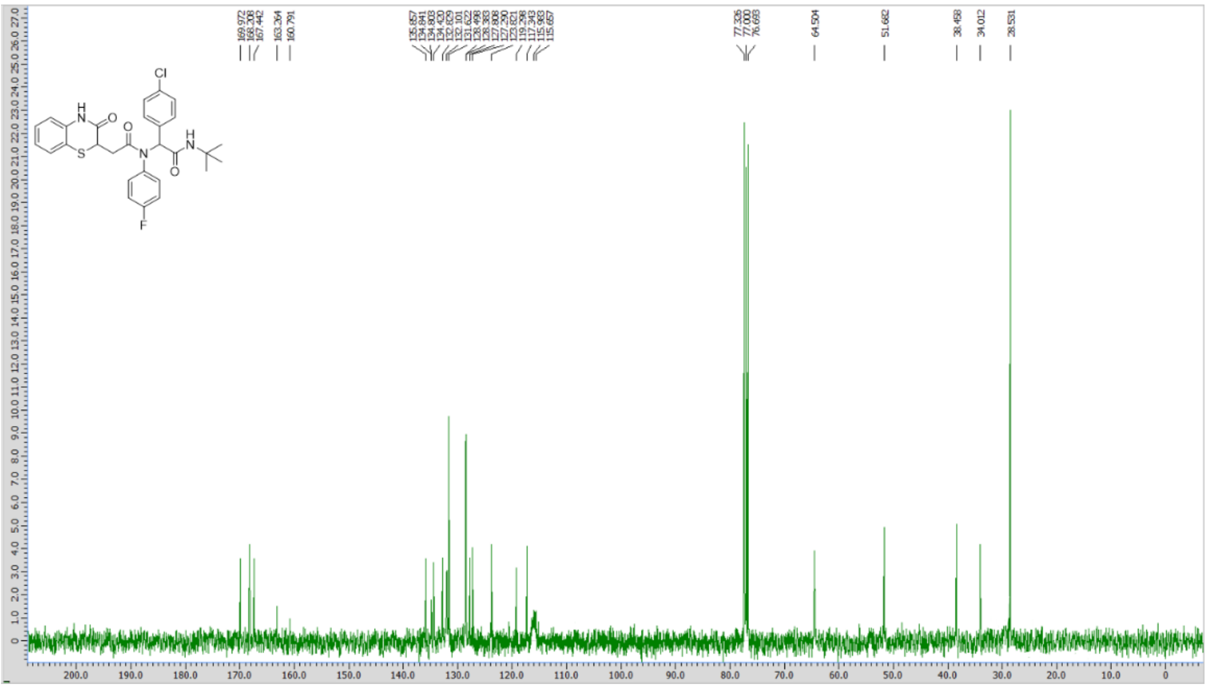

Figure S8: ^{13}C spectra of *N*-(*tert*-butyl)-2-(4-chlorophenyl)-2-(*N*-(4-fluorophenyl)-2-(3-oxo-3,4-dihydro-2*H*-benzo[*b*][1,4]-thiazin-2-yl)-acetamido)-acetamide (**8cC**)

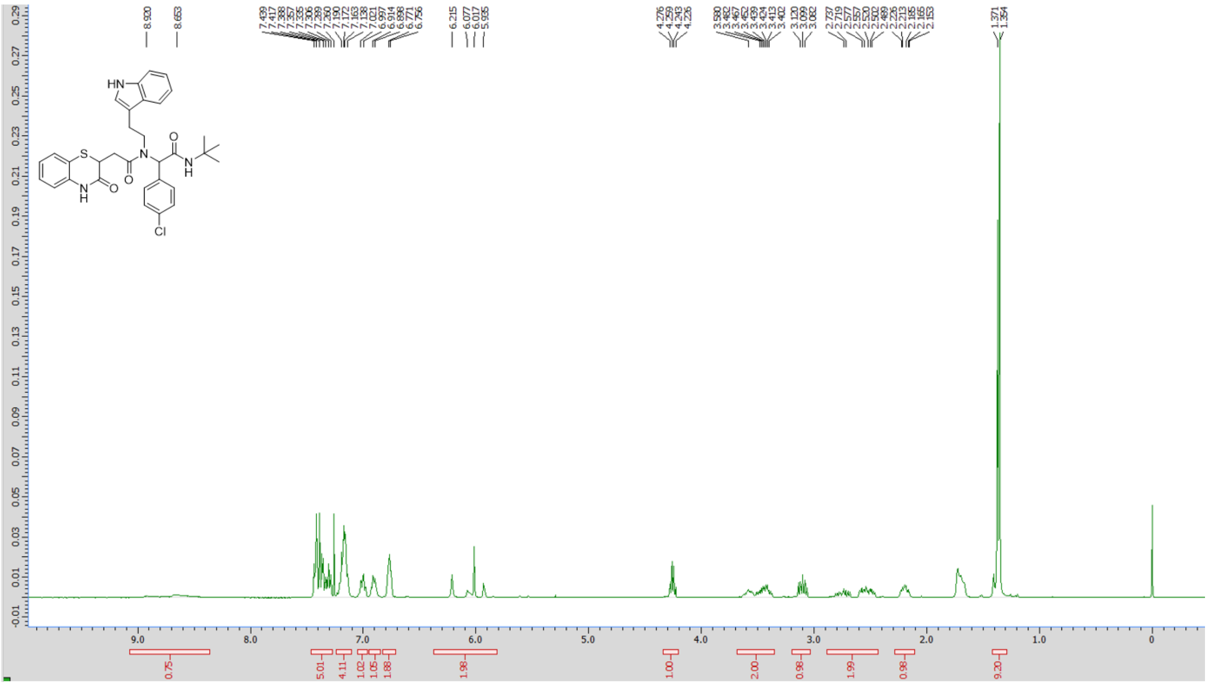

Figure S27: ^{1}H spectra of *N*-(2-(1*H*-indol-3-yl)-ethyl)-*N*-(2-(*tert*-butylamino)-1-(4-chlorophenyl)-2-oxoethyl)-2-(3-oxo-3,4-dihydro-2*H*-benzo[*b*][1,4]-thiazin-2-yl)-acetamide (**8cE**)

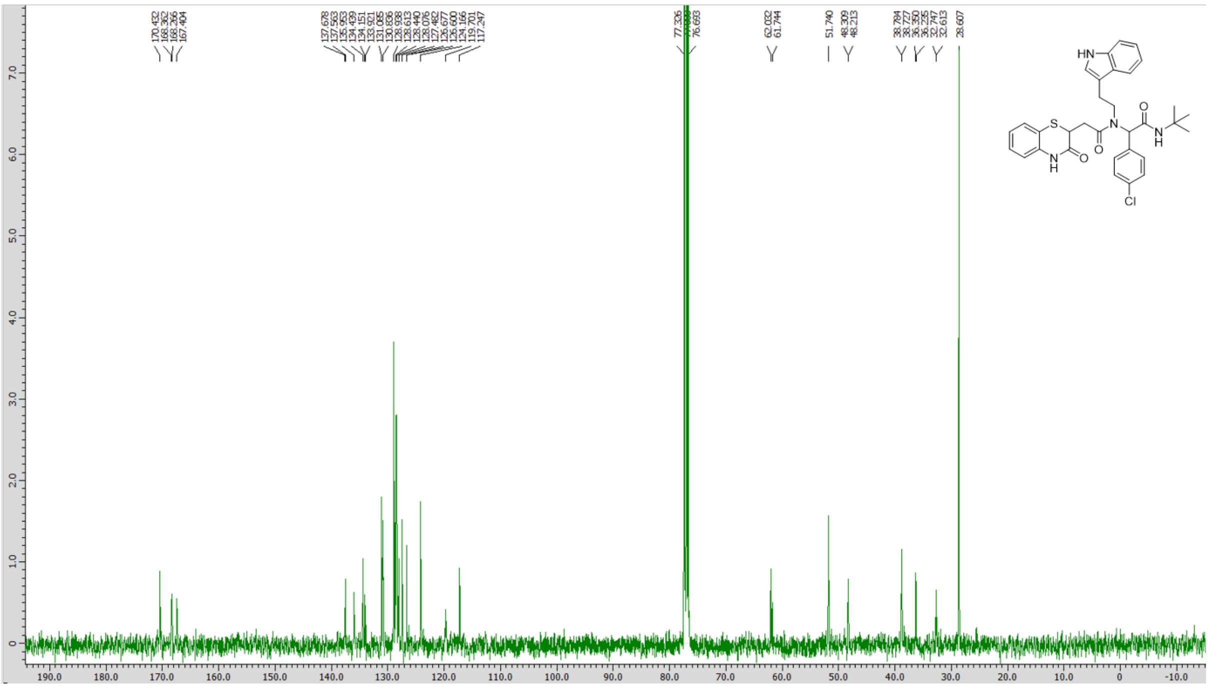

Figure S28: ^{13}C spectra of *N*-(2-(1*H*-indol-3-yl)-ethyl)-*N*-(2-(*tert*-butylamino)-1-(4-chlorophenyl)-2-oxoethyl)-2-(3-oxo-3,4-dihydro-2*H*-benzo[*b*][1,4]-thiazin-2-yl)-acetamide (**8cE**)

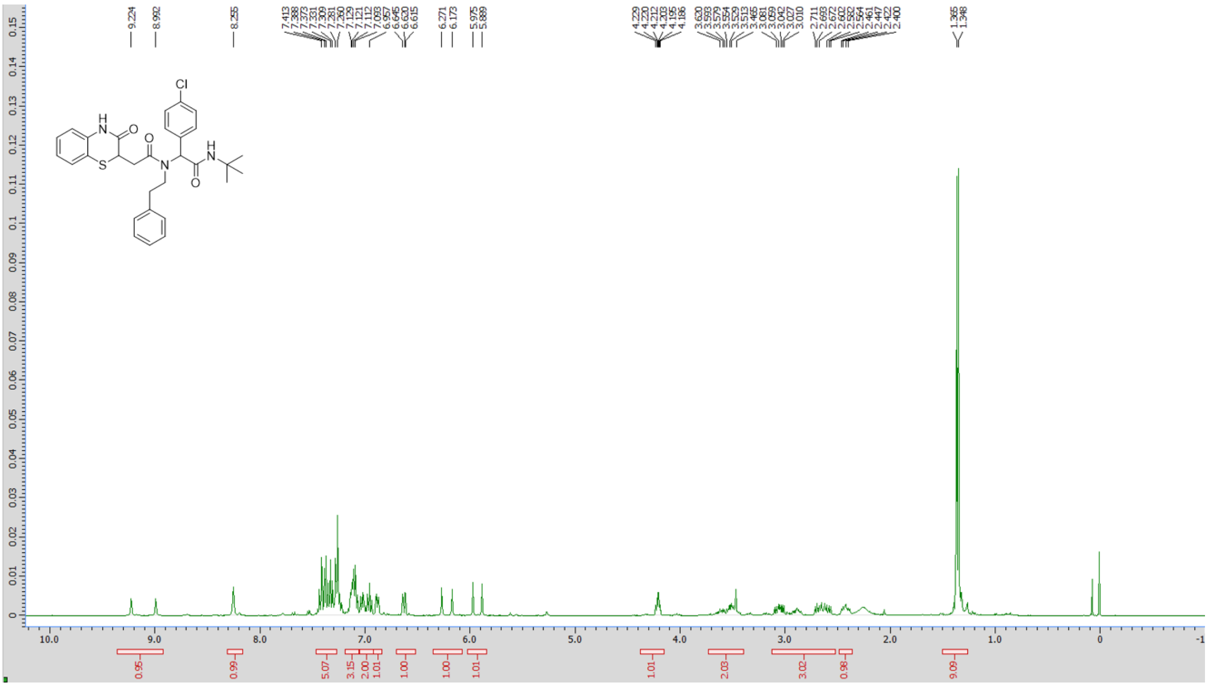

Figure S29: ¹H spectra of *N*-(*tert*-butyl)-2-(4-chlorophenyl)-2-(2-(3-oxo-3,4-dihydro-2*H*-benzo[*b*] [1,4]-thiazin-2-yl)-*N*-phenethyl-acetamido)-acetamide (**8cF**)

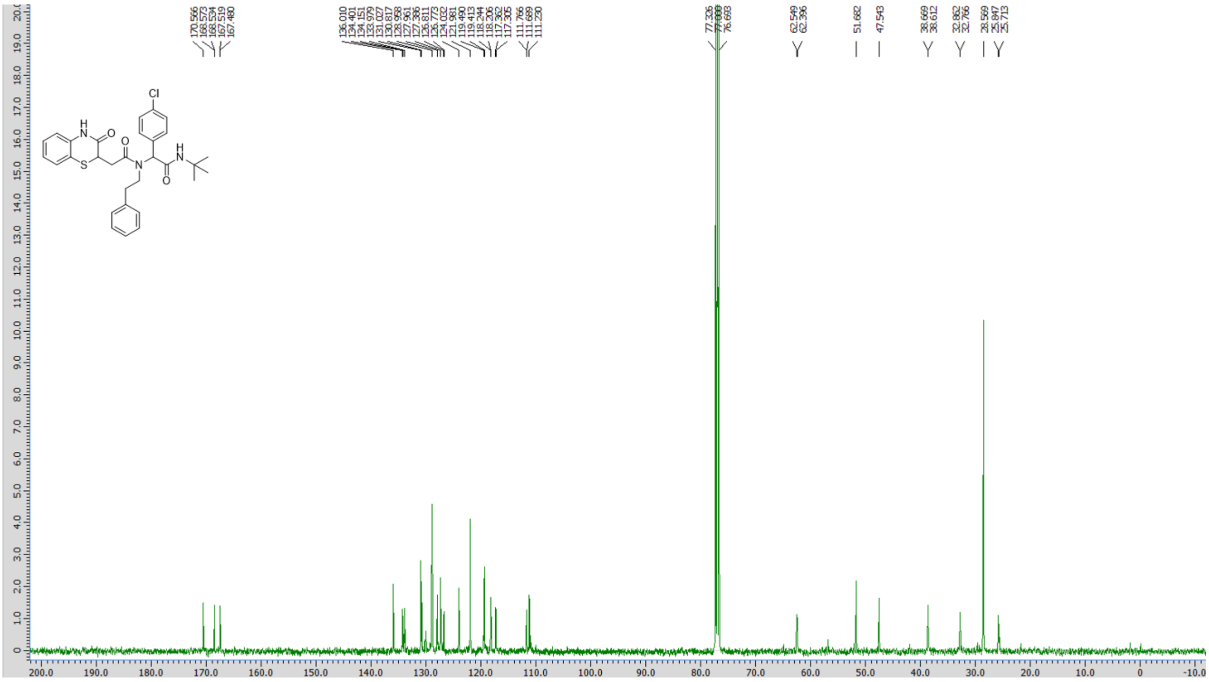

Figure S30: ¹³C spectra of *N*-(*tert*-butyl)-2-(4-chlorophenyl)-2-(2-(3-oxo-3,4-dihydro-2*H*-benzo[*b*] [1,4]-thiazin-2-yl)-*N*-phenethyl-acetamido)-acetamide (**8cF**)

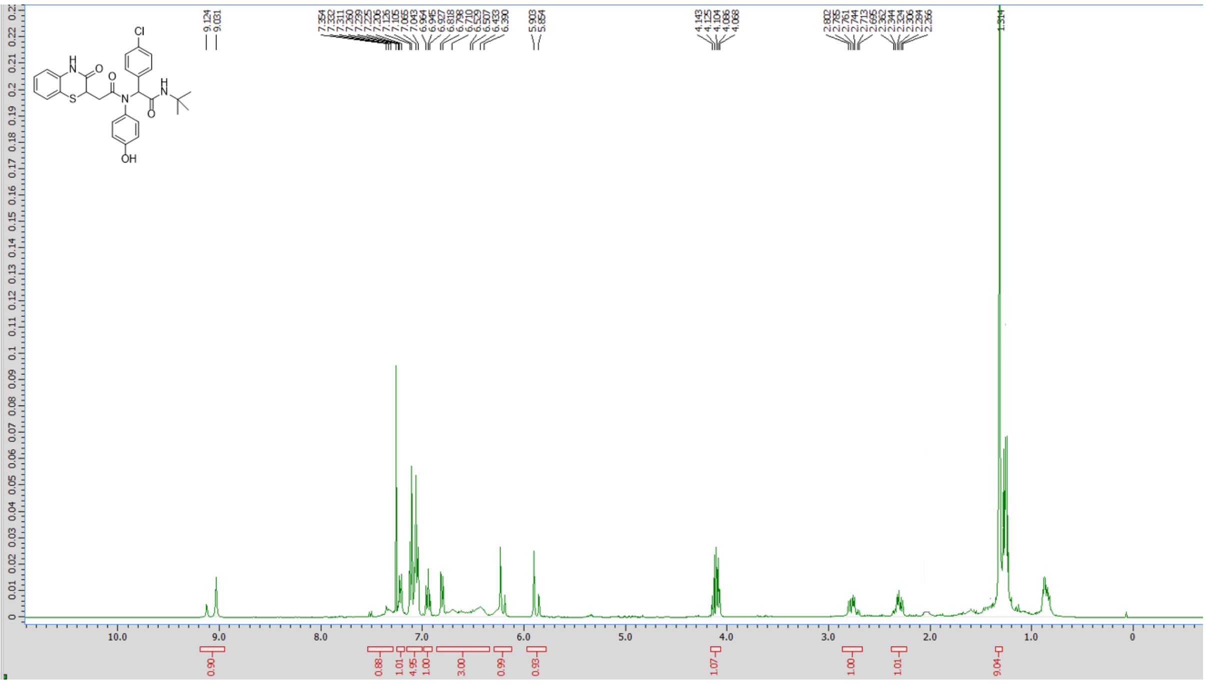

Figure S31: ^{1}H spectra of *N*-(*tert*-butyl)-2-(4-chlorophenyl)-2-(*N*-(4-hydroxyphenyl)-2-(3-oxo-3,4-dihydro-2*H*-benzo[*b*][1,4]-thiazin-2-yl)-acetamido)-acetamide (**8cI**)

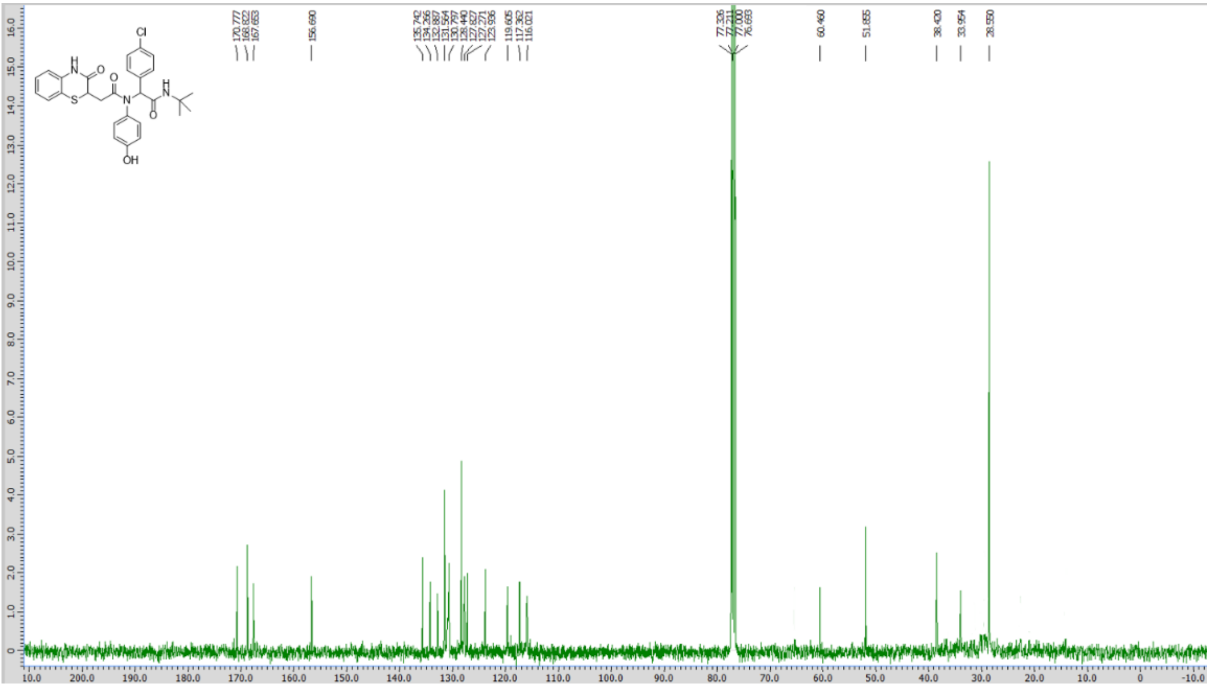

Figure S32: ^{13}C spectra of *N*-(*tert*-butyl)-2-(4-chlorophenyl)-2-(*N*-(4-hydroxyphenyl)-2-(3-oxo-3,4-dihydro-2*H*-benzo[*b*][1,4]-thiazin-2-yl)-acetamido)-acetamide (**8cI**)

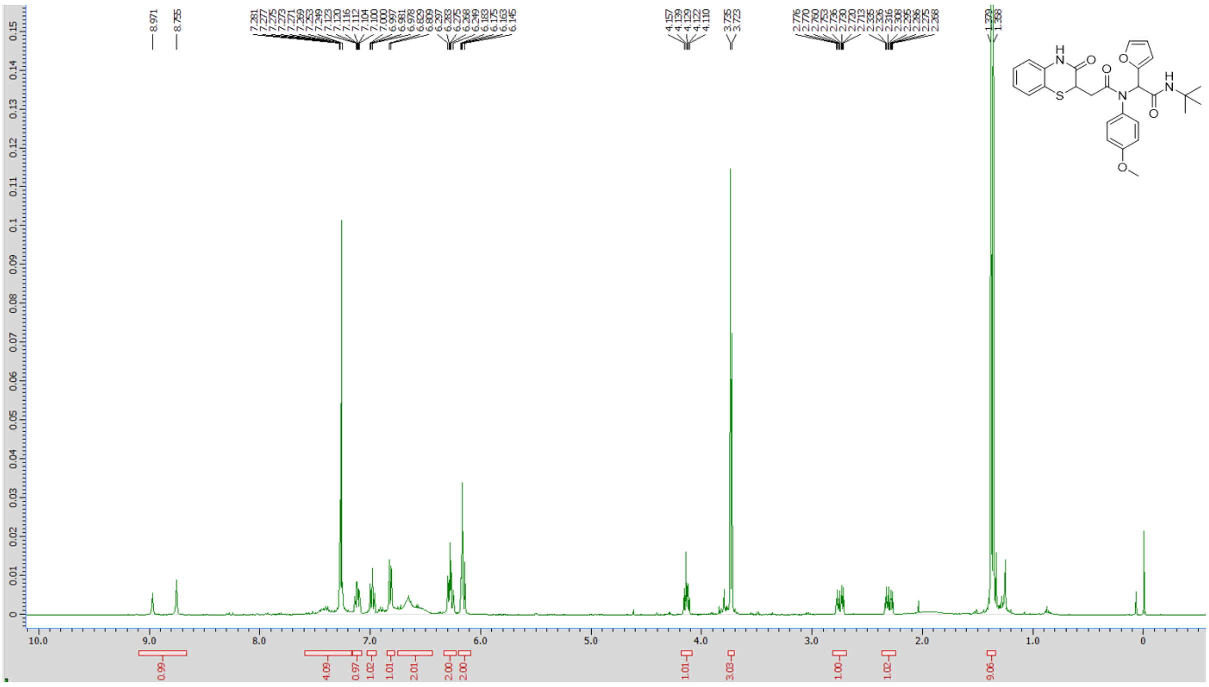

Figure S33: ^{1}H spectra of *N*-(*tert*butyl)-2-(furan-2-yl)-2-(*N*-(4-methoxyphenyl)-2-(3-oxo-3,4-dihydro-2*H*-benzo[*b*][1,4]-thiazin-2-yl)-acetamido)-acetamide (**8dG**)

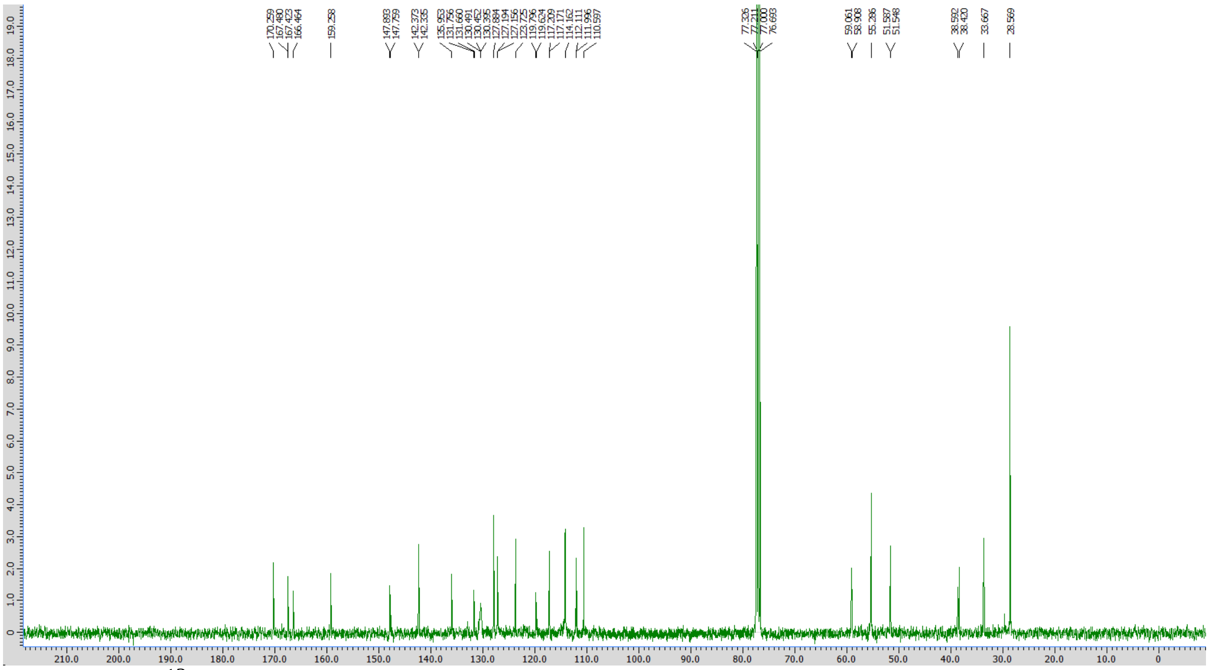

Figure S34: ^{13}C spectra of *N*-(*tert*butyl)-2-(furan-2-yl)-2-(*N*-(4-methoxyphenyl)-2-(3-oxo-3,4-dihydro-2*H*-benzo[*b*][1,4]-thiazin-2-yl)-acetamido)-acetamide (**8dG**)

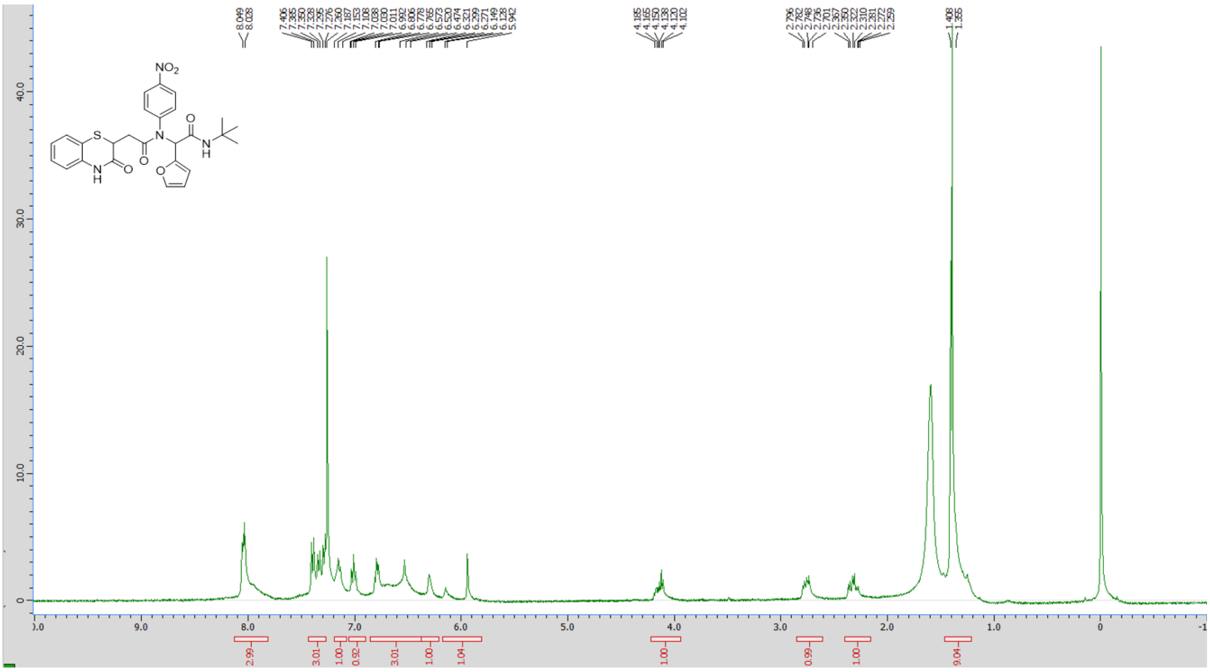

Figure S35: ^{1}H spectra of *N*-(*tert*-butyl)-2-(furan-2-yl)-2-(*N*-(4-nitrophenyl)-2-(3-oxo-3,4-dihydro-2*H*-benzo[*b*][1,4]-thiazin-2-yl)-acetamido)-acetamide (**8dH**)

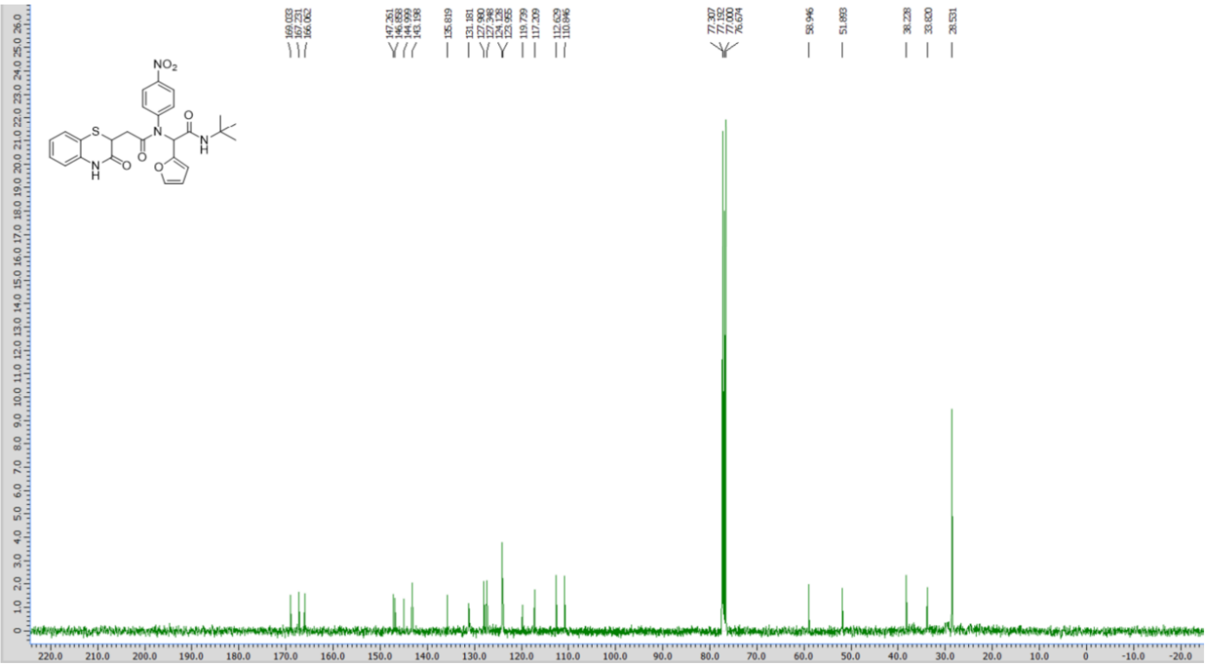

Figure S36: ^{13}C spectra of *N*-(*tert*-butyl)-2-(furan-2-yl)-2-(*N*-(4-nitrophenyl)-2-(3-oxo-3,4-dihydro-2*H*-benzo[*b*][1,4]-thiazin-2-yl)-acetamido)-acetamide (**8dH**)

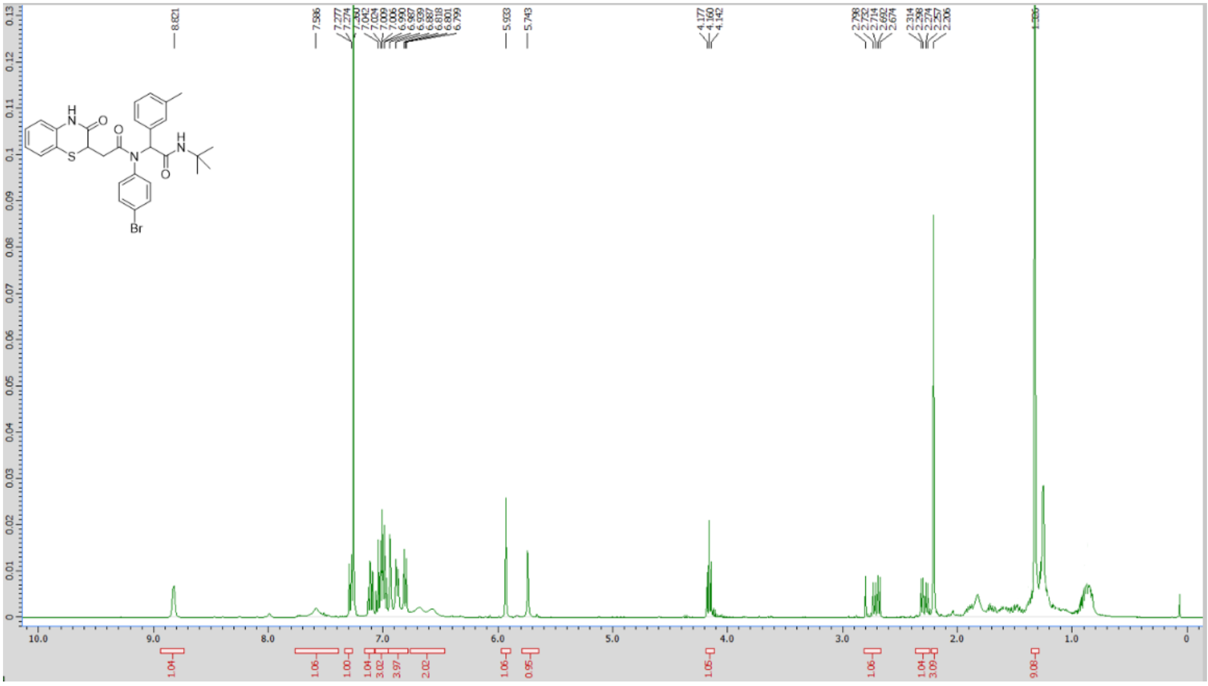

Figure S37: ^{1}H spectra of *N*-(4-bromophenyl)-*N*-(2-(*tert*-butylamino)-2-oxo-1-(m-tolyl)-ethyl)-2-(3-oxo-3,4-dihydro-2*H*-benzo[*b*][1,4]-thiazin-2-yl)-acetamide (**8eC**)

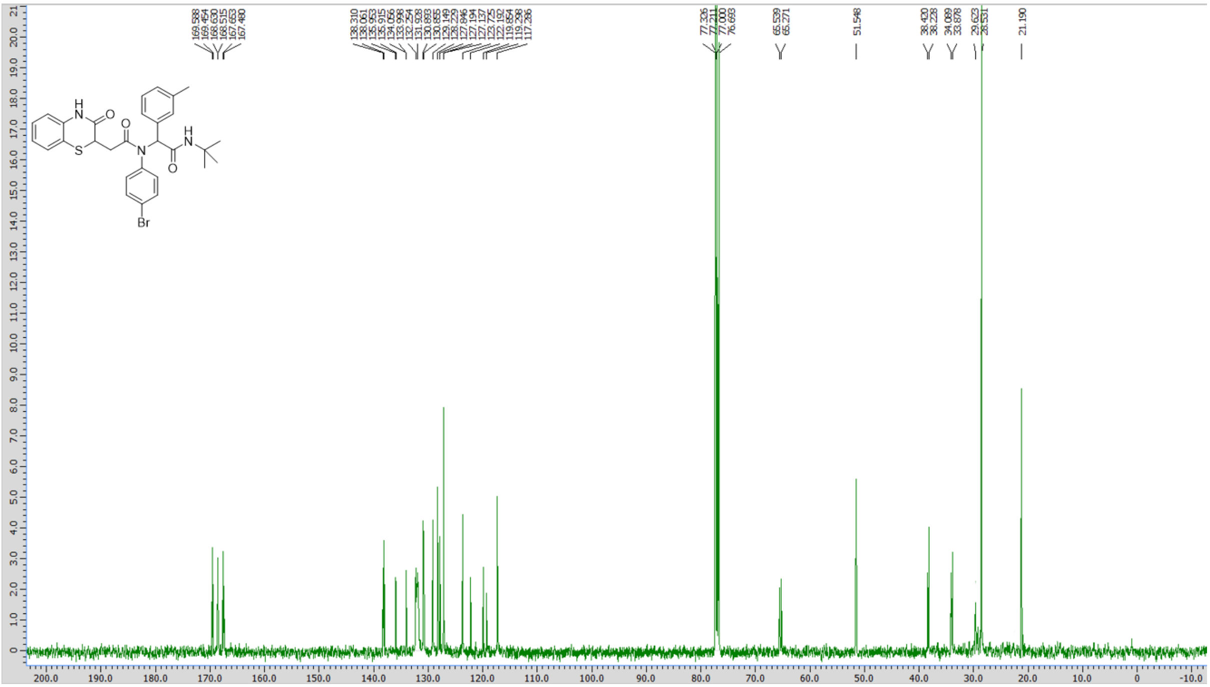

Figure S38: ^{13}C spectra of *N*-(4-bromophenyl)-*N*-(2-(*tert*-butylamino)-2-oxo-1-(m-tolyl)-ethyl)-2-(3-oxo-3,4-dihydro-2*H*-benzo[*b*][1,4]-thiazin-2-yl)-acetamide (**8eC**)

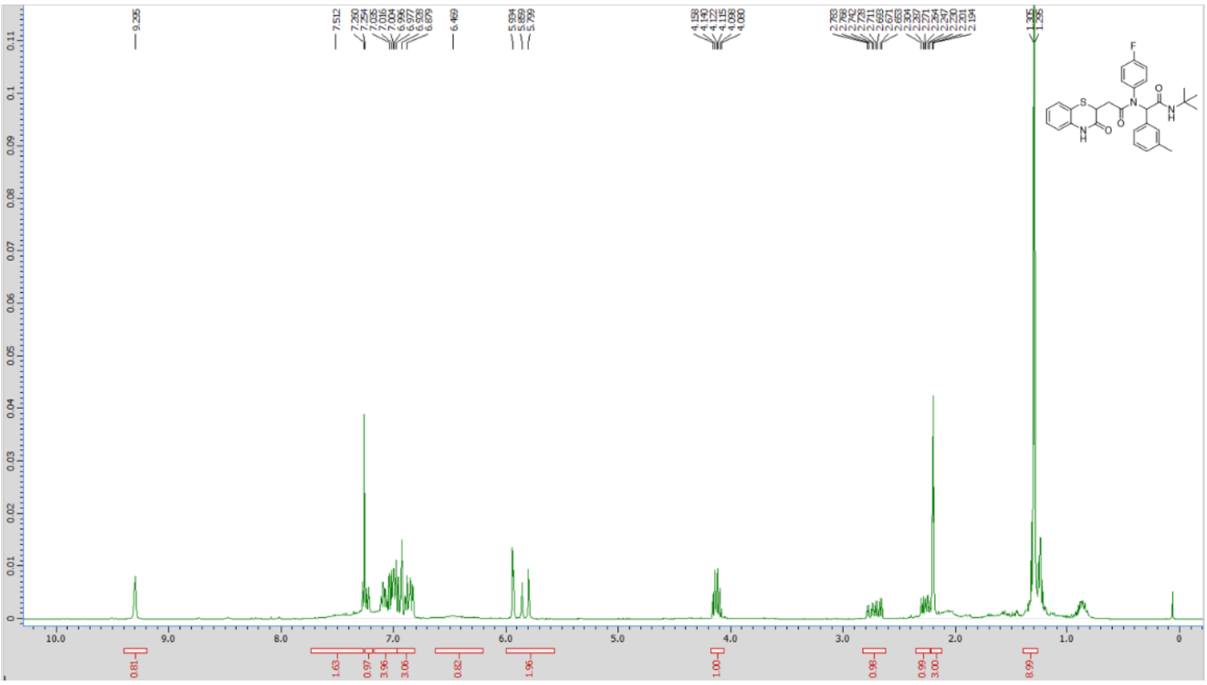

Figure S39: ^{1}H spectra of N-(tert-butyl)-2-(N-(4-fluorophenyl)-2-(3-oxo-3,4-dihydro-2H-benzo[b] [1,4]-thiazin-2-yl)-acetamido)-2-(m-tolyl)-acetamide (**8eD**)

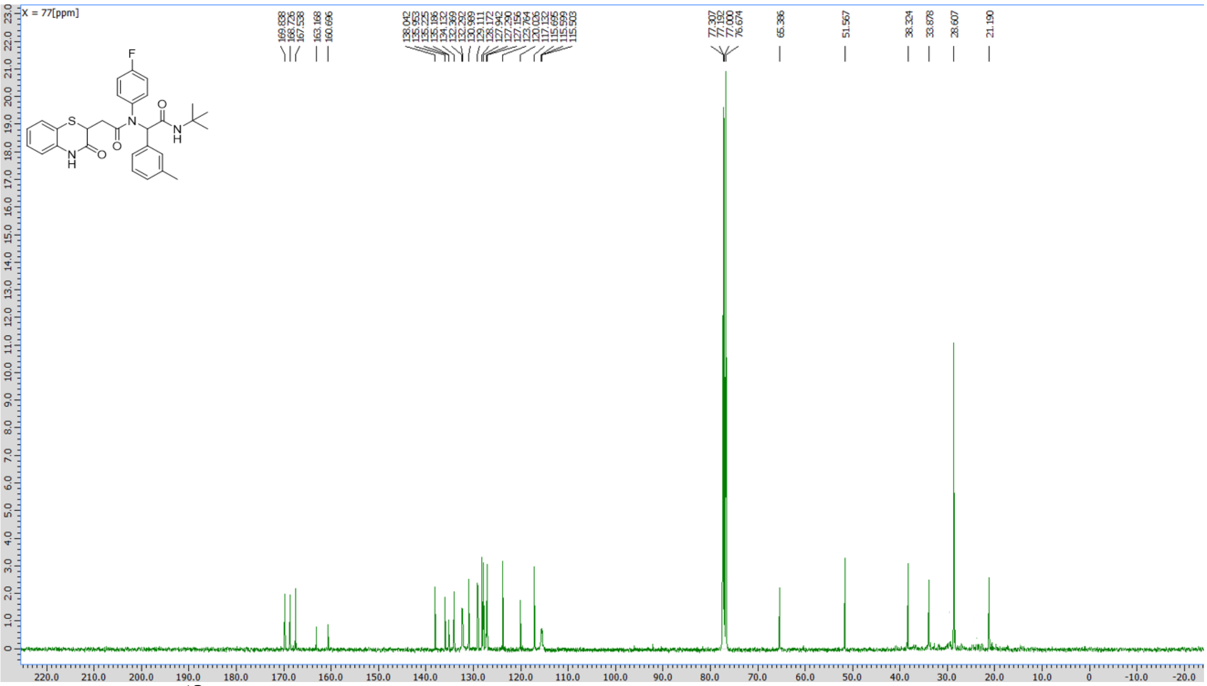

Figure S40: ^{13}C spectra of N-(tert-butyl)-2-(N-(4-fluorophenyl)-2-(3-oxo-3,4-dihydro-2H-benzo[b] [1,4]-thiazin-2-yl)-acetamido)-2-(m-tolyl)-acetamide (**8eD**)

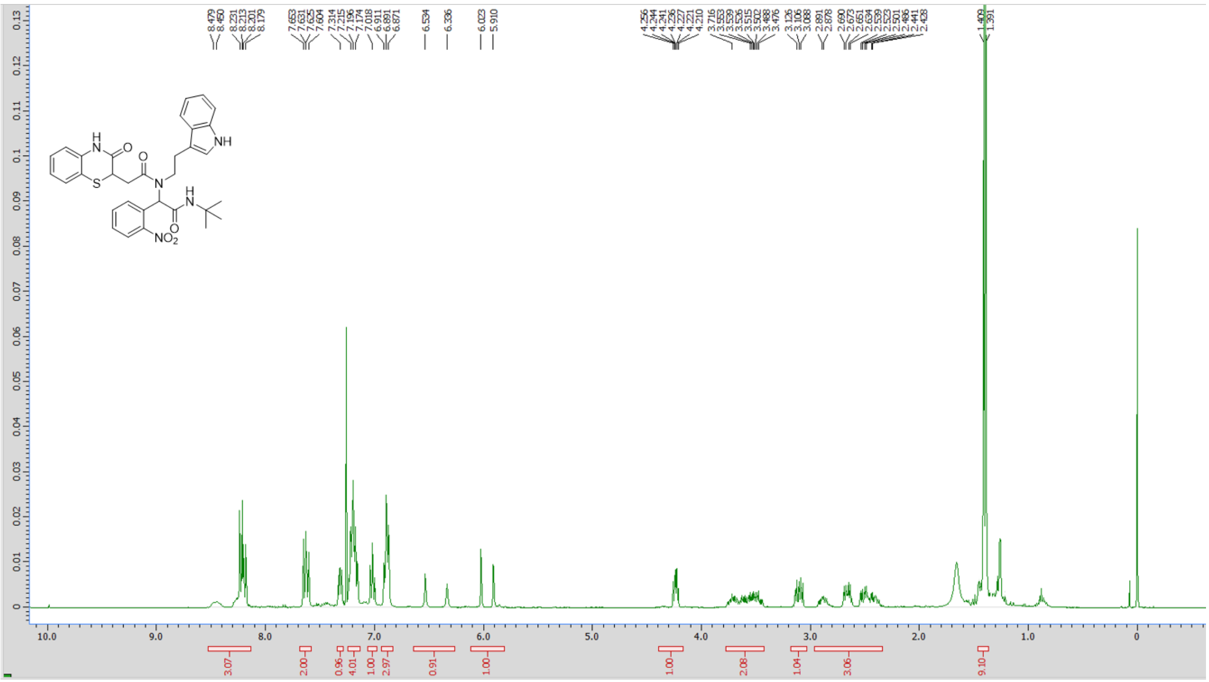

Figure S41: ^{1}H spectra of N-(2-(1H-indol-3-yl)-ethyl)-N-(2-(tert-butylamino)-1-(2-nitrophenyl)-2-oxoethyl)-2-(3-oxo-3,4-dihydro-2H-benzo[b][1,4-]-thiazin-2-yl)-acetamide (**8fE**)

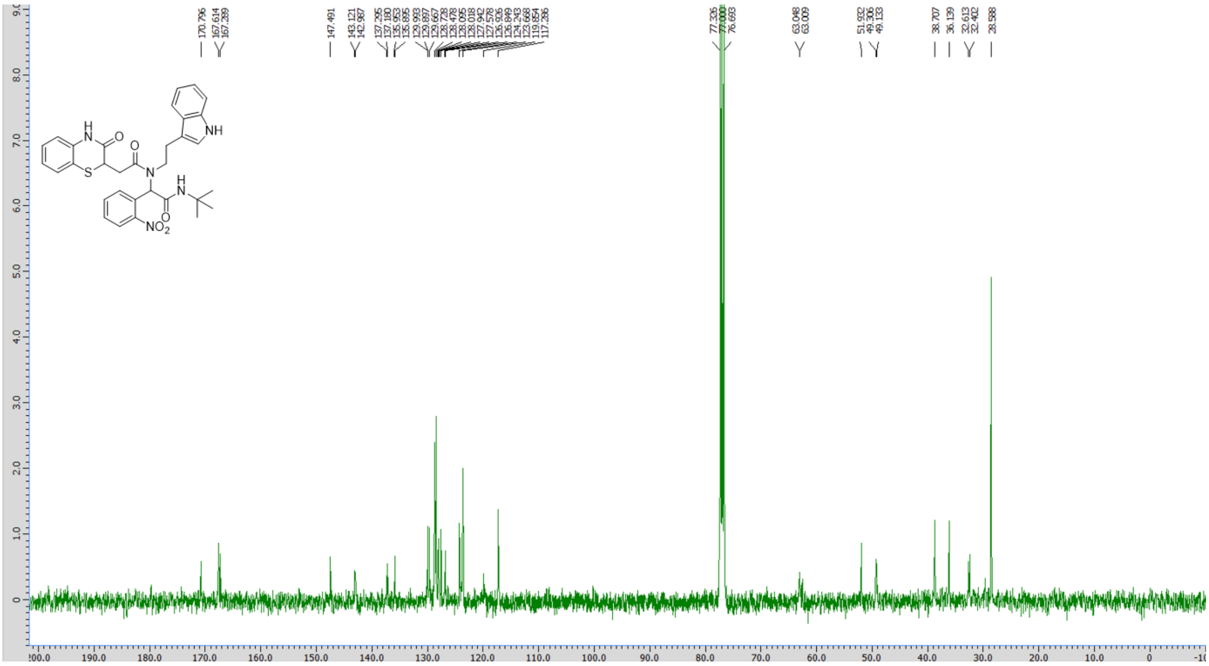

Figure S42: ^{13}C spectra of N-(2-(1H-indol-3-yl)-ethyl)-N-(2-(tert-butylamino)-1-(2-nitrophenyl)-2-oxoethyl)-2-(3-oxo-3,4-dihydro-2H-benzo[b][1,4-]-thiazin-2-yl)-acetamide (**8fE**)

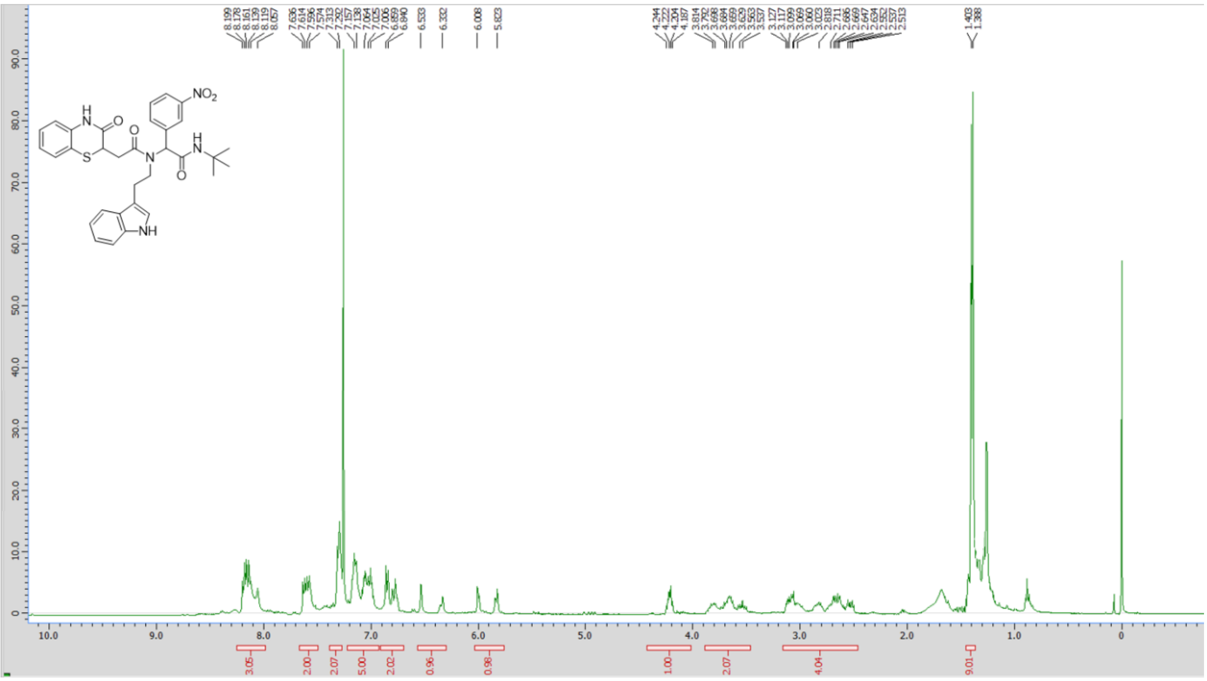

Figure S43: ^{1}H spectra of N-(2-(1H-indol-3-yl)-ethyl)-N-(2-(tert-butylamino)-1-(3-nitrophenyl)-2-oxoethyl)-2-(3-oxo-3,4-dihydro-2H-benzo[b][1,4]-thiazin-2-yl)-acetamide (**8gE**)

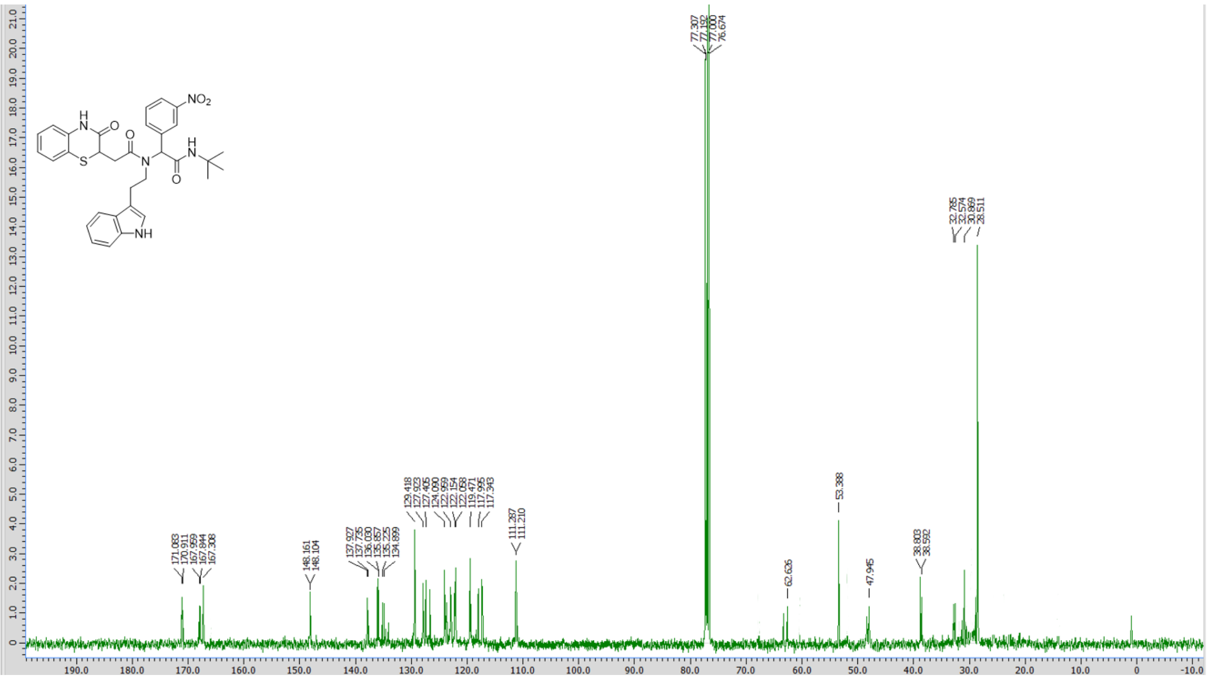

Figure S44: ^{13}C spectra of N-(2-(1H-indol-3-yl)-ethyl)-N-(2-(tert-butylamino)-1-(3-nitrophenyl)-2-oxoethyl)-2-(3-oxo-3,4-dihydro-2H-benzo[b][1,4]-thiazin-2-yl)-acetamide (**8gE**)

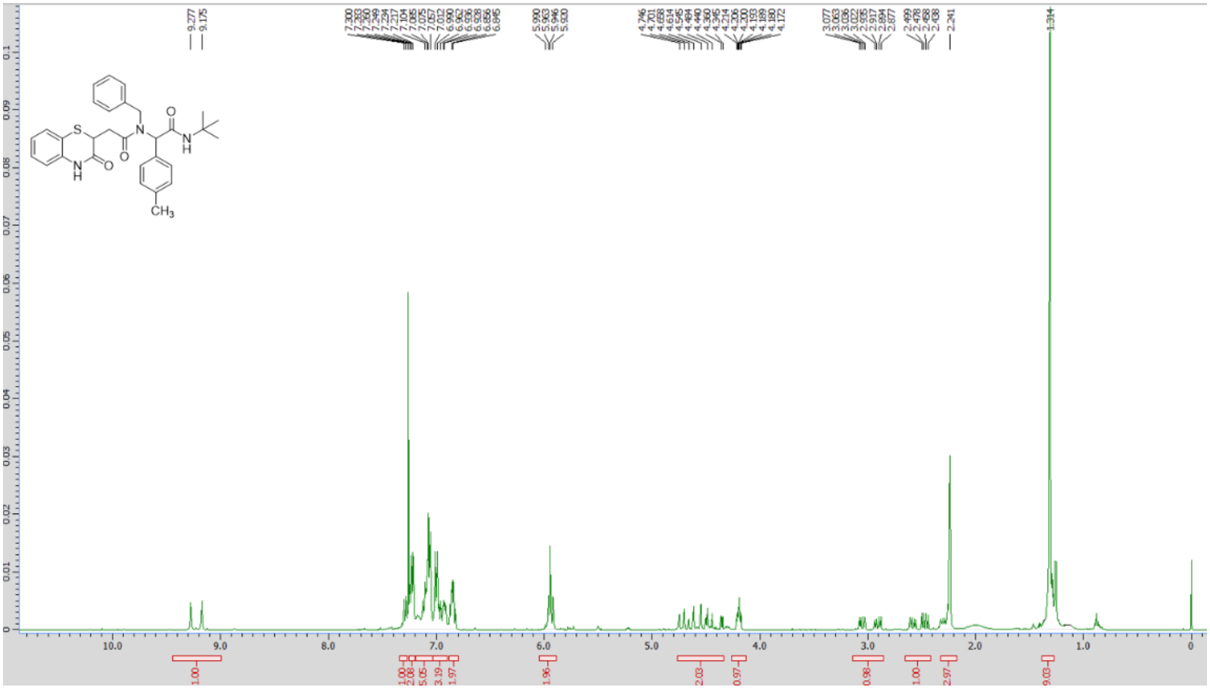

Figure S49: ^{1}H spectra of N-benzyl-N-(2-(tert-butylamino)-2-oxo-1-(p-tolyl)-ethyl)-2-(3-oxo-3,4-dihydro-2H-benzo[b][1,4]-thiazin-2-yl)-acetamide (**8hA**)

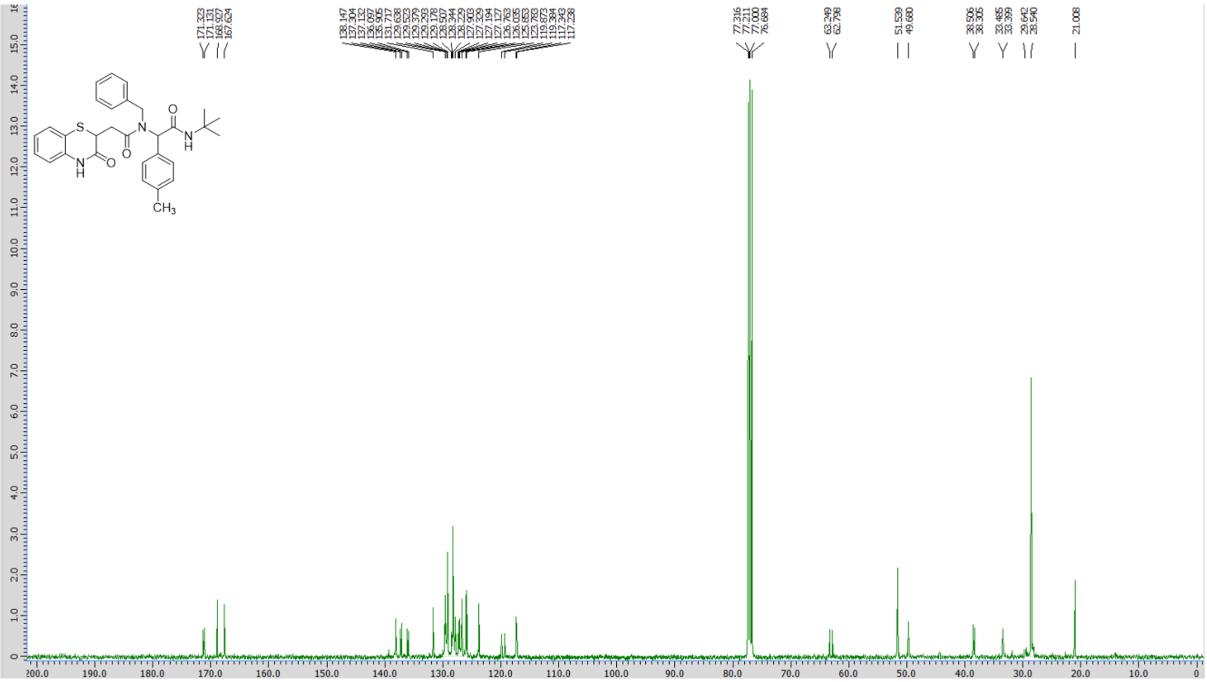

Figure S410: ^{13}C spectra of N-benzyl-N-(2-(tert-butylamino)-2-oxo-1-(p-tolyl)-ethyl)-2-(3-oxo-3,4-dihydro-2H-benzo[b][1,4]-thiazin-2-yl)-acetamide (**8hA**)

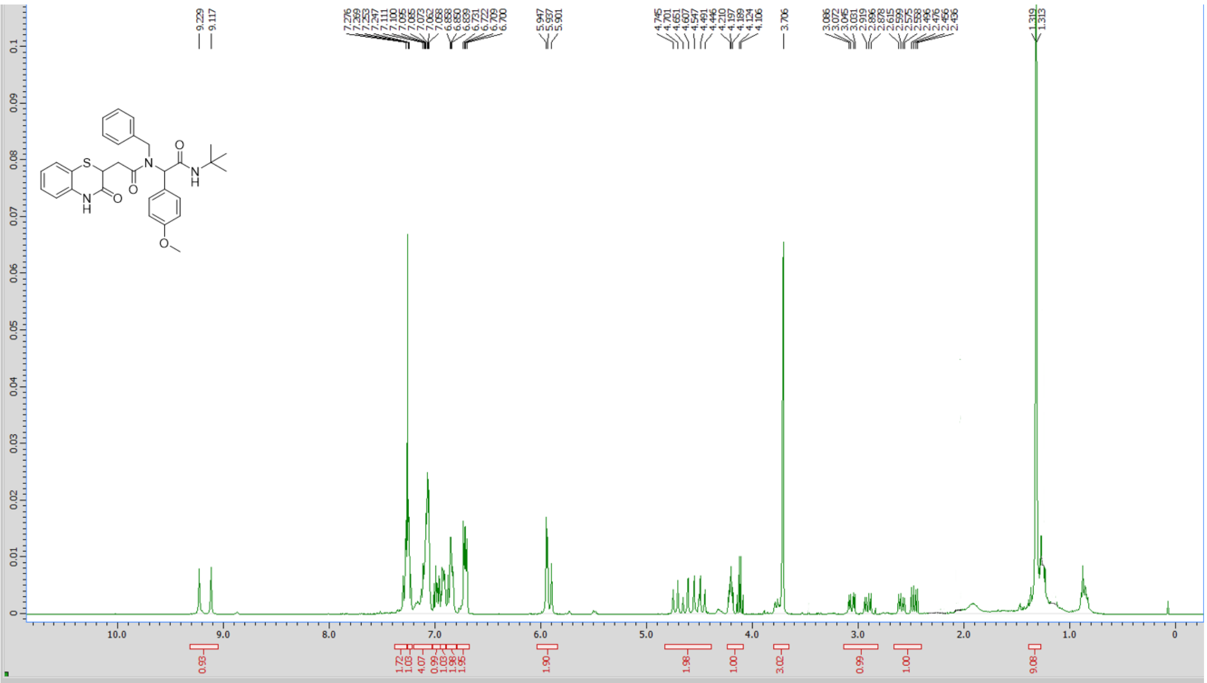

Figure S411: ^{1}H spectra of *N*-benzyl-*N*-(2-(*tert*-butylamino)-1-(4-methoxyphenyl)-2-oxoethyl)-2-(3-oxo-3,4-dihydro-2*H*-benzo[*b*][1,4]-thiazin-2-yl)-acetamide (**8iA**)

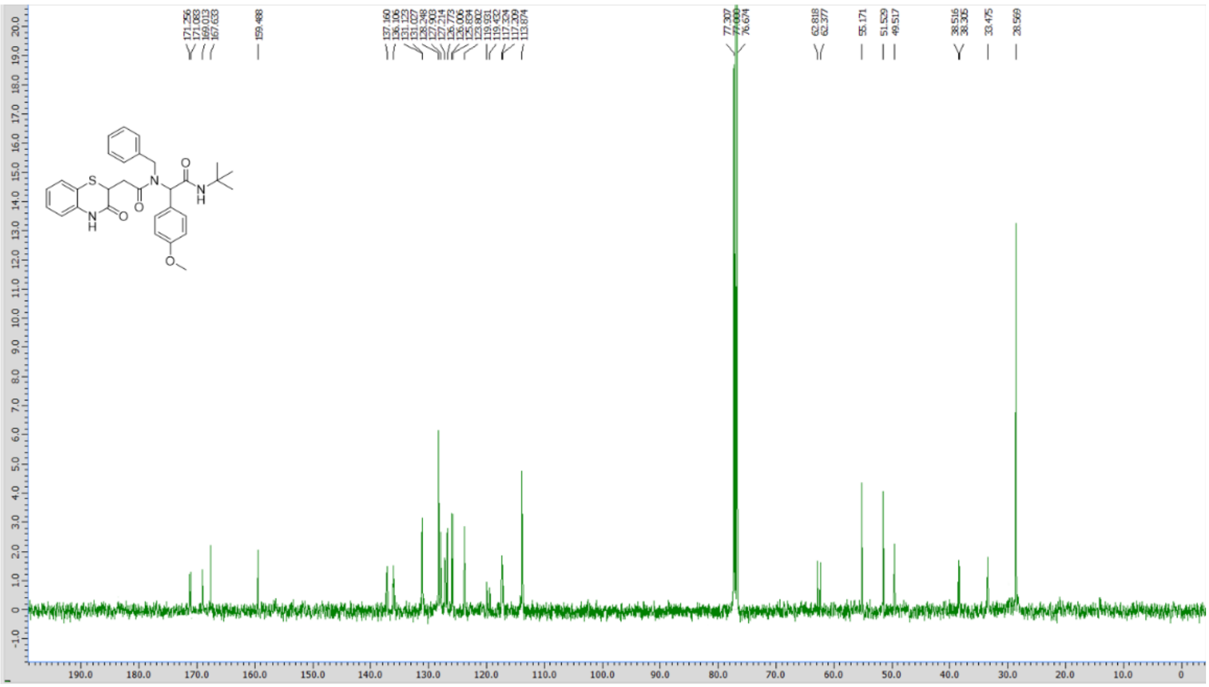

Figure S412: ^{13}C spectra of *N*-benzyl-*N*-(2-(*tert*-butylamino)-1-(4-methoxyphenyl)-2-oxoethyl)-2-(3-oxo-3,4-dihydro-2*H*-benzo[*b*][1,4]-thiazin-2-yl)-acetamide (**8iA**)

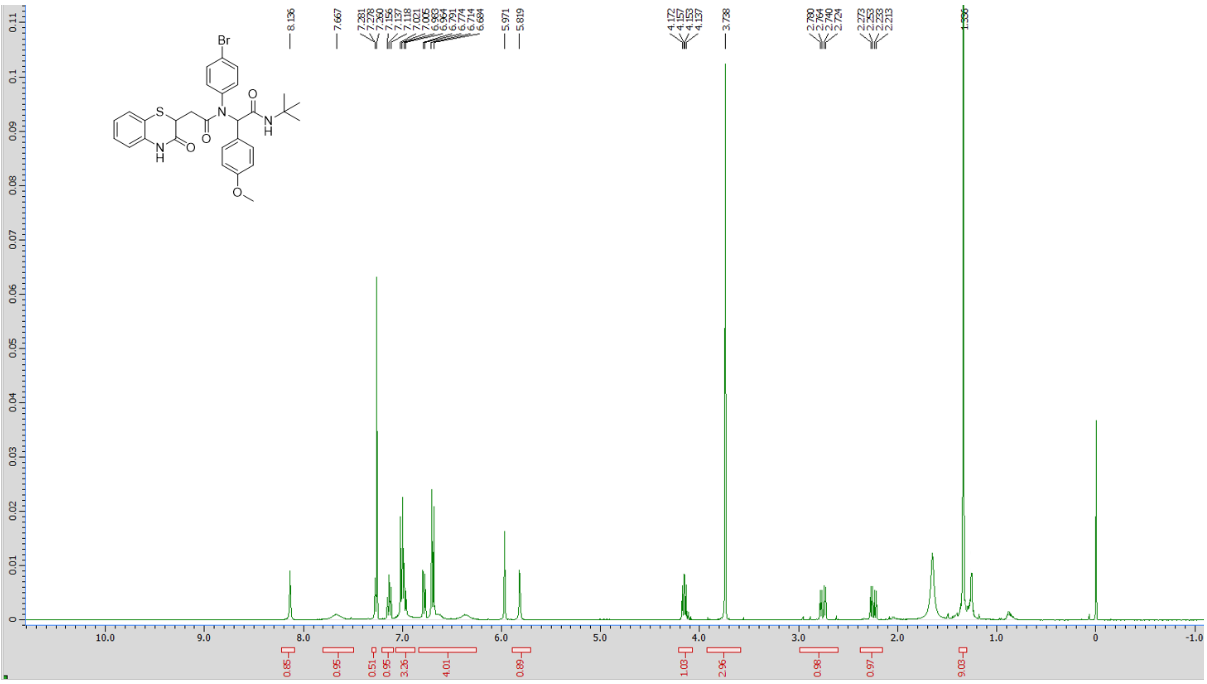

Figure S49: ^{1}H spectra of N-(4-bromophenyl)-N-(2-(*tert*-butylamino)-1-(4-methoxyphenyl)-2-oxoethyl)-2-(3-oxo-3,4-dihydro-2*H*-benzo[*b*][1,4]-thiazin-2-yl)-acetamide (**8iC**)

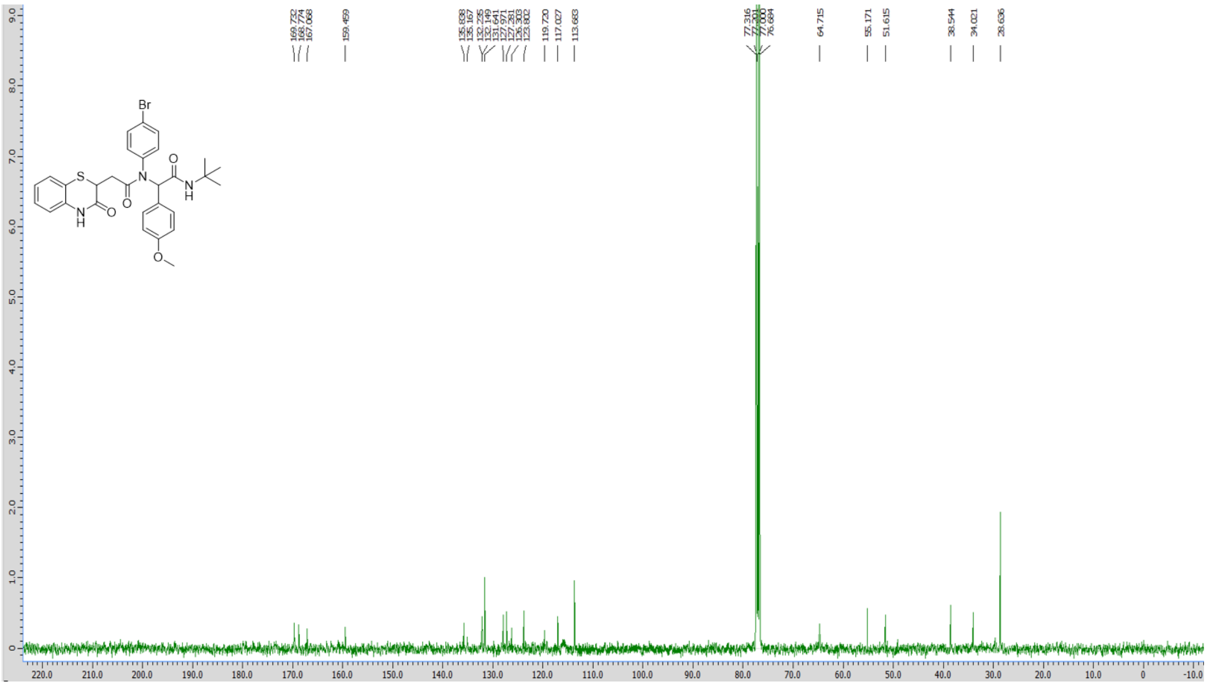

Figure S50: ^{13}C spectra of N-(4-bromophenyl)-N-(2-(*tert*-butylamino)-1-(4-methoxyphenyl)-2-oxoethyl)-2-(3-oxo-3,4-dihydro-2*H*-benzo[*b*][1,4]-thiazin-2-yl)-acetamide (**8iC**)

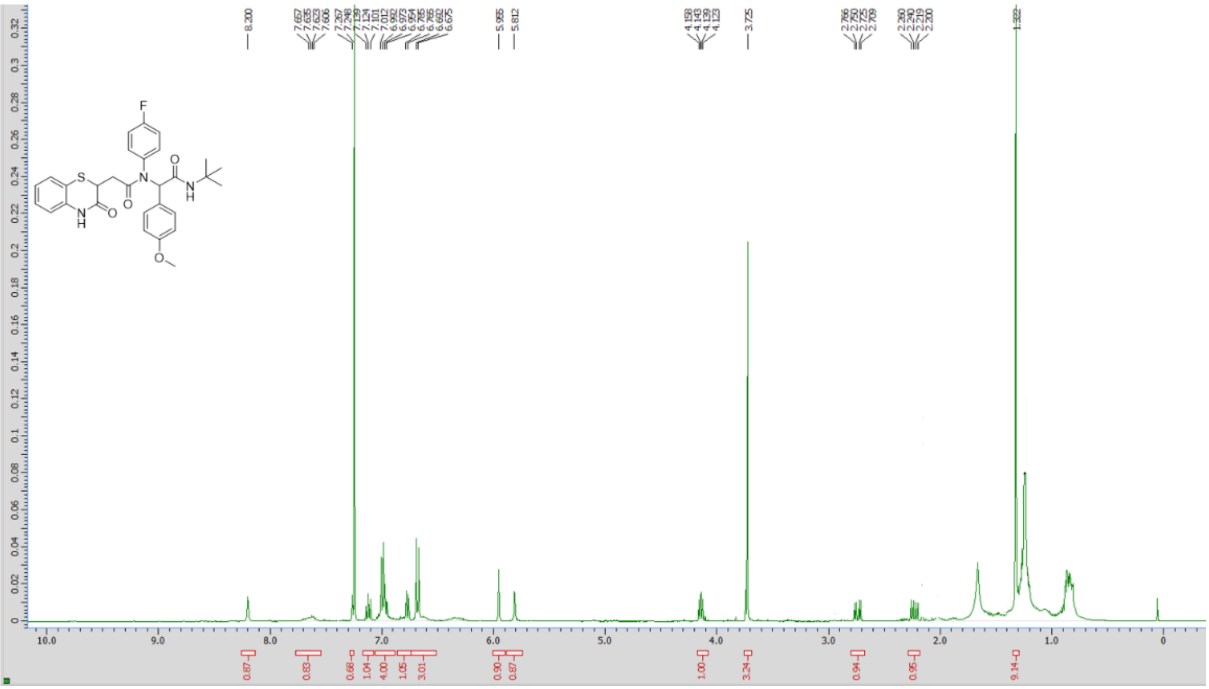

Figure S51: ^{1}H spectra of N-(*tert*-butyl)-2-(N-(4-fluorophenyl)-2-(3-oxo-3,4-dihydro-2*H*-benzo[*b*] [1,4]-thiazin-2-yl)-acetamido)-2-(4-methoxyphenyl)-acetamide (**8iD**)

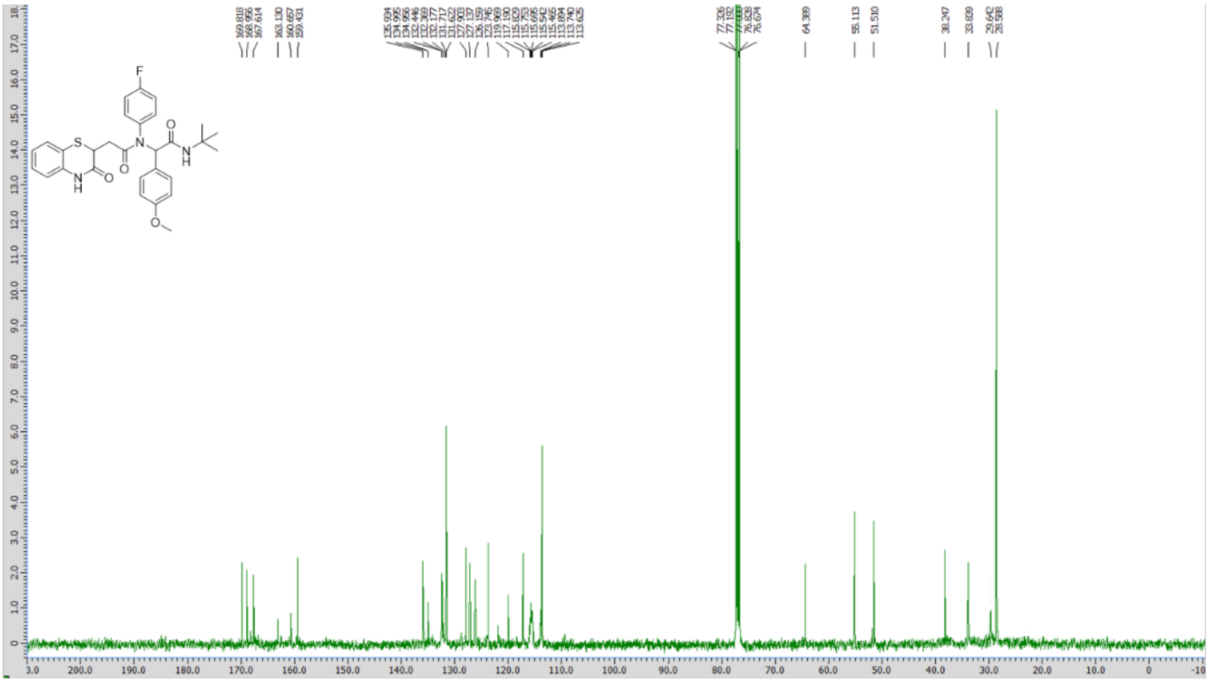

Figure 13: ^{13}C spectra of N-(*tert*-butyl)-2-(N-(4-fluorophenyl)-2-(3-oxo-3,4-dihydro-2*H*-benzo[*b*] [1,4]-thiazin-2-yl)-acetamido)-2-(4-methoxyphenyl)-acetamide (**8iD**)

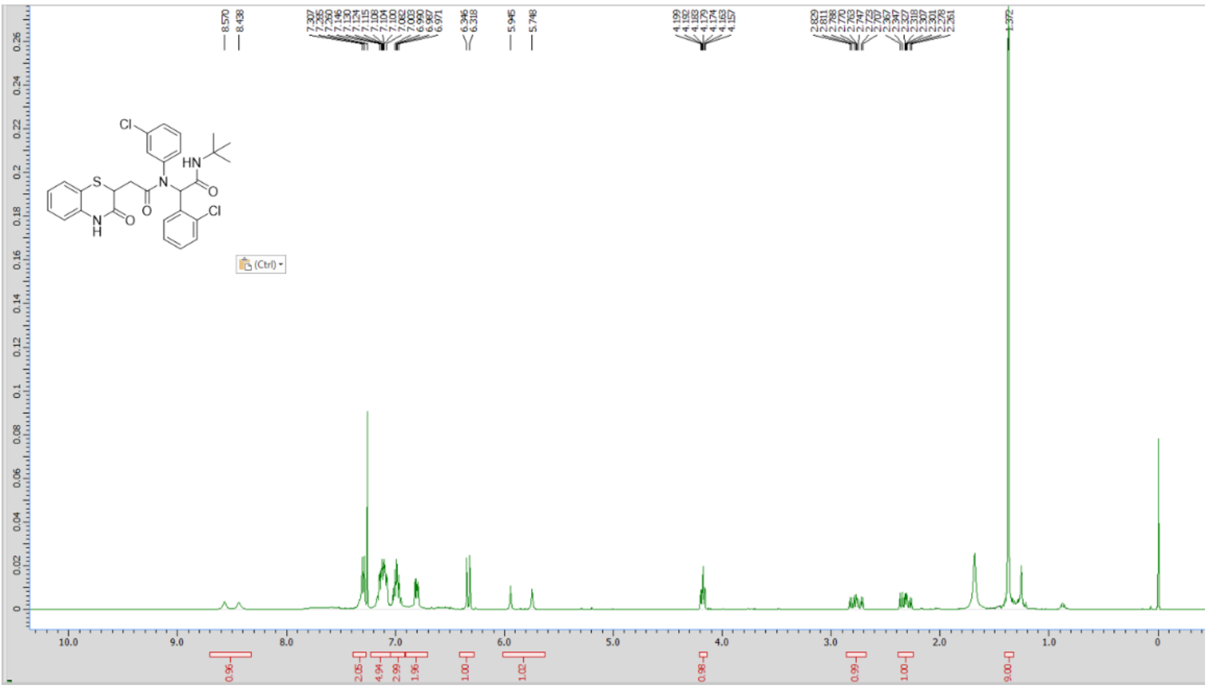

Figure 14: ^{1}H spectra of N-(*tert*-butyl)-2-(2-chlorophenyl)-2-(N-(3-chlorophenyl)-2-(3-oxo-3,4-dihydro-2*H*-benzo[*b*][1,4]-thiazin-2-yl)-acetamido)-acetamide (**8jB**)

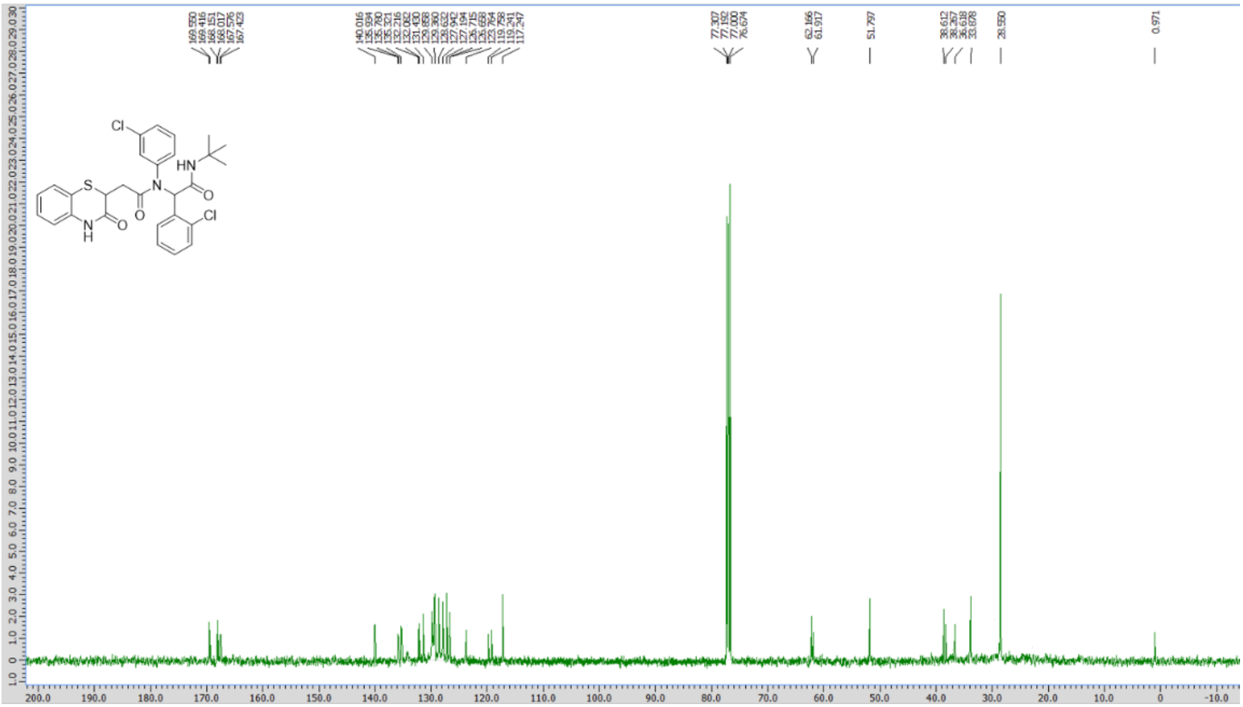

Figure 15: ^{13}C spectra of N-(*tert*-butyl)-2-(2-chlorophenyl)-2-(N-(3-chlorophenyl)-2-(3-oxo-3,4-dihydro-2*H*-benzo[*b*][1,4]-thiazin-2-yl)-acetamido)-acetamide (**8jB**)

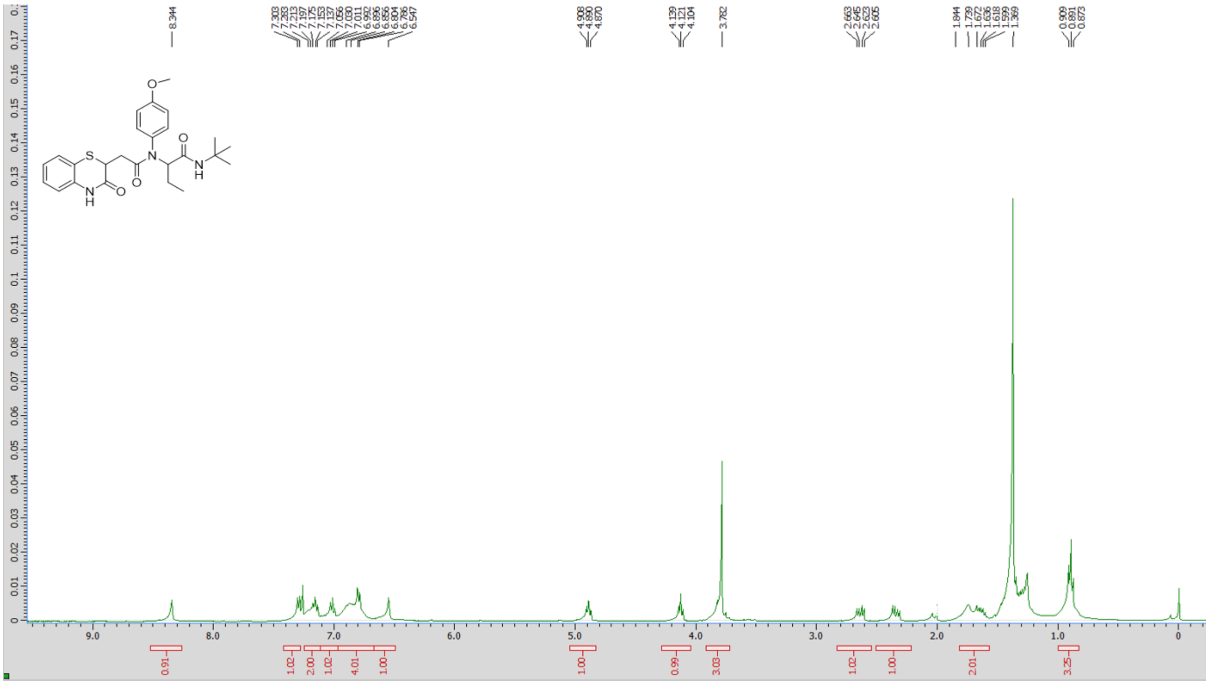

Figure S16: ^{1}H spectra of N-(*tert*-butyl)-2-(N-(4-methoxyphenyl)-2-(3-oxo-3,4-dihydro-2*H*-benzo[*b*] [1,4]-thiazin-2-yl)-acetamido)-butanamide (**8kG**)

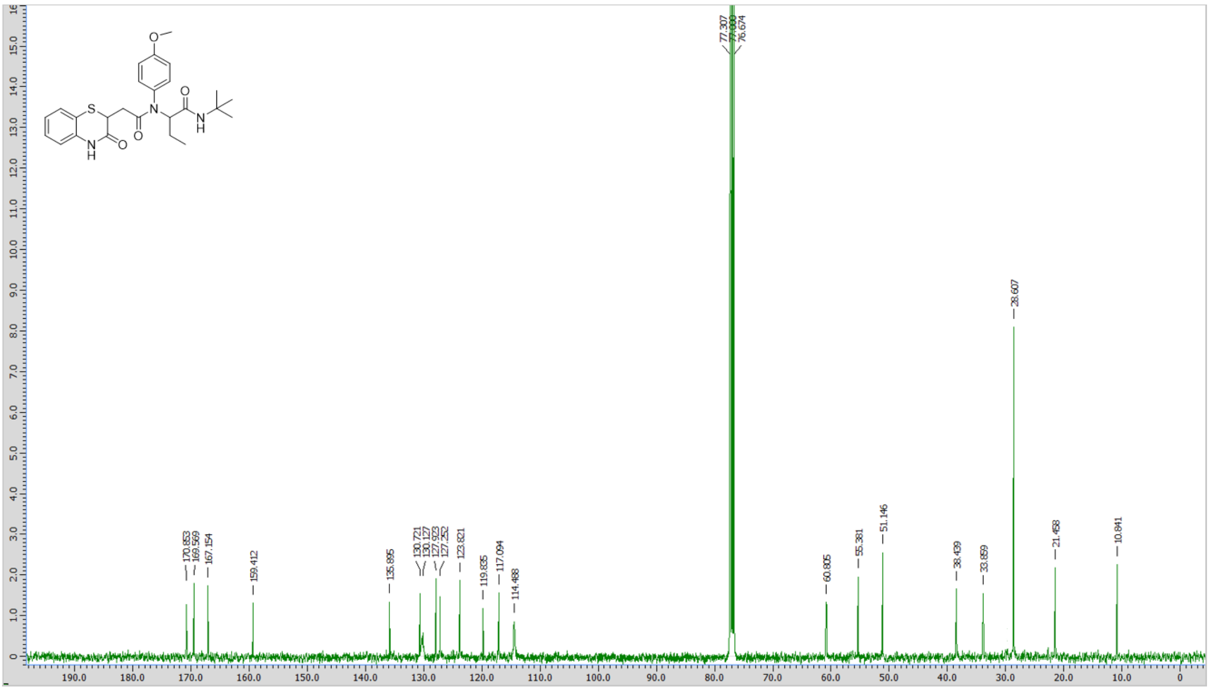

Figure S17: ^{13}C spectra of N-(*tert*-butyl)-2-(N-(4-methoxyphenyl)-2-(3-oxo-3,4-dihydro-2*H*-benzo[*b*] [1,4]-thiazin-2-yl)-acetamido)-butanamide (**8kG**)

Table S1: Docking scores of synthetic compounds

Compound	Structure	Docking Score (Kcal/mol)	Total H Bonds	H bonded Amino acids	H-bond Distance (Å)	Other Interacting amino acids (Except Van der Waals)
**8aA**	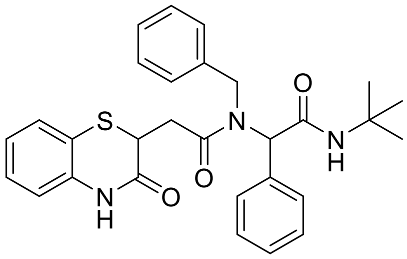	**-7.8**	**1**	**Asn:117**	**2.05**	**(8 interactions) Arg:56, Val:59, Leu:112, Glu:185, Val:151, His:154, Gly:110**
**8aC**	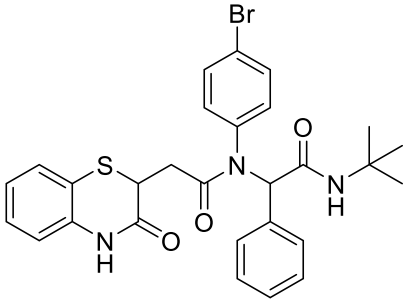	**-8.0**	**2**	**Gly:110**	**2.11<br>1.90**	**(7 interactions) Leu:105, Val:151, His:154, Leu:112, Val:59, Arg:56**
**8aD**	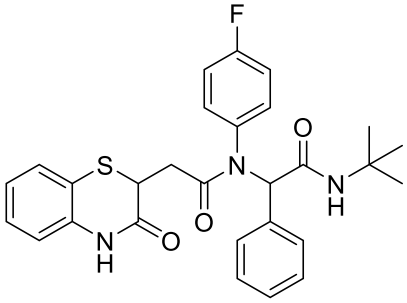	**-8.2**	**2**	**Gly:110**	**2.11<br>1.94**	**(6 interactions) Leu:105, Val:151, His:154, Leu:112, Val:59, Arg:56**
**8aJ**	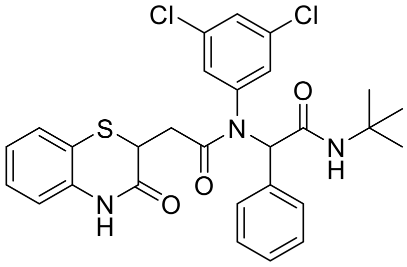	**-8.6**	**1**	**Gly:110**	**1.97**	**(7 interactions) Val:151, Leu:105, His:154, Leu:112, Gly:60**
**8bA**	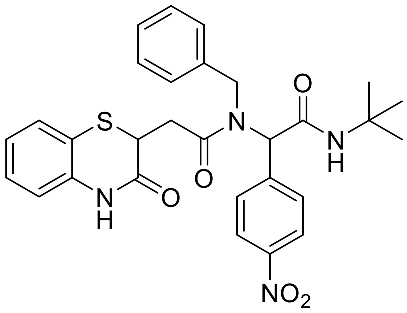	**-8.7**	**6**	**Gly:110<br>Arg:56<br>Val:59<br>Asn:117<br>Leu:112<br>Gln:65**	**2.72<br>2.89<br>2.70<br>1.96<br>2.01<br>2.87**	**(4 interactions) Val:151, His:154**

**8bB**	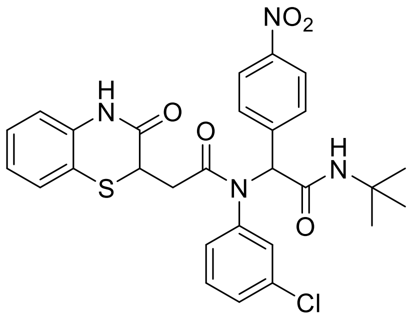	**-9.1**	**3**	**Gly:110 Tyr:147 His:154**	**2.39 2.46 3.00**	**(7 interactions) Glu:185, Gly:110, His:154, Leu:105, Val:59, Leu:112**
**8bC**	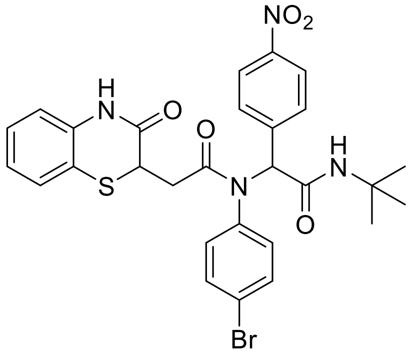	**-8.5**	**6**	**Val:59 Asn:117 Leu:112 Gln:65 Glu:155 Csd:111 (Cys Modifie d)**	**2.48 2.29 1.89 2.16 2.78 3.35**	**(8 interactions) Arg:56, Glu:185, His:154, Val:151, Ile:150, Leu:105**
**8bD**	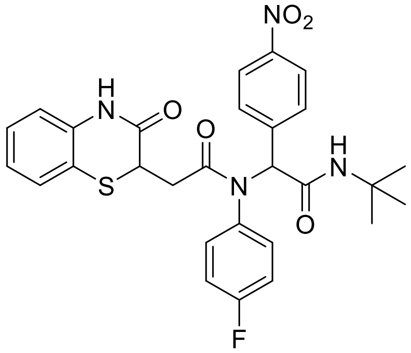	**-8.6**	**6**	**Val:59 Asn:117 Leu:112 Gln:65 Glu:155 Csd:111**	**2.59 2.23 1.90 2.24 2.77 3.38**	**(4 interactions) Arg:56, His:154**
**8bE**	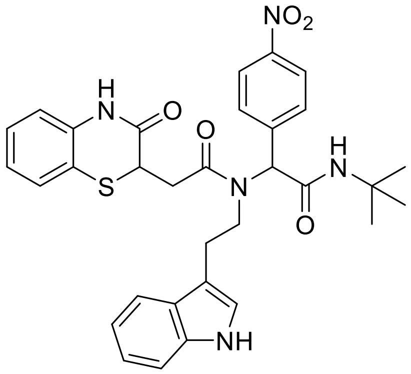	**-8.3**	**4**	**Val:59 Asn:117 Gln:65 Tyr:147**	**2.93 2.27 2.37 1.87**	**(6 interactions) Arg:56, Val:151, Leu:105, His:154, Leu:112**

**8bF**	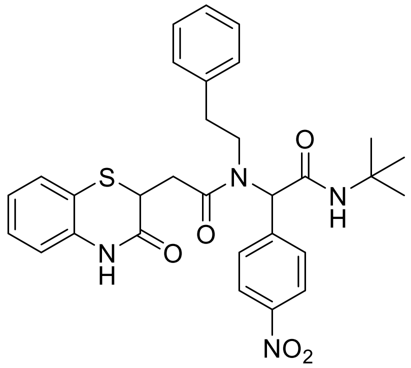	**-7.0**	**3**	**Asn:117<br>Gly:110**	**2.38<br>2.31<br>1.85**	**(6 interactions)<br>Val:151, His:154, Tyr:147, Val:59, Ser:57**
**8bI**	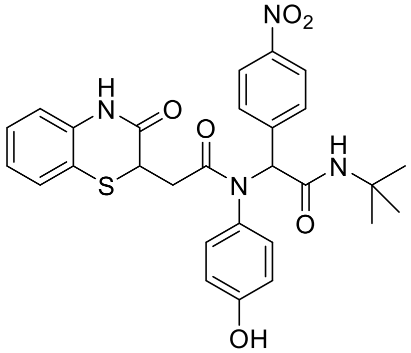	**-8.4**	**2**	**Gly:110<br>Gly:60**	**2.54<br>2.68**	**(6 interactions)<br>Pro:78, Leu:112, His:154, Val:151**
**8cC**	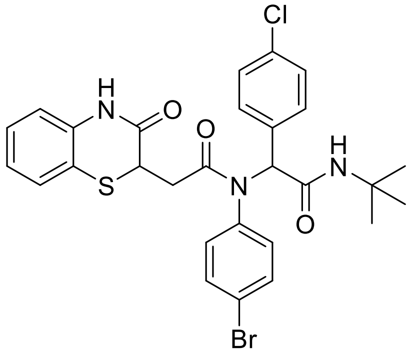	**-7.9**	**2**	**Gly:110<br>Val:59**	**2.23<br>2.79**	**(11 interactions)<br>Leu:105, Tyr:147, Ile:150, Val:151, His:154, Leu:112, Gly:58, Arg:56, Ser:57**
**8cD**	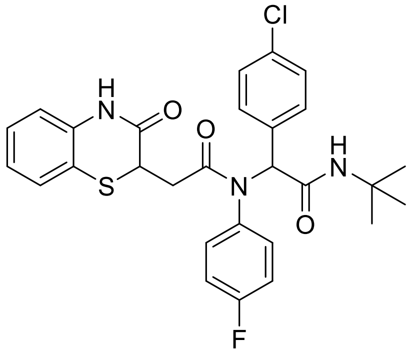	**-8.4**	**2**	**Ser:57<br>Tyr:147**	**2.18<br>2.29**	**(4 interactions)<br>Glu:109, His:154, Val:151, Leu:112**

**8cE**	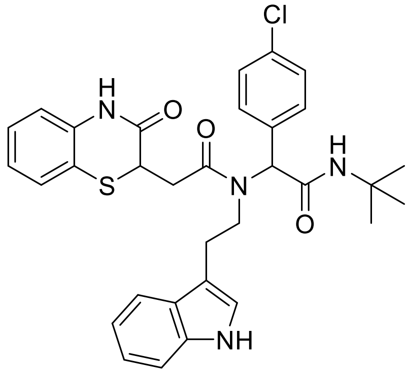	**-9.0**	**2**	**Gly:110**<br>**Ser:57**	**2.46**<br>**2.40**	**(9 interactions)**<br>**Leu:105, Tyr:147, Ile:150, Val:151, His:154, Leu:112, Gly:58, Pro:78**
**8cF**	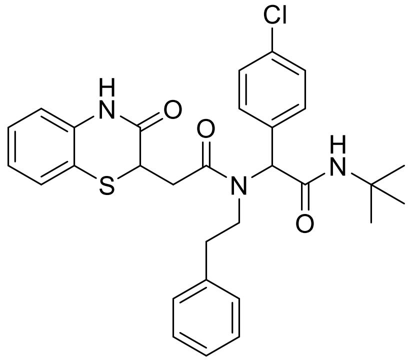	**-8.1**	**2**	**Ser:57**<br>**Gly:110**	**2.47**<br>**2.49**	**(8 interactions)**<br>**Val:151, Leu:105, Glu:185, Pro:78, Ile:150, Leu:112, His:154**
**8cI**	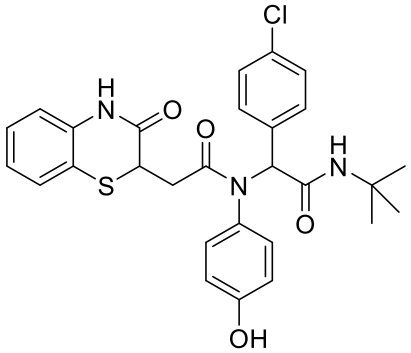	**-7.8**	**2**	**Leu:112**<br>**Gly:110**	**2.47**<br>**2.22**	**(6 interactions)**<br>**Val:59, His:154, Leu:105, Ile:50, Val:151**
**8dG**	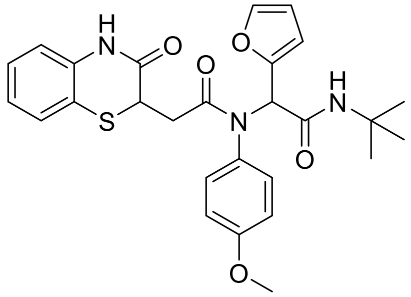	**-7.7**	**2**	**Gly:110**	**2.16**<br>**2.31**	**(6 interactions)**<br>**Val:151, Leu:112, Leu:105, Val:59, Arg:56**
**8dH**	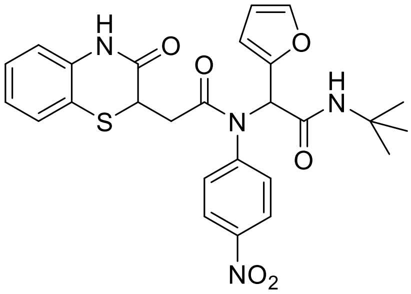	**-8.0**	**3**	**Asn:117**<br>**Arg:56**	**2.33**<br>**2.16**<br>**2.29**	**(6 interactions)**<br>**Pro:78, Leu:112, Val:151, Gly:110, His:154**

**8eC**	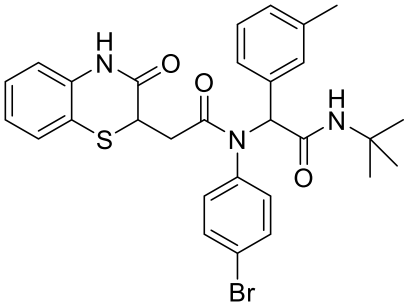	**-8.3**	**3**	**Gly:110**<br>**Val:59,**<br>**Tyr:147**	**2.39**<br>**2.10**<br>**2.37**	**(4 interactions)**<br>**His:154, Val:151,**<br>**Leu:112**
**8eD**	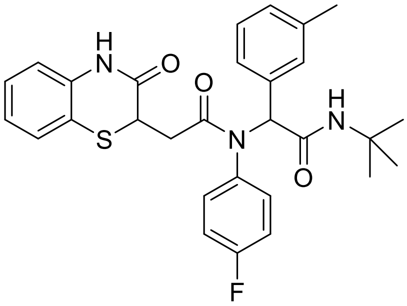	**-7.5**	**3**	**Val:59**<br>**Gly:60**	**2.22**<br>**2.87**<br>**2.84**	**(2 interactions)**<br>**Pro:78, Val:151**
**8fE**	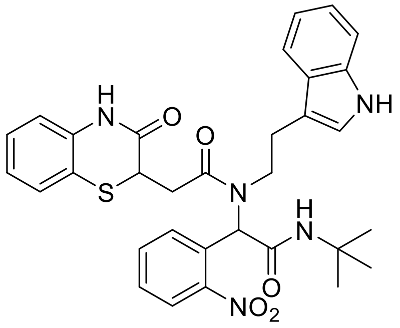	**-7.5**	**3**	**Arg:56**<br>**Gly:110**	**2.23**<br>**2.90**<br>**2.07**	**(7 interactions)**<br>**Leu:112, Glu:155,**<br>**Csd:111, Pro:78,**<br>**Val:59**
**8gE**	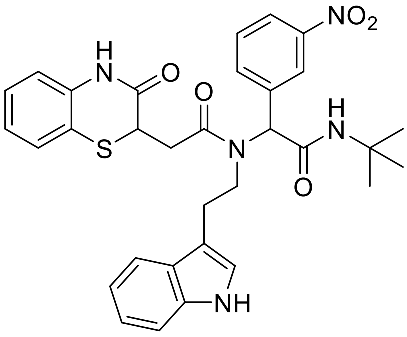	**-8.4**	**5**	**Arg:56**<br>**Val:59**<br>**Gly:60**<br>**Tyr:147**<br>**Asn:117**	**2.01**<br>**2.29**<br>**2.32**<br>**1.77**<br>**2.91**	**(7 interactions)**<br>**Leu:112, Gly:110,**<br>**Glu:185, Val:151,**<br>**His:154**
**8hA**	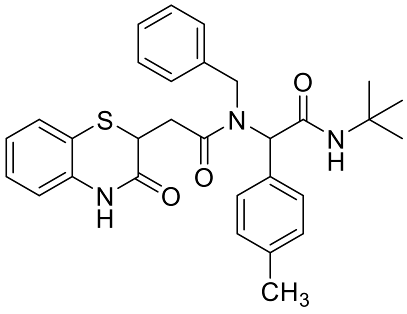	**-8.1**	**2**	**Asn:117**<br>**Val:59**	**2.09**<br>**2.41**	**(7 interactions)**<br>**Arg:56, Leu:112,**<br>**His:154, Val:151**

**8iA**	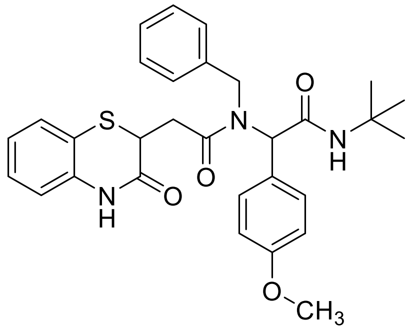	**-7.7**	**-**	**-**	**-**	**(8 interactions) Pro:78, Leu:105, Val:151, Glu:185, His:154, Leu:112, Gly:110**
**8iC**	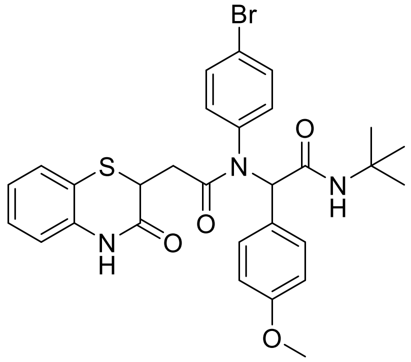	**-7.3**	**3**	**Gly:60<br>Val:59**	**2.60<br>2.05<br>2.92**	**(3 interactions) His:186, Pro:78, Val:151**
**8iD**	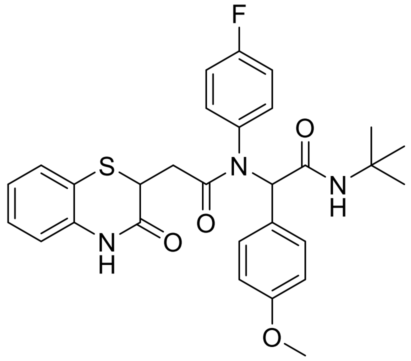	**-7.4**	**5**	**Val:59<br>Gly:60<br>Asn:117<br>Ser:57**	**2.93<br>2.10<br>2.53<br>2.65<br>2.97**	**(3 interactions) His:186, Pro:78, Val:151**
**8jB**	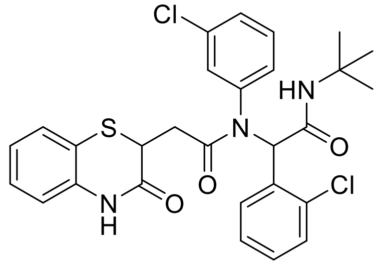	**-7.4**	**2**	**Gly:110<br>Val:59**	**2.62<br>2.38**	**(4 interactions) Leu:112, Leu:105, Val:151**
**8kG**	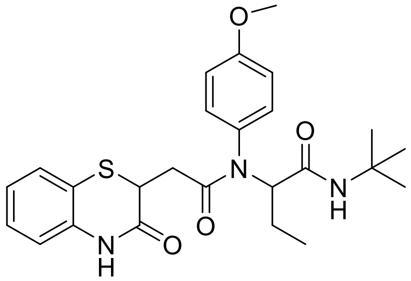	**-7.3**	**3**	**Arg:56<br>Gly:110<br>Tyr:147**	**2.36<br>2.27<br>1.88**	**(6 interactions) Leu:105, Val:151, Leu:112, Val:59**

**Standard (Actinonin)**	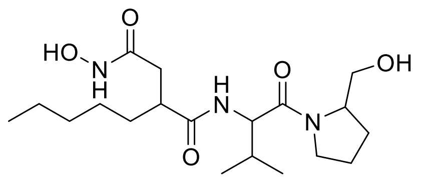	**-7.1**	**5**	**Gln:65**<br>**Gly:60**<br>**Leu:112**<br>**Glu:155**<br>**Val:59**	**2.82**<br>**2.41**<br>**2.47**<br>**2.70**<br>**1.76**	**(2 interactions)**<br><br>**Val:151**<br>**His:154**

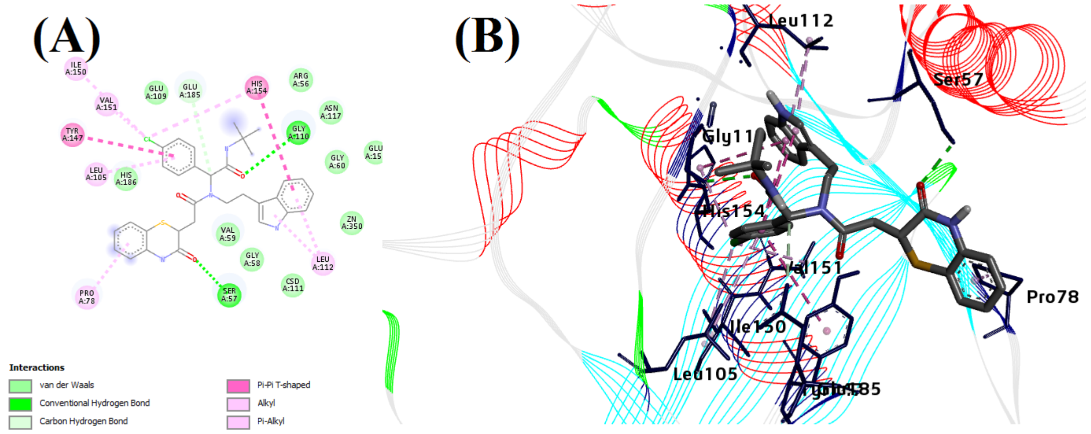

Figure S57. Molecular docking of the compound **8cE** against peptide deformylase. (A) 2D interaction image of compound **8cE** within the PDF binding pocket. The different interactions were highlighted in the figure; (B) 3D interaction image of compound **8cE** within the PDF binding pocket. Protein reported in line-ribbon. The blue color stick model indicates the interacting amino acid residues. The elemental color stick model indicates the ligand (grey color indicates hydrocarbons; blue indicates N; red indicating O, white indicates polar H, yellow indicates S).

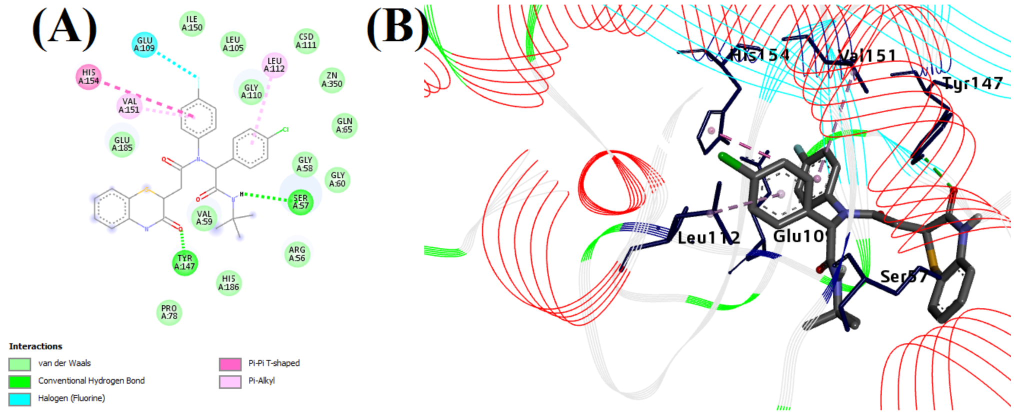

Figure S58. Molecular docking of the compound **8cD** against peptide deformylase. (A) 2D interaction image of compound **8cD** within the PDF binding pocket. The different interactions were highlighted in the figure; (B) 3D interaction image of compound **8cD** within the PDF binding pocket. Protein reported in line-ribbon. The blue color stick model indicates the interacting amino acid residues. The elemental color stick model indicates the ligand (grey color indicates hydrocarbons; blue indicates N; red indicating O, white indicates polar H, yellow indicates S).

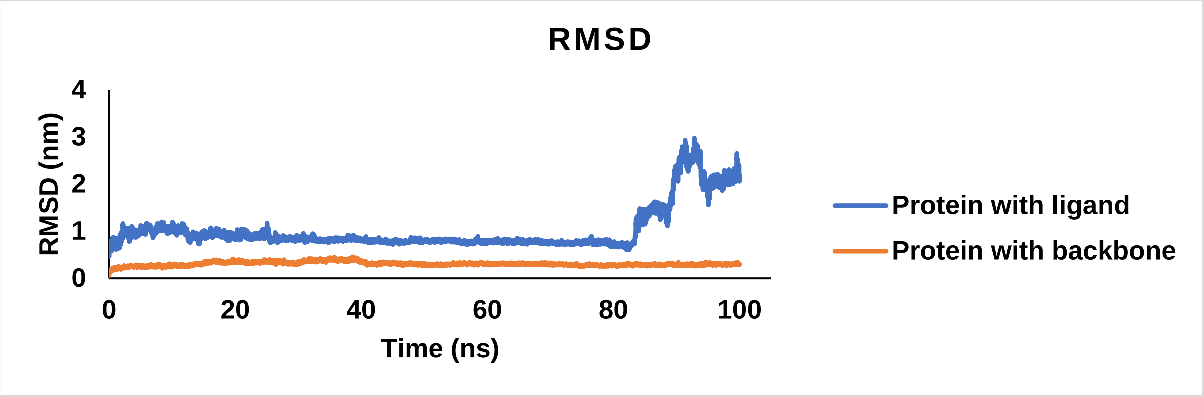

Figure S59. RMSD curve of 8bE-complex and protein

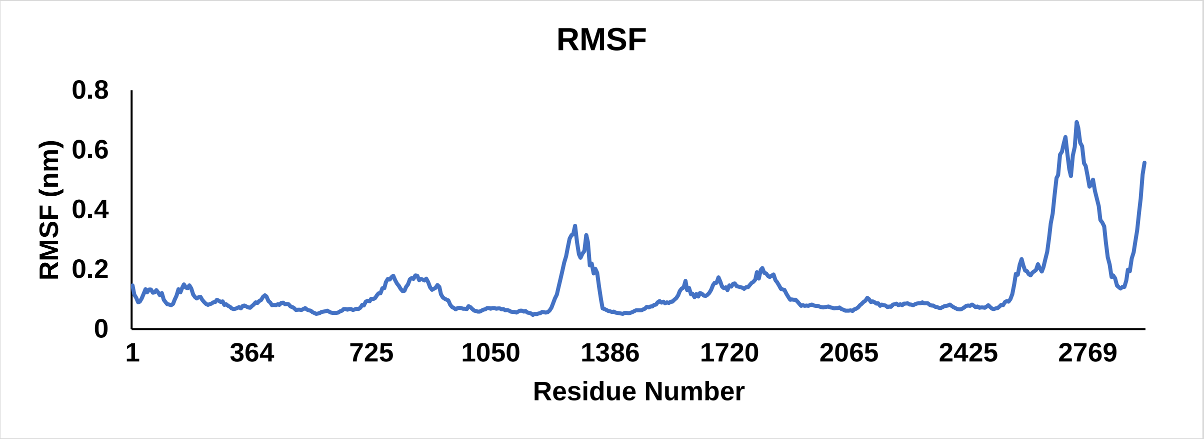

Figure S60. RMSF curve of complex

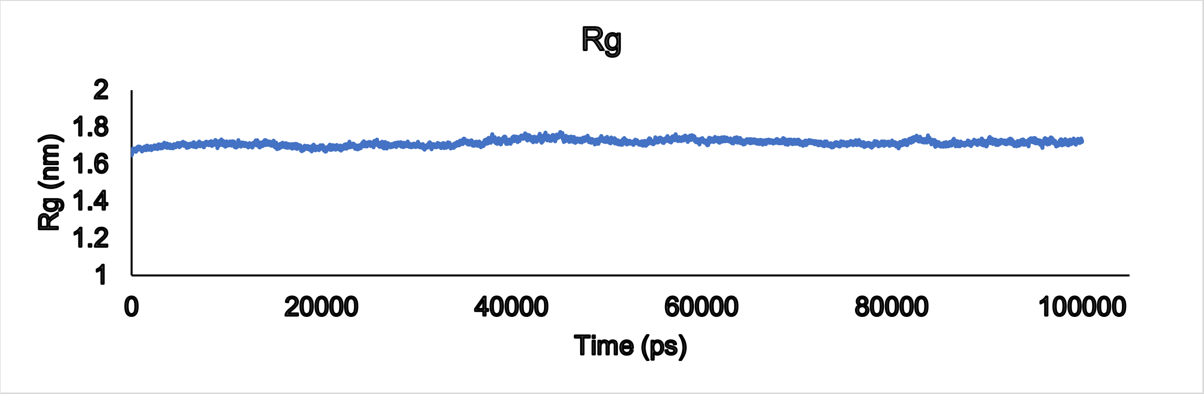

Figure S61. Rg curve of complex

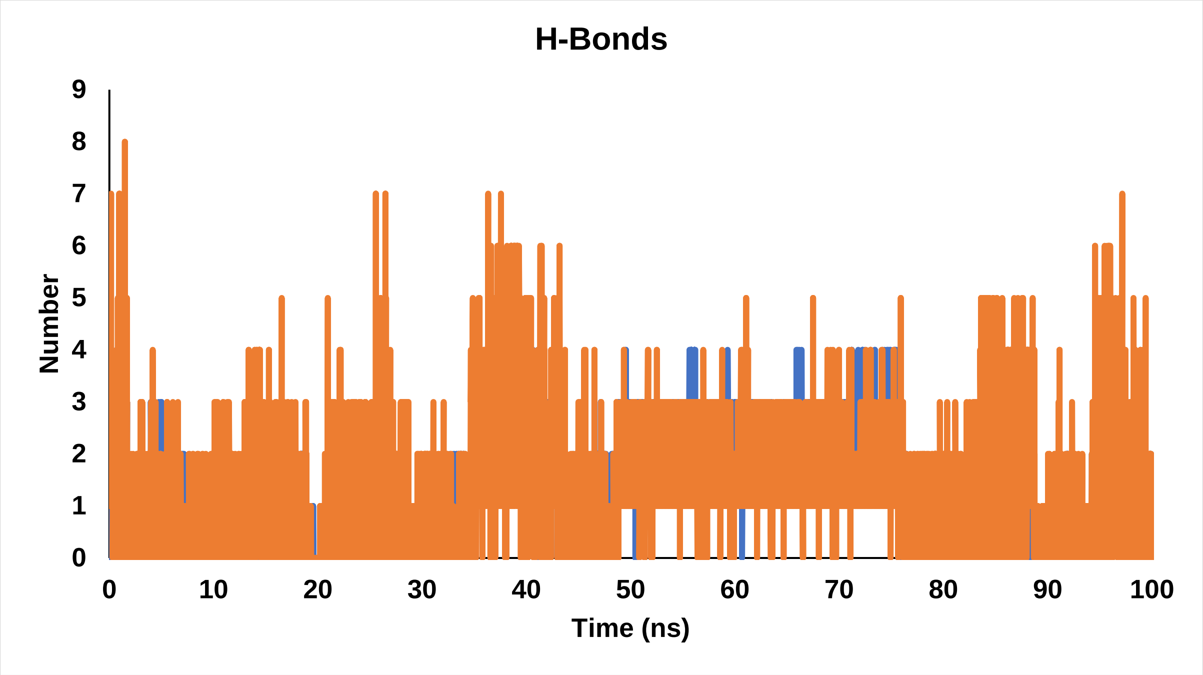

Figure S62. H-bonds of complex

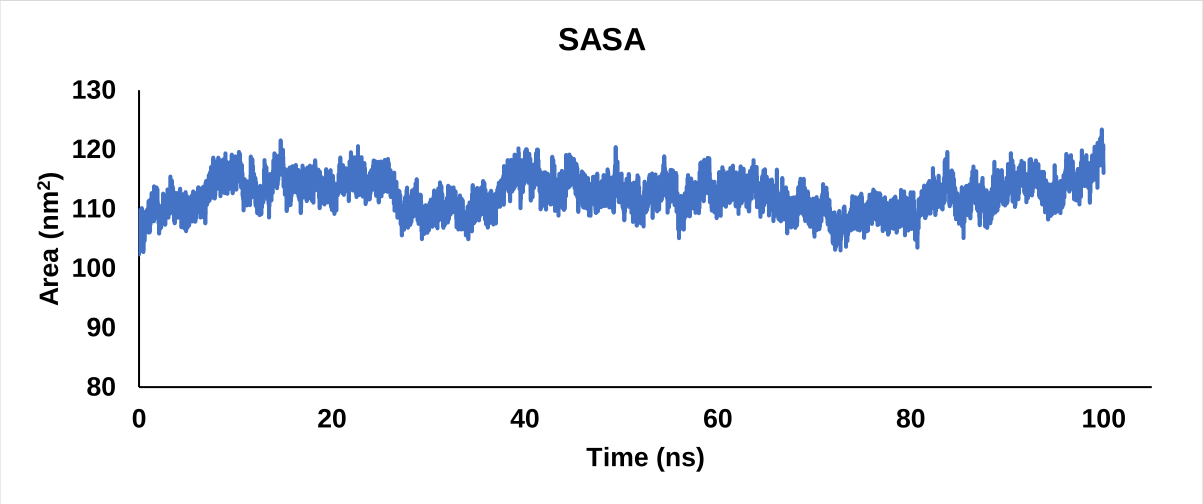

Figure S63. SASA curve of complex

Supplementary References:

Colomer-Winter, C., Lemos, J. A., & Flores-Mireles, A. L. (2019). Biofilm assays on fibrinogen-coated silicone catheters and 96-well polystyrene plates. Bio-protocol, 9(6), e3196-e3196.

Molteni, V.; He, X.; Nabakka, J.; Yang, K.; Kreusch, A.; Gordon, P.; Bursulaya, B.; Warner, I.; Shin, T.; Biorac, T., Identification of novel potent bicyclic peptide deformylase inhibitors. Bioorganic & medicinal chemistry letters 2004, 14 (6), 1477-1481.

O'Toole, G. A. (2011). Microtiter dish biofilm formation assay. JoVE (Journal of Visualized Experiments), (47), e2437.

Wiegand, I., Hilpert, K., & Hancock, R. E. (2008). Agar and broth dilution methods to determine the minimal inhibitory concentration (MIC) of antimicrobial substances. Nature protocols, 3(2), 163-175.
